# Pharmacophore Synergism in Diverse Scaffold Clinches in Aurora Kinase B

**DOI:** 10.3390/ijms232314527

**Published:** 2022-11-22

**Authors:** Vijay H. Masand, Sami A. Al-Hussain, Mithilesh M. Rathore, Sumer D. Thakur, Siddhartha Akasapu, Abdul Samad, Aamal A. Al-Mutairi, Magdi E. A. Zaki

**Affiliations:** 1 Department of Chemistry, Vidya Bharati Mahavidyalaya, Amravati 444602, Maharashtra, India; 2 Department of Chemistry, Faculty of Science, Imam Mohammad Ibn Saud Islamic University, Riyadh 11623, Saudi Arabia; 3 Department of Chemistry, RDIK and NKD College, Badnera, Amravati 444701, Maharashtra, India; 4 Curia Global, Springfield, MO 65807, USA; 5 Department of Pharmaceutical Chemistry, Faculty of Pharmacy, Tishk International University, Erbil 44001, Iraq

**Keywords:** aurora kinase B, QSAR, pharmacophore modeling

## Abstract

Aurora kinase B (AKB) is a crucial signaling kinase with an important role in cell division. Therefore, inhibition of AKB is an attractive approach to the treatment of cancer. In the present work, extensive quantitative structure–activity relationships (QSAR) analysis has been performed using a set of 561 structurally diverse aurora kinase B inhibitors. The Organization for Economic Cooperation and Development (OECD) guidelines were used to develop a QSAR model that has high statistical performance (R^2^_tr_ = 0.815, Q^2^_LMO_ = 0.808, R^2^_ex_ = 0.814, CCC_ex_ = 0.899). The seven-variable-based newly developed QSAR model has an excellent balance of external predictive ability (Predictive QSAR) and mechanistic interpretation (Mechanistic QSAR). The QSAR analysis successfully identifies not only the visible pharmacophoric features but also the hidden features. The analysis indicates that the lipophilic and polar groups—especially the H-bond capable groups—must be present at a specific distance from each other. Moreover, the ring nitrogen and ring carbon atoms play important roles in determining the inhibitory activity for AKB. The analysis effectively captures reported as well as unreported pharmacophoric features. The results of the present analysis are also supported by the reported crystal structures of inhibitors bound to AKB.

## 1. Introduction

The machinery for cell division, also known as mitosis, is completely regulated. Any irregularity or imperfect mitosis results in nondiploid DNA content, which ultimately causes cancer [1]. Researchers have therefore become interested in developing cancer chemotherapeutics that target centrosome maturation and separation, mitotic spindle assembly, chromosomal separation, and cytokinesis involving the participation of numerous important signaling kinases, including aurora, polo-like-kinase (Plk), and cyclin-dependent kinase (Cdk) [2,3]. The successful transition to mitosis depends on the aurora kinase family of serine/threonine kinases [4,5,6,7]. Since their discovery in 1995 and the initial detection of their expression in human cancer tissue in 1998 [2,5,7,8,9], these kinases have received a great deal of attention. This is due to their aberrant and excessive expression in a wide range of solid and liquid tumors, such as pancreatic, lung, liver, and breast tumors, as well as their oncogenic activity [2,4,5,7,8,9,10,11].

The aurora kinase family consists of three isoforms (A, B, and C), each of which differs in the length and amino acid composition of the N-terminal domain, but they share a common and conserved ATP binding site [2,12]. In order for the centrosome to mature, and for spindle assembly, meiosis, and metaphase spindle orientation to occur, aurora-A is essential [2,12]. In order to achieve precise chromosomal segregation and cytokinesis, aurora kinase B (AKB) is required [2,12]. Massive polyploidization and failure to bio-orientate chromosomes result from AKB inhibition [2,12]. Since aurora kinase C (AKC), which complements the activity of AKB, has received less attention to date, we decided to focus only on AKB in this investigation, due to a lack of data for AKC [12].

**Figure 1:**
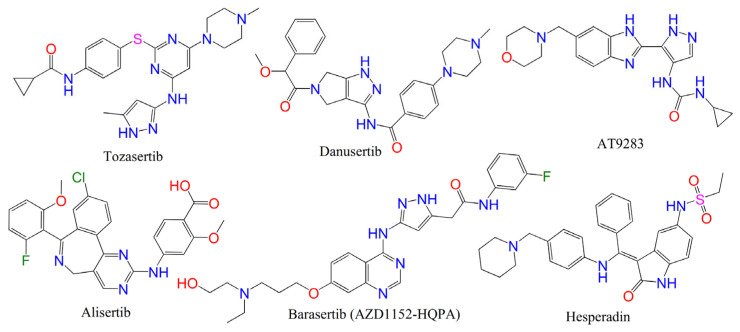
Structures of some known aurora inhibitors in different clinical trial stages.

Aurora kinases have been suggested as prospective targets for anticancer treatments due to their crucial function in controlling the cell cycle. At this time, none of the ATP-competitive inhibitors targeting AKB that are in clinical development (Figure 1) have been granted approval [4,5,13].

In these conditions, a quick and effective strategy to find AKB inhibitors is still a key goal for medicinal chemists. To fulfill this goal, there is a need to use modern methods such as computer-aided drug design (CADD) to reduce time, costs, trial-and-error procedures, and other required resources [14,15]. The vibrant and developing field of CADD is successful due to the result-oriented performance of molecular docking, QSAR, and its other branches [14,15,16]. In QSAR, a mathematical model is created to connect chemical descriptors (structural features) to a desired bioactivity profile using a wide range of machine learning techniques [17,18]. In a more pragmatic sense, QSAR allows one to prioritize compounds with desirable attributes for a subsequent (and presumably successful) biological evaluation [17,18,19]. Traditional QSAR concentrates on producing statistically significant models [17,18,19]. Previously, different researchers have reported QSAR models for AKB using different techniques. For example, Neaz et al. [20] reported a 3D-QSAR model for a dataset of forty-eight quinazoline derivatives possessing other heterocyclic rings. The developed model had a leave-one-out cross-validated correlation coefficient (Q2LOO) of 0.56. Another 3D-QSAR and molecular docking study of azaindole derivatives as AKB inhibitors was accomplished by Lan and co-workers [21]. The best developed QSAR model based on forty-one molecules had Q2LOO = 0.575. Likewise, Ashraf et al. [22] used a dataset of 57 acylureidoindolin derivatives to develop a 3D-QSAR model, which had Q2LOO = 0.641, and indicated that electrostatic and hydrophobic fields determine the activity of compounds. Thus, AKB has been the subject of QSAR research; however, the developed QSAR models find little usage due to a lack of generalizability, low predictive power, being based on small datasets comprising limited scaffolds, or a combination of these factors. Therefore, there is a need to develop a robust and balanced QSAR model based on a larger dataset, encompassing diverse structural scaffolds. Consequently, in the present work, a QSAR model has been developed that possesses high external predictive ability and extensive mechanistic interpretations supported by X-ray-resolved structures.

## 2. Results

As stated in Section 1, the focus was on developing a genetic algorithm–multilinear regression (GA–MLR) model with a combination of mechanistic interpretation and high predictive power. We have discovered several structural features in the current investigation. The recently constructed seven-parameter model and its statistical validation parameters are as follows.

Model A: pIC50 = 4.611 (±0.224) + 0.559 (±0.105) × fringNplaN4B + 0.436 (±0.11) × fsp3Csp2N5B + 0.253 (±0.038) × N_H_2B + 0.164 (±0.035) × fsp2Osp2C5B + 0.1 (±0.015) × da_lipo_5B − 0.317 (±0.056) × fringNC6B − 0.262 (±0.048) × fOringC6B.

Statistical parameters associated with model A: R^2^_tr_ = 0.815, RMSEtr = 0.468, MAEtr = 0.401, CCCtr = 0.898, s = 0.473, F = 277.836, R2cv (Q2LOO) = 0.808, RMSEcv = 0.477, MAEcv = 0.408, CCCcv = 0.895, Q2LMO = 0.807, R2Yscr = 0.016, Q2Yscr = −0.02, RMSEex = 0.446, MAEex = 0.373, R^2^_ex_ = 0.814, Q2-F1 = 0.811, Q2-F2 = 0.811, Q2-F3 = 0.833, CCC_ex_ = 0.900.

**Figure 2:**
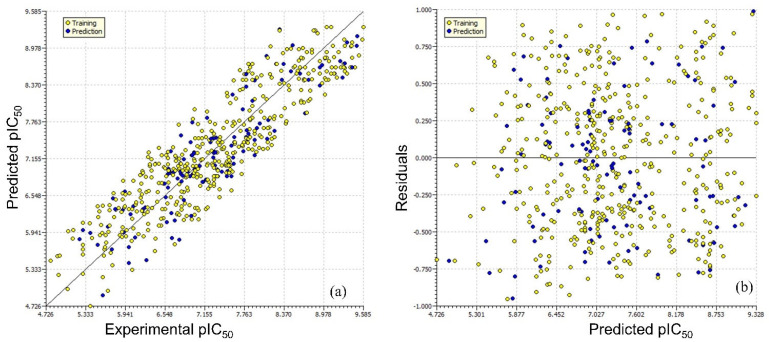

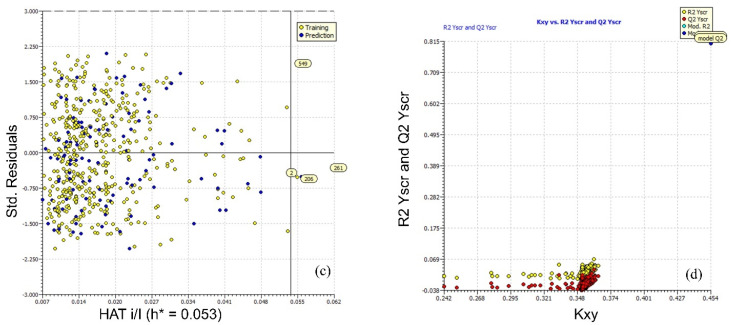
Different graphs related to model A: (**a**) experimental vs. predicted pIC50 (the solid line represents the regression line); (**b**) experimental vs. residuals; (**c**) Williams plot for applicability domain (the vertical solid line represents h* = 0.053 and horizontal dashed lines represent the upper and lower boundaries for applicability domain); (**d**) Y-randomization plot.

Model A is statistically robust, as shown by the high values of various statistical parameters, such as the coefficient of determination (R^2^_tr_) and cross-validated coefficient of determination for leave-one-out (R2cv or Q2LOO), the external coefficient of determination (R^2^_ex_), Q2-Fn and the Concordance Correlation Coefficient (CCC_ex_), etc., and the low values of lack-of-fit (LOF), root mean square error (RMSEtr), and mean absolute error (MAE). As a result, model A has high external predictive ability [23,24,25,26,27,28,29,30], is devoid of random correlations [31,32], and meets suggested threshold values for key parameters. The Supplementary Materials contain the formulae to determine these parameters. A Williams plot was used to evaluate the model’s applicability domain [33,34,35,36]. As a result, it complies with all the OECD-recommended standards and requirements for developing a valuable QSAR model. Different graphs associated with model A are depicted in Figure 2.

There are seven descriptors in model A, which have been calculated by PyDescriptor [37] and tabulated in Table 1. Of the seven descriptors, five descriptors, viz. fringNplaN4B, fsp3Csp2N5B, N_H_2B, fsp2Osp2C5B, and da_lipo_5B, have positive coefficients in model A, implying that increasing their value could lead to a better activity profile, whereas the reverse is true for the remaining two descriptors, fOringC6B and fringNC6B, which have negative coefficients in model A. Each molecular descriptor, which is a numeric representation of structural features [37,38,39], has correlations with different types of pharmacophoric features, which govern the inhibitory profile. However, it is to be noted that a single structural feature can neither explain nor fully determine the final biological activity (IC50) of a molecule. The biological activity IC50, etc., is an outcome of a combination of different structural features and some unknown factors. Some features enhance the desired pharmacological activity, whereas others are responsible for reversing it. It is believed that two or more pharmacophoric groups concomitantly decide the biological activity (pharmacophore synergism).

## 3. Discussion

Of the seven descriptors in model A, five descriptors, viz. fringNplaN4B, fsp3Csp2N5B, N_H_2B, da_lipo_5B, and fringNC6B, indicate the importance of different types of nitrogen atoms in determining the inhibitory activity for aurora kinase B. The same is true for carbon, which is present in four descriptors, viz. fsp3Csp2N5B, da_lipo_5B, fringNC6B, and fOringC6B. The relevance of oxygen is due to its presence in three descriptors, viz. fsp2Osp2C5B, da_lipo_5B, and fOringC6B. At the same time, it should be noted that the descriptors present in model A are highly interlinked; that is, increasing the value of one descriptor could significantly change the value of another descriptor. This leads to substantial changes in the biological profile of a molecule, pointing toward pharmacophore synergism, as molecular descriptors are mathematical representations of pharmacophores. For example, the values of descriptors fringNplaN4B and fringNC6B vary with the presence/absence of ring nitrogen atoms. Therefore, increasing the value of fringNplaN4B by escalating ring nitrogen atoms could also lead to a higher fringNC6B value. Therefore, in the present work, we have adopted an approach that involves the concomitant consideration of two or more molecular descriptors to explain the variance in the activity profile of matched molecular pairs (MMP). Accordingly, the molecular descriptors whose values have changed for MMP have been discussed concurrently with relevant examples in Section 3.

**da_lipo_5B:**

**Figure 3:**
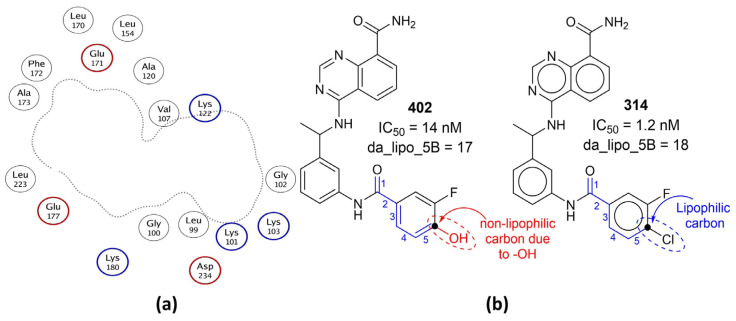
(**a**) A 2D depiction of active site of aurora kinase B (pdb: 4c2w). The dotted line represents the contour proximity of active site residues. Acidic and basic residues have been highlighted using red- and blue-colored circles. (**b**) Comparison of molecule 402 with 314 with respect to da_lipo_5B (blue-colored bonds and numbering).

The descriptor da_lipo_5B is simultaneously associated with two important aspects of a molecule: lipophilic character and H-bonding-capable (donor and acceptor) atoms. It is to be noted that, in the present work, a carbon atom is non-lipophilic while calculating da_lipo_5B, if oxygen or nitrogen is attached to it. The average value of da_lipo_5B for the top one hundred active molecules (IC50 = 0.26 to 4.3 nM) is 15.29, and the value for the least active one hundred molecules (IC50 = 611 to 16,000 nM) is 8.51. This reveals that the higher the number of lipophilic atoms within five bonds of a H-bond-capable atom, the higher the activity. This gives an initial impression that lipophilicity (mostly represented by logP [40]) is the only governing factor. However, the calculated logP (clogP), which represents molecular lipophilicity, has a weak correlation of 0.077 with pIC50, whereas da_lipo_5B has a value of 0.533. Therefore, the conditional occurrence of lipophilic atoms in the vicinity of H-bonding-capable atoms is a better choice. A plausible reason could be the composition of the active site of AKB, which consists of the persistent presence of lipophilic residues such as Gly, Leu, Val, Phe, etc., between the acidic or basic residues such as Glu, Asp, Lys [22]. This is why an aurora kinase B inhibitor also requires the presence of H-bond-capable atoms, preferably with separation by five bonds and the concomitant occurrence of lipophilic atoms in their vicinity. This observation is confirmed by the reported X-ray-resolved structure of aurora kinase B (pdb: 4c2w [41]) (see Figure 3).

The importance of da_lipo_5B highlights the significance of determining the numbers of donor cum acceptor atoms required to obtain better activity. The average value of donor cum acceptor atoms for the top one hundred active molecules (IC50 = 0.26 to 4.3 nM) is 3.21, and the value for the least active one hundred molecules (IC50 = 611 to 16,000 nM) is 2.24. A comparison of the following pairs of molecules as representative examples further highlights the importance of da_lipo_5B: 314 with 402 (see Figure 3), 355 with 347, 206 with 207, 103 with 101, 103 with 99, 61 with 142, 57 with 58, etc.

**fringNplaN4B:**

fringNplaN4B stands for the frequency of occurrence of planer nitrogen atoms exactly at four bonds from a ring nitrogen atom. If the same planer nitrogen atom is also present at ≤4 bonds from the same or any other ring nitrogen atom through any path, then it is excluded while calculating fringNplaN4B. The importance of fringNplaN4B is reflected by the fact that the most active 110 molecules with IC50 values ranging from 0.26 to 5.9 nM have one or more combinations of planer and ring nitrogen atoms. The reverse is true for less active molecules (IC50 = 16,000 to 611 nM), with some exceptions, such as molecule numbers 213, 73, 71, 66, 20, etc. Moreover, it was observed that replacing fringNplaN4B with its corresponding equivalents, fringNplaN3B and fringNplaN5B, for three and five bonds led to a reduction in the performance of model A (R^2^ = 0.770, for both). Moreover, fringNplaN3B and fringNplaN5B have a correlation of R = 0.084 and 0.028 with pIC50, respectively, whereas fringNplaN4B is a better choice as a descriptor, with R = 0.628.

However, at first sight, it appears that, individually, ringN (number of ring nitrogen atoms) or nplanN (number of planer nitrogen atoms) could be an alternative to fringNplaN4B. However, both have a weak correlation of 0.207 and 0.374 with pIC50, respectively. Moreover, a loss in the statistical performance of model A on replacing fringNplaN4B with ringN (R^2^ = 0.772) or nplanN (R^2^ = 0.770) again confirmed the importance of fringNplaN4B. Therefore, a combination of ring and planer nitrogen atoms separated exactly by four bonds is an important structural feature to obtain a better pIC50 for AKB.

**Figure 4:**
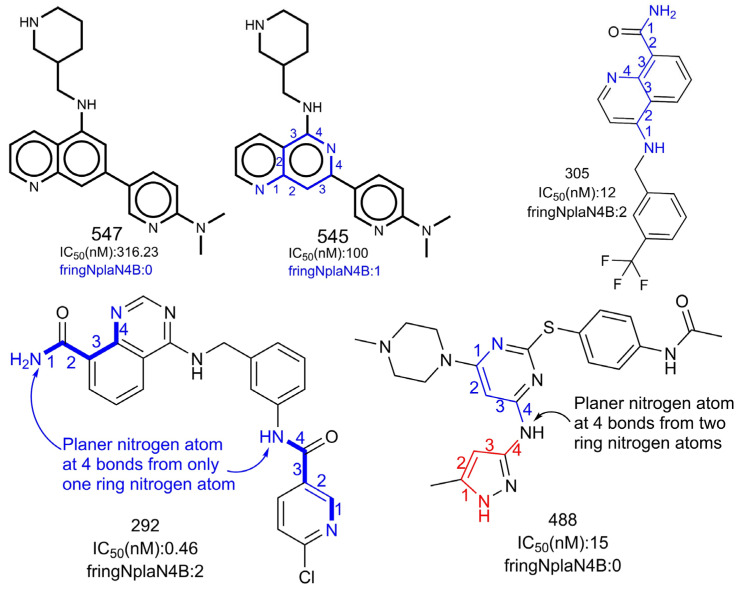
Representation of influence of fringNplaN4B on activity profile of AKB inhibitors. The numbers (blue/red) indicate the counting of number of bonds between ring and planer nitrogen.

A literature survey reveals that for pyrrolopyrazole derivatives, a substituted 3-aminopyrazole moiety is important due to its ability to interact with the hinge region of the ATP binding site [2]. The three nitrogen atoms of the N-C-N-N pattern present in 3-aminopyrazole are responsible for binding with the receptor [2]. Unfortunately, it appears that the reported pattern is exclusive to pyrrolopyrazole derivatives bearing a substituted 3-aminopyrazole moiety. Interestingly, the terminal nitrogen atoms of the N-C-N-N pattern are actually ring and planer nitrogen atoms, thereby suggesting the possible presence of fringNplaN4B. However, in many active molecules of the present dataset bearing a substituted 3-aminopyrazole moiety, the value of fringNplaN4B is zero; this is because the planer nitrogen of the N-C-N-N pattern is also present within ≤4 bonds of the other ring nitrogen atom. However, in several active molecules for AKB, fringNplaN4B is present due to other scaffolds (see Figure 4). In other words, instead of the N-C-N-N pattern or a substituted 3-aminopyrazole moiety, an emphasis on the simultaneous presence of planer and ring nitrogen atoms separated by four bonds in the molecule is a better strategy to enhance the inhibitory profile against AKB. Hence, the present work successfully identified a novel aspect of a reported pattern (N-C-N-N) and extended it for other scaffolds.

**N_H_2B:**

The positive coefficient for N_H_2B indicates that the presence of hydrogen in the vicinity of nitrogen is beneficial to increase the inhibitory activity for aurora kinase B. In many molecules, N_H_2B exists due to the direct attachment of a hydrogen atom to a nitrogen atom (N-H) or due to hydrogen atoms bonded to carbon atoms adjacent to nitrogen (N-CHn fragment). N_H_2B favors two important structural features that could lead to a better inhibitory profile: (1) the presence of polar hydrogen atoms as N-H or N-CHn fragments; (2) steric hindrance or bulkiness in the vicinity of nitrogen atoms, because hydrogen is the smallest among all the elements. The lesser the bulkiness around nitrogen atoms, the better the inhibitory profile. These two structural features in combination allow the polar interactions or H-bond formation between the ligand and the receptor. This observation, and the significance of N_H_2B as well as da_lipo_5B, is confirmed by the two forms of the ligand VX-680 (molecule number 14) in the pdb 4b8m [42].

**Figure 5:**
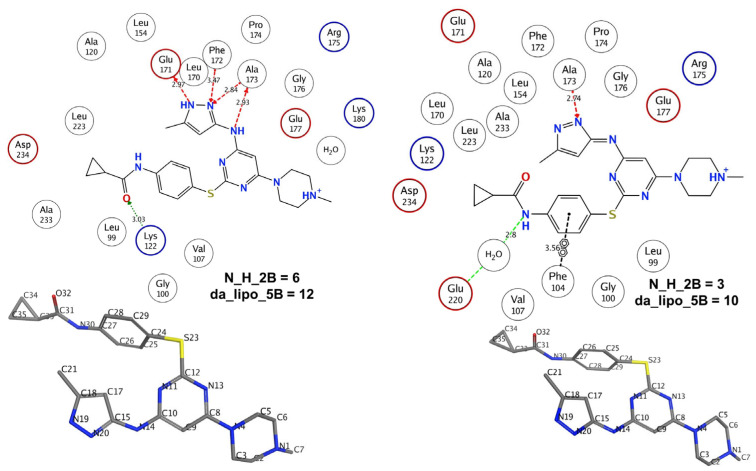
Pictorial representation of N_H_2B using VX-680 (pdb 4b8m) as an example.

The ligand VX-680 exists in two different forms, labeled as TA and TB in the present work, in the two chains of pdb 4b8m. From Figure 5 and Table 2, it is clear that the TA form consists of a higher number of hydrogen atoms than TB, especially in the vicinity of nitrogen atoms. This led to different values for N_H_2B for the two forms (see Figure 5). The form TA, having a higher N_H_2B value, has a higher number of interactions with the receptor, because the additional hydrogen atoms attached to the nitrogen atoms of the pyrazole (designated as N19 and N20) ring and aminopyrimidine (designated as N14) are responsible for H-bond interactions with Glu171, Phe172, and Ala173 (see Table 2). Meanwhile, these interactions are absent for TB, even though the respective atoms N19 and N14 of TB are more proximate to receptor atoms. The TB form has only one prominent interaction with the receptor due to the nitrogen (designated as N20) of the pyrazole ring in the form of a H-bond with Ala173.

**Figure 6:**
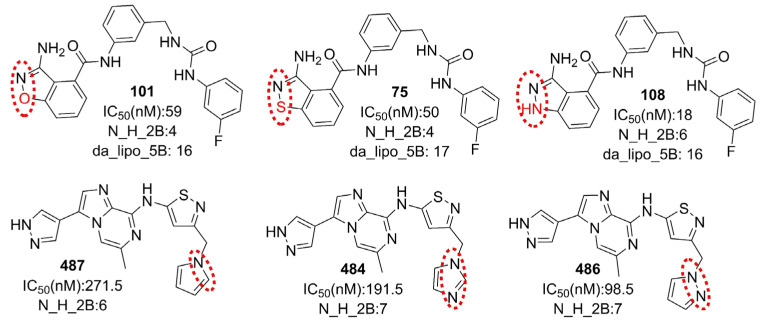
Representative examples to understand N_H_2B.

The following comparisons of molecules further highlight the importance of N_H_2B (see Figure 6): 108 with 75 and 101, 486 with 487 and 484, and 148 with 144, to list a few. A simple analysis of these examples indicates that the presence of a pyrazole ring leads to a better IC50 for a molecule (see Figure 6). However, it has a negative correlation (R = −0.177) with pIC50. A plausible reason appears from the present work suggesting that H-bond-capable polar groups are more suitable near the periphery of a molecule, rather than a pyrazole ring, to achieve good interactions with the receptor.

**fsp3Csp2N5B:**

The descriptor fsp3Csp2N5B is associated with two features, viz. sp2-hybridized nitrogen and sp3-hybridized carbon atoms. As it has a positive coefficient in model 1, increasing the numbers of such atoms favors the augmentation of pIC50. At the same time, increasing fsp3Csp2N5B could influence the values of da_lipo_5B and N_H_2B, as these descriptors are associated with carbon and nitrogen too. Therefore, it indicates that pharmacophore synergism determines the final inhibitory ability of a molecule for AKB. This is clearly reflected when molecule 435 is compared with molecule 438.

**Figure 7:**
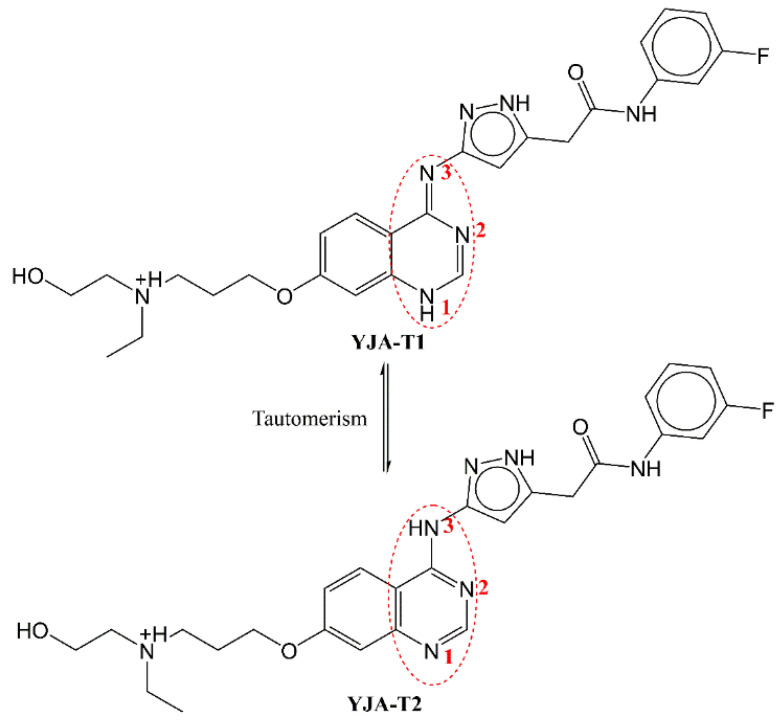
Tautomeric forms of ligand YJA (pdb 4c2v). The red colored numbers have been used for indication of nitrogen atoms involved in tautomerism.

The pdb 4c2v contains two different tautomeric forms of ligand YJA in two different chains, A and B. The influence of fsp3Csp2N5B along with N_H_2B is observed for the two tautomeric forms of co-crystallized ligand ‘YJA’ in the pdb 4c2v [41]. The two tautomeric forms show that YJA-T1 and YJA-T2 (see Figure 7) of ligand YJA have different values for fsp3Csp2N5B and N_H_2B (see Table 3). The online tautomer generator from Chemaxon (https://disco.chemaxon.com/calculators/demo/plugins/tautomers/, accessed on 28 October 2022) indicates that the ligand YJA can exist in seven different tautomeric forms. However, only two tautomeric forms, YJA-T1 and YJA-T2, predominate, with approximately 16 and 84 percent, respectively. The rest of the tautomeric forms have less than a 0.1% probability of existence.

**Figure 8:**
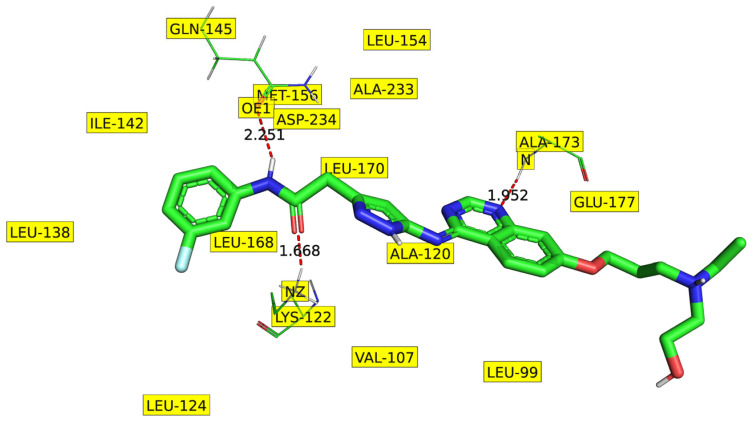

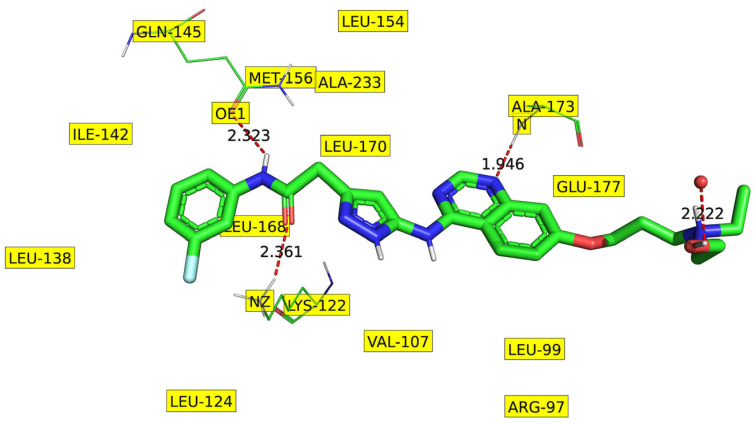
Depiction of prominent interactions of YJA-T1 and T2 with the receptor (pdb: 4c2v).

A comparison of the interactions of YJA-T1 and YJA-T2 with the receptor and the solvent indicates that the two forms have established H-bonds with the similar amino acid residues of the receptor but with different distances (see Figure 8). The YJA-T2 has an additional H-bond with the solvent (HOH2108). Moreover, it has a higher number of interactions with the receptor and the solvent (H_2_O) within 5 Å compared to YJA-T1. Thus, the increased value of fsp3Csp2N5B and N_H_2B for these two tautomeric forms correlates with a higher number of receptor atoms in the vicinity, which ultimately leads to an augmented number of interactions. Additional details related to the interactions of YJA-T1 and YJA-T2 with the receptor are available in Table S1 in the Supplementary Materials.

**fsp2Osp2C5B:**

**Figure 9:**
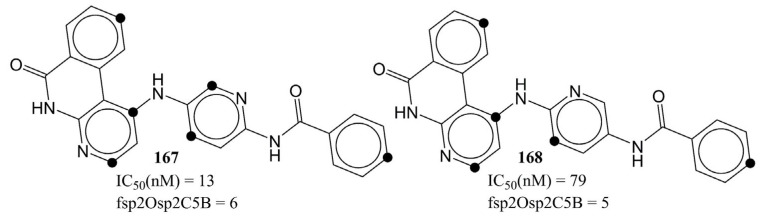
Representation of fsp2Osp2C5B using molecule numbers 167 and 168 as representative examples. The black circle represents the sp2-hybridized carbon at five bonds from sp2-hybridized oxygen.

The molecular descriptor fsp2Osp2C5B underlines the influence of a specific combination of sp2-hybridized carbon with sp2-hybridized oxygen in determining the inhibitory profile for AKB. The positive coefficient for fsp2Osp2C5B indicates that increasing such a combination of oxygen and carbon could lead to a better inhibitory profile. In the present dataset, there are 426 molecules with the presence of at least one such combination of oxygen and carbon. Likewise, the 200 most active molecules with IC50 values in the range of 0.26 to 24 nM, except molecule numbers 36 and 469, also possess fsp2Osp2C5B >1. A comparison of molecule number 167 with 168 further strengthens this observation (see Figure 9).

A closer analysis revealed that the sp2-hybridized carbon with sp2-hybridized oxygen, required for the existence of fsp2Osp2C5B are, in general, aromatic carbon atoms and oxygen of the carbonyl group, especially the amide group, respectively. This further highlights the importance of aromatic rings—and in turn lipophilic atoms—as aromatic carbons are mostly lipophilic in nature. The need for an amide group in conjugation point outs the necessity of a polar group to enhance the interactions with the receptor. The two tautomeric forms of YJA-T1 and T2 possess such a combination and it results in enhanced interactions with the receptor (see Figure 8). Obviously, a sp2-hybridized carbon atom will be at a respective distance of three and five bonds from the nitrogen and oxygen atoms of the same amide group; therefore, we also checked the importance of famdNsp2C3B (frequency of occurrence of sp2-hybridized carbon atoms exactly at three bonds from amide nitrogen atoms). It was observed that fsp2Osp2C5B and famdNsp2C3B have a correlation of 0.64 and 0.58, respectively, with pIC50. Therefore, fsp2Osp2C5B is a better choice to be considered for future optimizations and activity predictions.

**fOringC6B:**

**Figure 10:**
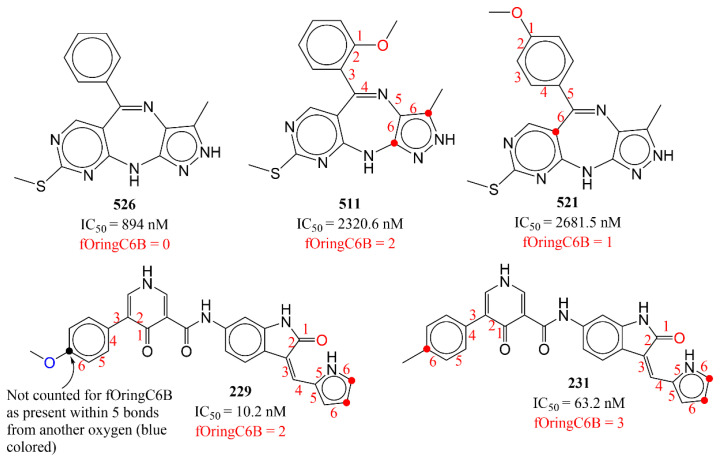
Representative examples for fOringC6B. The numbers (red) indicate the counting of number of bonds between ring carbon and oxygen atom.

The descriptor fOringC6B is associated with the simultaneous and conditional occurrence of polar (oxygen) and lipophilic characters (ring carbons) with an exact separation by six bonds. If a ring carbon is also present within five or less bonds of any other oxygen atom, then it is omitted while calculating fOringC6B. The molecular descriptor fOringC6B has a negative coefficient in model 1, which means that a higher number of such carbon atoms could reduce the inhibitory profile of a molecule for AKB. This is confirmed when the following pairs of molecules are compared: 526 with 511, 526 with 521, 204 with 205, 229 with 231, 477 with 485, and 256 with 257. The descriptor has been depicted in Figure 10. The red dots indicate the ring carbons, which contribute to fOringC6B at exactly six bonds from the oxygen atom. The six bonds separating such carbon and oxygen atoms have been labeled with numbers.

It appears that reducing the number of ring carbon atoms is a feasible solution to achieve a lower value of fOringC6B, but this will affect negatively other descriptors, viz. da_lipo_5B, fsp2Osp2C5B. Instead, a solution is to reduce the number of oxygen atoms or alternatively increase their presence within five or less bonds of ring carbon atoms. The second solution is observed in the case of molecule number 229. The additional -OCH3 led to a decreased value of fOringC6B, because, while calculating fOringC6B, if a ring carbon atom was simultaneously present within six bonds of two or more oxygen atoms, it was excluded.

**fringNC6B:**

**Figure 11:**
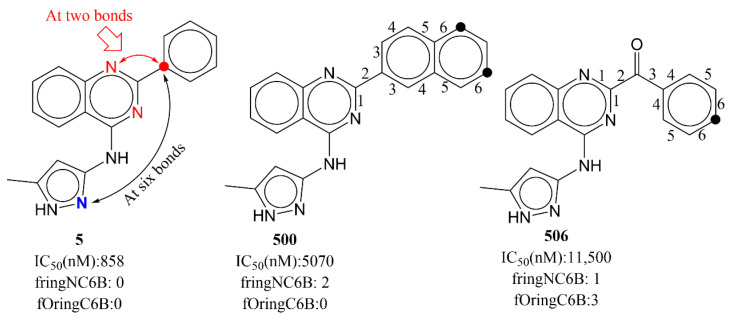
Depiction of fringNC6B using molecule numbers 5, 500, and 506 as representative examples. The carbon present at six bonds from ring nitrogen has been depicted using black dots. The numbers (black) indicate the counting of number of bonds between ring nitrogen and carbon.

The molecular descriptor fringNC6B provides crucial information about the upper limit for separation required between the lipophilic (carbon atoms) and polar (nitrogen atoms) moieties to achieve a better activity profile. While calculating fringNC6B, if a carbon atom is also present within five bonds of any other ring nitrogen, then it is omitted. If a carbon atom is present exactly at a distance of six bonds from a ring nitrogen atom, then it contributes negatively; therefore, such a combination should be avoided. Reducing the bond gap between carbon and ring nitrogen is a feasible and justified solution, as other descriptors, viz. da_lipo_5B and fsp3Csp2N5B, also indicate the same. As stated earlier, a plausible reason for this could be the active site of AKB (see Figure 11). The influence of fringNC6B on activity is confirmed when following pairs of molecules are compared: 5 with 500, 5 with 506, 374 with 406, 507 with 514, to list a few.

As stated earlier, the descriptors present in model A are entangled. Therefore, changing one descriptor could result in changes in other descriptors. For example, the descriptors fringNplaN4B and fringNC6B indicate the importance of ring nitrogen atoms. The fringNplaN4B has a positive correlation with pIC50 but fringNC6B has the opposite relation. Therefore, increasing the value of fringNplaN4B by escalating the ring nitrogen atoms could also lead to a higher fringNC6B value. Hence, a balance of the appropriate number and types of nitrogen, carbon, and oxygen could lead to significant inhibitory activity for aurora kinase B.

## 4. Materials and Methods

In this work, we adhered to the OECD’s and other researchers’ suggested standards and recommendations [17,18,19,32,43,44] for a successful QSAR analysis. The various procedures for creating a model included meticulous dataset selection, data curation, 3D structure production for all molecules, computation and trimming of molecular descriptors, model creation and extensive validation, and mechanistic interpretation [45,46]. To eliminate bias and ensure proper model validation, these stages were carried out one at a time.

### 4.1. Selection of Dataset

**Figure 12:**
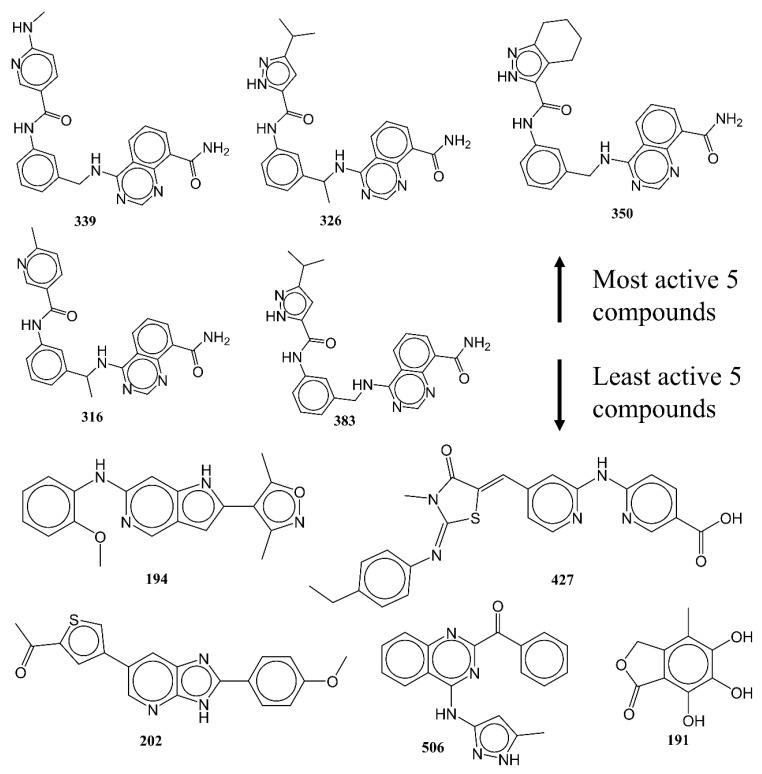
Representative examples from the selected dataset (five most active and five least active molecules).

The success and efficacy of a QSAR analysis in the drug discovery pipeline are significantly influenced by the size, composition, and structural diversity of the selected dataset used for the analysis [17,18,19,32,43,44]. As a result, a sizable dataset of 3398 reported AKB ligands was downloaded from BindingDB (https://www.bindingdb.org/bind/index.jsp, accessed on 14 January 2022). The dataset was then reduced to 561 molecules only after duplicates (average value for duplicates), salts, metal derivatives, rule-of-five violators, molecules with undefinable Ki values, etc., were eliminated during data curation [47]. The condensed dataset still included a variety of molecules, such as stereoisomers, positional and chain isomers, various heterocyclic and aromatic scaffolds, etc. Thus, it covered a broad chemical space. The experimental IC50 ranged from 0.26 to 16,000 nM. The experimental IC50 values were converted to pIC50 for a better QSAR analysis (−log_10_IC50). Figure 12 and Table 4 comprise some molecules that are very active and those that are least active, to help the readers to understand the structural variation present in the dataset.

### 4.2. Calculation of Molecular Descriptors and Objective Feature Selection (OFS)

The next step involved applying the proper methodology to convert SMILES notations into 3D-optimized structures. OpenBabel 3.1 [48] was used to translate SMILES to SDF for this. Then, utilizing PM3 as a force field for structure optimization and partial charge assignment, SDF was converted to MOL2 using MOPAC [49] 2016. After this, PyDescriptor [37] and PaDEL [50], which together offered more than 40,000 molecular descriptors for each molecule, were used for molecular descriptor calculation. Although using a large number of molecular descriptors increases the likelihood that a QSAR analysis will be effective, with a balance of predictive and mechanistic interpretation abilities, it also raises the risk of overfitting due to noisy redundancy in the descriptors or chance correlations. As a result, OFS was carried out using QSARINS 2.2.4 [51], which eliminated molecular descriptors that were nearly constant (for 90% of molecules) and highly inter-correlated (|R| > 0.90). After extensive OFS, only 1150 descriptors were finally included in the reduced set of molecular descriptors, but they nevertheless covered a wide descriptor space because they included fingerprints, charged-based, 1D to 3D, and a good number of atom-pair descriptors. The likelihood of a mechanistic interpretation of the model increased because a significant portion of the descriptors could be readily interpreted in terms of structural traits.

### 4.3. Splitting the Dataset into Training and External Sets and Subjective Feature Selection (SFS)

SFS is one of the most important steps in the QSAR model-building process that involves choosing the right feature selection technique with an adequate number and set of molecular descriptors. Before developing the QSAR model, the dataset was randomly divided into a training set (80%, or 449 molecules) and a prediction set (20%, or 112 molecules), to allow for proper training and validation of the model. In order to eliminate bias, reduce information leakage [32], confirm the model’s external predictive ability to predict for molecules other than the training set, and to improve the composition of the training and prediction sets, the dataset was randomly divided at a ratio of 80:20. The selection of molecular descriptors was done using the training set only. The prediction set, also known as the test set or external set, was used exclusively for judging the external predictive ability of the model.

**Figure 13:**
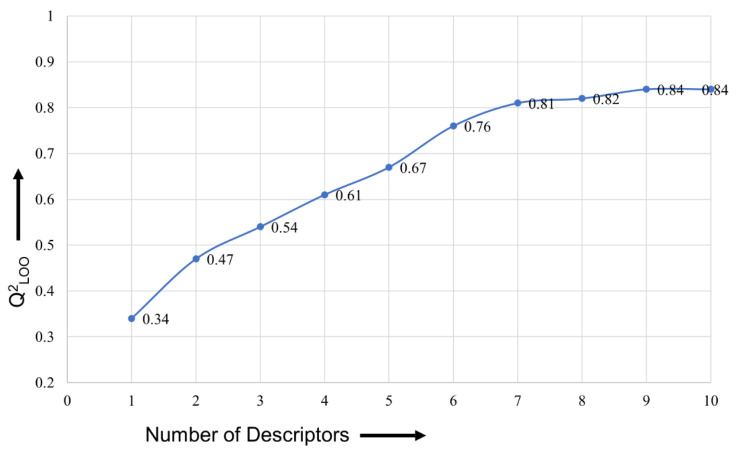
Plot of number of descriptors against leave-one-out coefficient of determination (Q2LOO) to identify the optimum number of descriptors.

To prevent over- and underfitting, the QSAR model must have an ideal number of molecular descriptors (variables). Consequently, the ideal number of descriptors for the model was identified using a straightforward graphical (or breaking point) method [45,46,52]. The value of Q2LOO typically increases considerably when a new variable (molecular descriptor) is added in stages to an MLR model until the desired elevation is reached. After this, the value of Q2LOO increases slightly or negligibly. As a result, the number of molecular descriptors that match the elevation point is ideal for creating a QSAR model. A graph of this is shown in Figure 13. The last elevation point in Figure 13 corresponds to seven molecular descriptors. Therefore, the genetic algorithm (GA) in combination with multi-regression (GA–MLR) method, using QSARINS 2.2.4, was used for the exhaustive search to identify seven molecular descriptors to develop the QSAR model. For GA–MLR, Q2LOO was used as the fitness parameter.

### 4.4. Building Regression Model and Its Validation

Different combinations of various molecular descriptors were eventually found during the search for seven molecular descriptors for the QSAR model using GA–MLR. However, due to the statistical performance and the satisfaction of adhering to strict parameters and criteria, which have been recommended [17,18,19,23,27,32,33,44,45,46,52,53,54,55,56,57] by a significant number of researchers, only one combination of molecular descriptors was chosen. The following threshold values and conditions were used to select the model:

R^2^_tr_ ≥ 0.6, Q2LOO ≥ 0.5, Q2LMO ≥ 0.6, R^2^ > Q2LOO, R^2^_ex_ ≥ 0.6, RMSEtr < RMSEcv, ΔK ≥ 0.05, CCC ≥ 0.80, Q2-Fn ≥ 0.60, r2m ≥ 0.5, (1-r2/ro2) < 0.1, 0.9 ≤ k ≤ 1.1 or (1-r2/r′o2) < 0.1, 0.9 ≤ k′ ≤ 1.1, | ro2− r′o2| < 0.3, RMSEex, MAEex, R^2^_ex_, Q2F1, Q2F2, Q2F3, and low R2Yscr, RMSE and MAE.

The model’s application domain must be identified for additional validation. In order to assess the application domain of the QSAR model, we employed a Williams plot (standardized residuals vs. hat values).

## 5. Conclusions

In relation to different features influencing the inhibitory activity for AKB, the present analysis successfully highlighted the significance of different types of atoms, groups, patterns, and tautomerism. Additionally, it emphasized the significance of specific patterns of atoms of different hybridization and their inter-relations in determining the final activity. The conditional presence of lipophilic (carbon) atoms or groups with respect to nitrogen atoms was also successfully recognized by model A as being beneficial for obtaining higher inhibitory for AKB. The present work, for the first time, pointed out the role played by tautomerism for AKB inhibitors. Model A performed statistically well, which was indicative of its strong external prediction power. As the current work successfully recognized both previously described and novel pharmacophoric properties associated with AKB inhibition, the results are of immense use throughout the drug discovery pipeline for the development of lead/drug candidates against AKB.

## Figures and Tables

**Table 1:** Different molecular descriptors present in model A and their descriptions.

Molecular Descriptor	Description
fringNplaN4B	Frequency of occurrence of planer nitrogen atoms exactly at 4 bonds from ring nitrogen atom
fsp3Csp2N5B	Frequency of occurrence of sp2-hybridized nitrogen atoms exactly at 5 bonds from sp3-hybridized carbon atoms
N_H_2B	Total number of nitrogen atoms present within 2 bonds from hydrogen atoms
fsp2Osp2C5B	Frequency of occurrence of sp2-hybridized carbon atoms exactly at 5 bonds from sp2-hybridized oxygen atoms
da_lipo_5B	Total number of lipophilic atoms present within 5 bonds from H-bond donor cum acceptor atoms
fOringC6B	Frequency of occurrence of ring carbon atoms exactly at 6 bonds from oxygen atoms
fringNC6B	Frequency of occurrence of carbon atoms exactly at 6 bonds from ring nitrogen atoms

**Table 2:** Distances of different atoms of TA and TB forms of VX-680 (molecule number 14) from the receptor atoms (pdb 4b8m).

TA Form				TB Form			
Residue	Residue Atom	Ligand Atom	Distance	Residue	Residue Atom	Ligand Atom	Distance
GLU171	O	N19	2.97	GLU171	O	N19	2.74
PHE172	CA	N20	3.47	PHE172	CA	N20	3.52
ALA173	N	N20	2.84	ALA173	N	N20	2.74
ALA173	O	N14	2.93	ALA173	O	N14	2.91
HOH2005	O	N13	3.32	HOH2005	O	N30	2.80

**Table 3:** A comparison of two tautomeric forms, YJA-T1 and YJA-T2.

Tautomer with Descriptor Value	H-Bonds Formed with Distance (Å) with Angle (Donor–Hydrogen–Acceptor) (Cut-Off: 5 Å)	List of Receptor Heavy Atoms within 5 Å of N3 atom of Ligand (Residue–Atom–Distance in Å)	List of Receptor Heavy Atoms within 5 Å of N1 Atom of Ligand (Residue–Atom–Distance in Å)
YJA-T1 fsp3Csp2N5B = 0 N_H_2B = 6 fsp2Osp2C5B = 3	LYS122 at 1.668 with 159.8^°^, GLN145 at 2.251 with 142.4, ALA173 at 1.952 with 163.9^°^	VAL107-CB-4.672, VAL107-CG1-4.351, VAL107-CG2-4.419, LU177-OE2-4.842, LEU223-CG-4.608, LEU223-CD1-3.627, LEU223-CD2-4.406	LEU99-CD1-4.259, ALA120-CB-4.501, GLU171-C-4.888, GLU171-O-4.058, PHE172-N-4.808, PHE172-CA-3.818, PHE172-C-3.832, PHE172-CB-4.641, PHE172-CG-4.403, PHE172-CD1-3.550, PHE172-CE1-4.156, ALA173-N-2.936, ALA173-CA-3.743, ALA173-C-4.208, ALA173-O-3.930, ALA173-CB-3.623, LEU223-CD1-4.121
YJA-T2 fsp3Csp2N5B = 1 N_H_2B = 7 fsp2Osp2C5B = 3	LYS122 at 2.361 with 157.8^°^, GLN145 at 2.323 with 115.7^°^, ALA173 at 1.946 with 174.4^°^, HOH2108 2.222 with 106.7^°^	PHE104-CG-4.358, PHE104-CD2-3.203, PHE104-CE2-3.058, PHE104-CZ-4.124, VAL107-CB-4.591, VAL107-CG1-4.413, VAL107-CG2-4.142, LEU223-CD1-4.047, LEU223-CD2-4.948	LEU99-CD2-3.977, ALA120-CB-4.707, GLU171-C-4.734, GLU171-O-3.872, PHE172-N-4.690, PHE172-CA-3.669, PHE172-C-3.814, PHE172-CB-4.567, PHE172-CG-4.418, PHE172-CD1-3.618, PHE172-CE1-4.265, ALA173-N-2.953, ALA173-CA-3.799, ALA173-C-4.271, ALA173-O-3.915, ALA173-CB-3.635, LEU223-CD1-4.165

**Table 4:** SMILES notation, IC50 (nM), and pIC50 (M) of five most and least active molecules of the selected dataset.

Mol ID	SMILES	IC50 (nM)	pIC50 (M)
339	O=C(Nc1cc(CNc2ncnc3c(C(=O)N)cccc23)ccc1)c1cnc(NC)cc1	0.26	9.585
326	O=C(Nc1cc(C(Nc2ncnc3c(C(=O)N)cccc23)C)ccc1)c1[nH]nc(C(C)C)c1	0.27	9.569
350	O=C(Nc1cc(CNc2ncnc3c(C(=O)N)cccc23)ccc1)c1[nH]nc2c1CCCC2	0.3	9.523
316	O=C(Nc1cc(C(Nc2ncnc3c(C(=O)N)cccc23)C)ccc1)c1cnc(C)cc1	0.32	9.495
383	O=C(Nc1cc(CNc2ncnc3c(C(=O)N)cccc23)ccc1)c1[nH]nc(C(C)C)c1	0.33	9.481
191	O=C1OCc2c(C)c(O)c(O)c(O)c12	8690	5.061
506	O=C(c1nc(Nc2n[nH]c(C)c2)c2c(n1)cccc2)c1ccccc1	11,500	4.939
202	O=C(C)c1scc(-c2cnc3[nH]c(-c4ccc(OC)cc4)nc3c2)c1	12,100	4.917
427	O=C(O)c1cnc(Nc2nccc(/C=C\3/C(=O)N(C)/C(=N/c4ccc(CC)cc4)/S/3)c2)cc1	12,505.05	4.903
194	O(C)c1c(Nc2ncc3c([nH]c(-c4c(C)onc4C)c3)c2)cccc1	16,000	4.796

## Data Availability

Data are contained within the article and Supplementary Materials.

## Supplementary Materials

The following supporting information can be downloaded at: https://www.mdpi.com/article/10.3390/ijms232314527/s1.

## Abbreviations

SMILES	Simplified molecular-input line-entry system
GA	Genetic algorithm
MLR	Multiple linear regression
QSAR	Quantitative structure−activity relationship
WHO	World Health Organization
OLS	Ordinary least squares
QSARINS	QSAR Insubria
OECD	Organization for Economic Cooperation and Development
