# Supplementary material for: Pharmacophore Synergism in Diverse Scaffold Clinches in Aurora Kinase B
Source: Int J Mol Sci. 2022 Nov 22;23(23):14527. doi: 10.3390/ijms232314527 (PMC9739353; doi:10.3390/ijms232314527)
Supplement: Supplementary file 1 [file ijms-23-14527-s001.zip › Supplementary Material.pdf]

1 148

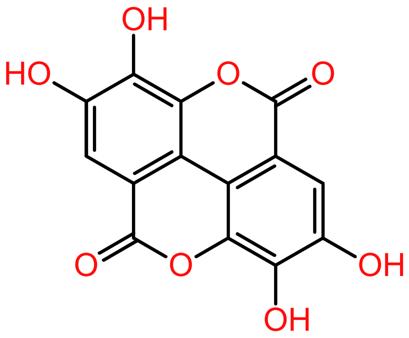

IC50(nM):2500
fringNplaN4B:0
fsp3Csp2N5B:0
N_H_2B:0
fsp2Osp2C5B:0
da_lipo_5B:6
fringNC6B:0
fOringC6B:0

2 3666

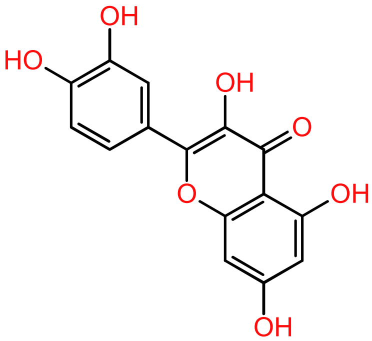

IC50(nM):910
fringNplaN4B:0
fsp3Csp2N5B:0
N_H_2B:0
fsp2Osp2C5B:3
da_lipo_5B:7
fringNC6B:0
fOringC6B:0

3 12922

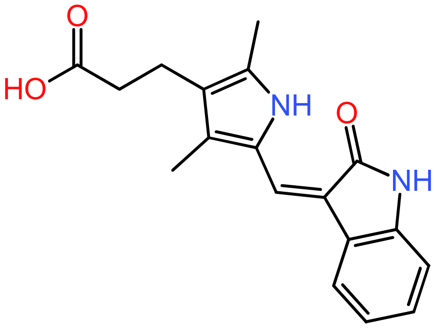

IC50(nM):254.5
fringNplaN4B:0
fsp3Csp2N5B:0
N_H_2B:2
fsp2Osp2C5B:3
da_lipo_5B:13
fringNC6B:0
fOringC6B:0

4 209968

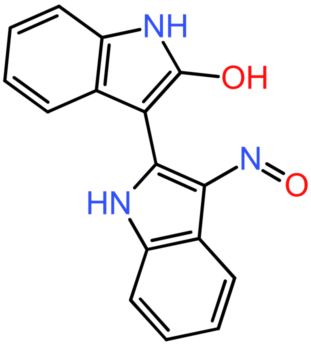

IC50(nM):1150.5
fringNplaN4B:0
fsp3Csp2N5B:0
N_H_2B:2
fsp2Osp2C5B:4
da_lipo_5B:11
fringNC6B:0
fOringC6B:1

5 298591

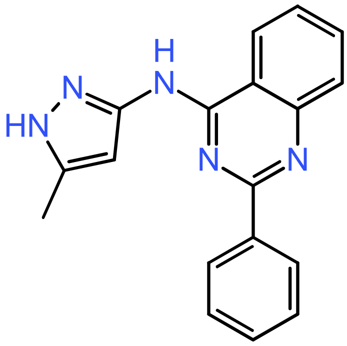

IC50(nM):858
fringNplaN4B:0
fsp3Csp2N5B:0
N_H_2B:3
fsp2Osp2C5B:0
da_lipo_5B:10
fringNC6B:0
fOringC6B:0

6 341698

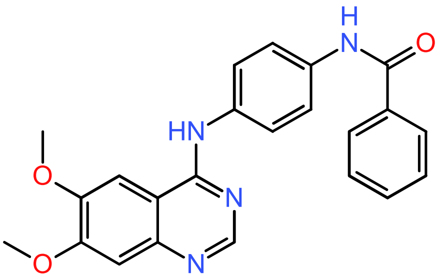

IC50(nM):626
fringNplaN4B:0
fsp3Csp2N5B:1
N_H_2B:4
fsp2Osp2C5B:3
da_lipo_5B:13
fringNC6B:2
fOringC6B:1

7 353408

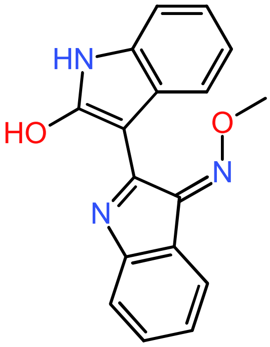

IC50(nM):3000
fringNplaN4B:0
fsp3Csp2N5B:1
N_H_2B:1
fsp2Osp2C5B:0
da_lipo_5B:7
fringNC6B:0
fOringC6B:1

8 353452

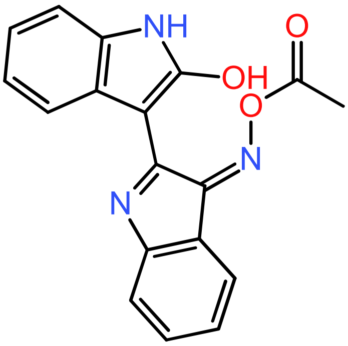

IC50(nM):2100
fringNplaN4B:0
fsp3Csp2N5B:0
N_H_2B:1
fsp2Osp2C5B:0
da_lipo_5B:7
fringNC6B:1
fOringC6B:1

9 361860

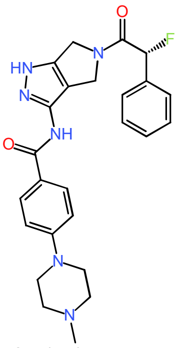

IC50(nM):300
fringNplaN4B:0
fsp3Csp2N5B:0
N_H_2B:6
fsp2Osp2C5B:3
da_lipo_5B:7
fringNC6B:1
fOringC6B:1

10 361877

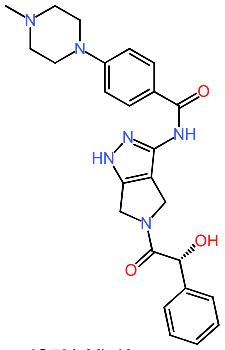

IC50(nM):48
fringNplaN4B:0
fsp3Csp2N5B:0
N_H_2B:6
fsp2Osp2C5B:3
da_lipo_5B: 12
fringNC6B:1
fOringC6B:0

11 361909

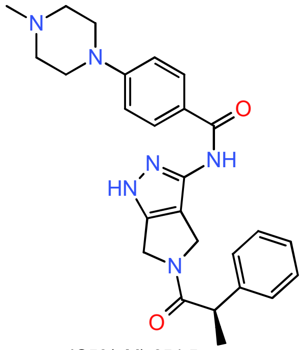

IC50(nM):654.5
fringNplaN4B:0
fsp3Csp2N5B:0
N_H_2B:6
fsp2Osp2C5B:3
da_lipo_5B: 7
fringNC6B:1
fOringC6B:1

12 361959

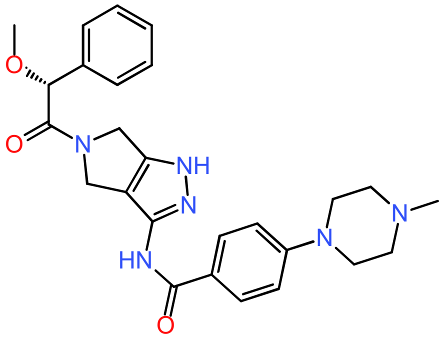

IC50(nM):630
fringNplaN4B:0
fsp3Csp2N5B:0
N_H_2B:6
fsp2Osp2C5B:3
da_lipo_5B: 6
fringNC6B:1
fOringC6B:0

13 369152

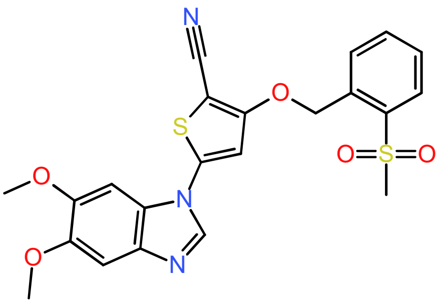

IC50(nM):1807.273333
fringNplaN4B:0
fsp3Csp2N5B:1
N_H_2B:2
fsp2Osp2C5B:1
da_lipo_5B: 0
fringNC6B:1
fOringC6B:0

14 428508

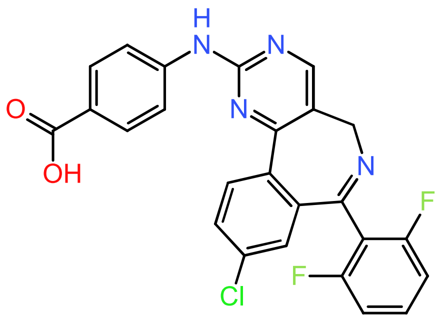

IC50(nM):2044.43
fringNplaN4B:0
fsp3Csp2N5B:0
N_H_2B:3
fsp2Osp2C5B:0
da_lipo_5B: 9
fringNC6B:1
fOringC6B:0

15 429302

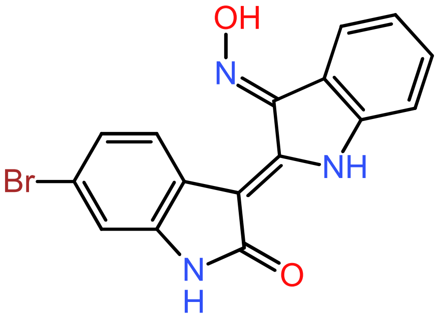

IC50(nM):451.5
fringNplaN4B:0
fsp3Csp2N5B:0
N_H_2B:3
fsp2Osp2C5B:4
da_lipo_5B: 12
fringNC6B:0
fOringC6B:1

16 436277

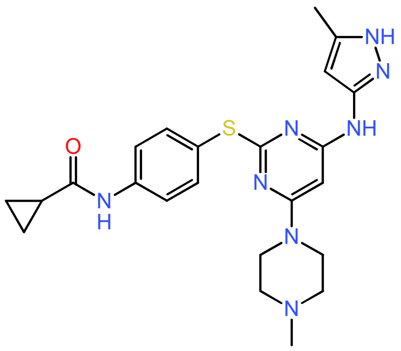

IC50(nM):66.73364103
fringNplaN4B:0
fsp3Csp2N5B:1
N_H_2B:6
fsp2Osp2C5B:2
da_lipo_5B: 12
fringNC6B:1
fOringC6B:1

17 436278

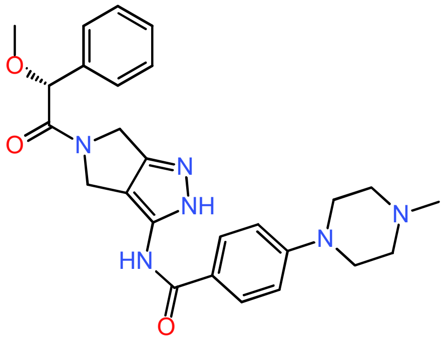

IC50(nM):89
fringNplaN4B:0
fsp3Csp2N5B:0
N_H_2B:6
fsp2Osp2C5B:3
da_lipo_5B: 6
fringNC6B:1
fOringC6B:0

18 449128

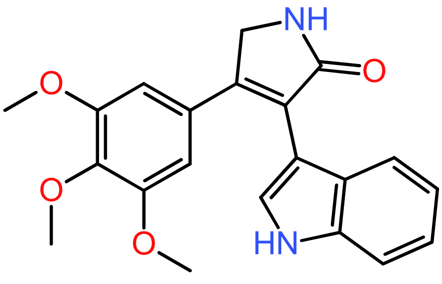

IC50(nM):2100
fringNplaN4B:0
fsp3Csp2N5B:0
N_H_2B:2
fsp2Osp2C5B:4
da_lipo_5B: 11
fringNC6B:1
fOringC6B:2

19 459668

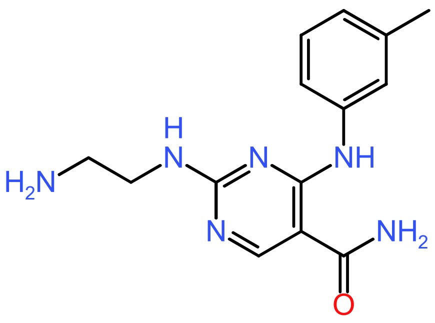

IC50(nM): 1258.9
fringNplaN4B: 1
fsp3Csp2N5B: 0
N_H_2B: 5
fsp2Osp2C5B: 2
da_lipo_5B: 7
fringNC6B: 2
fOringC6B: 2

20 475975

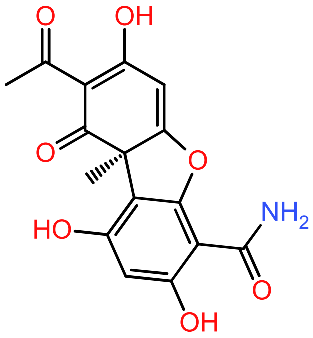

IC50(nM): 2400
fringNplaN4B: 0
fsp3Csp2N5B: 0
N_H_2B: 1
fsp2Osp2C5B: 0
da_lipo_5B: 8
fringNC6B: 0
fOringC6B: 0

21 489011

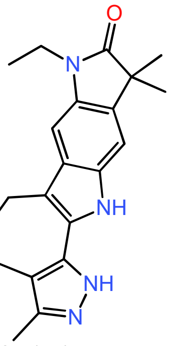

IC50(nM): 138.3333333
fringNplaN4B: 0
fsp3Csp2N5B: 0
N_H_2B: 4
fsp2Osp2C5B: 2
da_lipo_5B: 13
fringNC6B: 0
fOringC6B: 1

22 520121

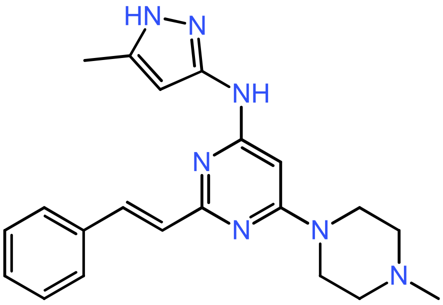

IC50(nM): 305
fringNplaN4B: 0
fsp3Csp2N5B: 1
N_H_2B: 5
fsp2Osp2C5B: 0
da_lipo_5B: 5
fringNC6B: 2
fOringC6B: 0

23 622633

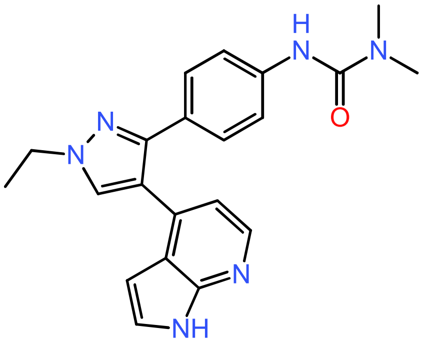

IC50(nM): 26
fringNplaN4B: 0
fsp3Csp2N5B: 0
N_H_2B: 5
fsp2Osp2C5B: 2
da_lipo_5B: 10
fringNC6B: 0
fOringC6B: 1

24 627855

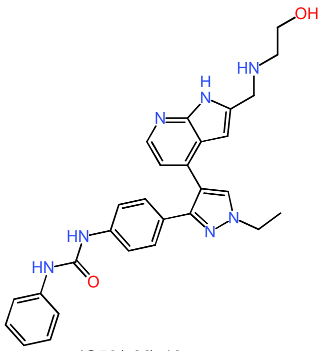

IC50(nM): 46
fringNplaN4B: 0
fsp3Csp2N5B: 0
N_H_2B: 6
fsp2Osp2C5B: 4
da_lipo_5B: 15
fringNC6B: 0
fOringC6B: 3

25 628348

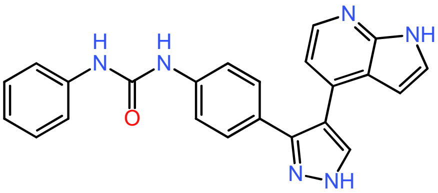

IC50(nM): 32
fringNplaN4B: 0
fsp3Csp2N5B: 0
N_H_2B: 6
fsp2Osp2C5B: 4
da_lipo_5B: 15
fringNC6B: 0
fOringC6B: 2

26 628349

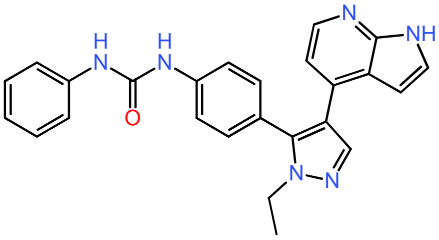

IC50(nM): 25
fringNplaN4B: 0
fsp3Csp2N5B: 0
N_H_2B: 6
fsp2Osp2C5B: 4
da_lipo_5B: 15
fringNC6B: 0
fOringC6B: 2

27 628398

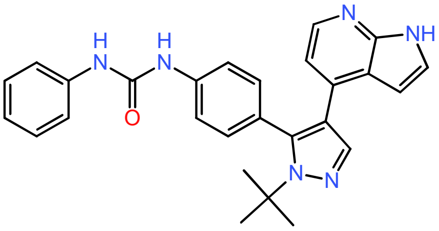

IC50(nM): 40
fringNplaN4B: 0
fsp3Csp2N5B: 0
N_H_2B: 5
fsp2Osp2C5B: 4
da_lipo_5B: 15
fringNC6B: 0
fOringC6B: 2

28 628399

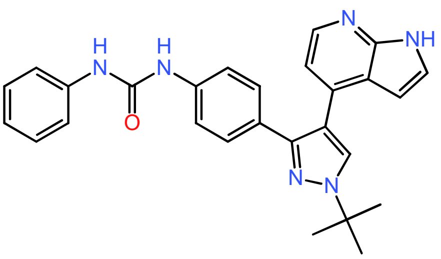

IC50(nM): 50
fringNplaN4B: 0
fsp3Csp2N5B: 0
N_H_2B: 5
fsp2Osp2C5B: 4
da_lipo_5B: 15
fringNC6B: 0
fOringC6B: 2

29 628400

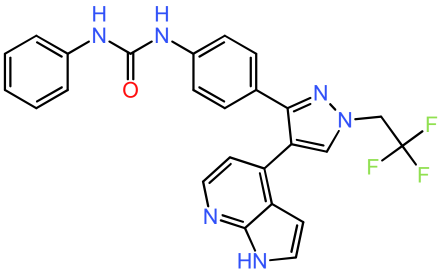

IC50(nM): 16
fringNplaN4B: 0
fsp3Csp2N5B: 0
N_H_2B: 5
fsp2Osp2C5B: 4
da_lipo_5B: 15
fringNC6B: 0
fOringC6B: 2

30 628510

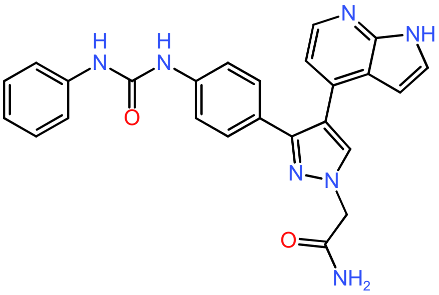

IC50(nM): 64
fringNplaN4B: 0
fsp3Csp2N5B: 0
N_H_2B: 6
fsp2Osp2C5B: 6
da_lipo_5B: 15
fringNC6B: 0
fOringC6B: 3

31 628511

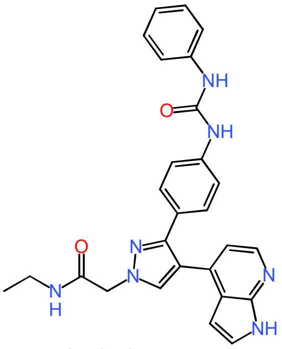

IC50(nM): 64
fringNplaN4B: 0
fsp3Csp2N5B: 0
N_H_2B: 6
fsp2Osp2C5B: 6
da_lipo_5B: 16
fringNC6B: 0
fOringC6B: 3

32 628679

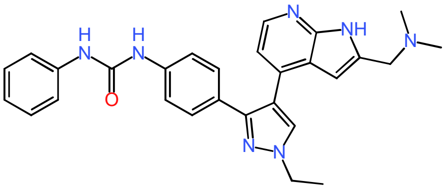

IC50(nM): 58
fringNplaN4B: 0
fsp3Csp2N5B: 0
N_H_2B: 6
fsp2Osp2C5B: 4
da_lipo_5B: 15
fringNC6B: 0
fOringC6B: 2

33 700097

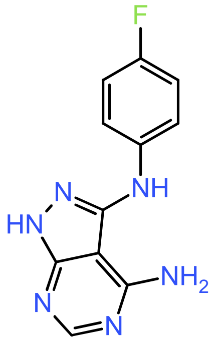

IC50(nM): 660.25
fringNplaN4B: 0
fsp3Csp2N5B: 0
N_H_2B: 6
fsp2Osp2C5B: 0
da_lipo_5B: 7
fringNC6B: 1
fOringC6B: 0

34 702437

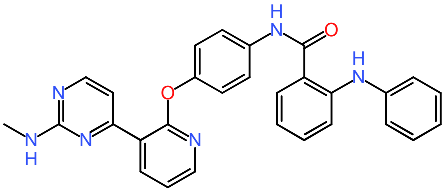

IC50(nM): 12
fringNplaN4B: 0
fsp3Csp2N5B: 0
N_H_2B: 5
fsp2Osp2C5B: 4
da_lipo_5B: 17
fringNC6B: 1
fOringC6B: 2

35 1037720

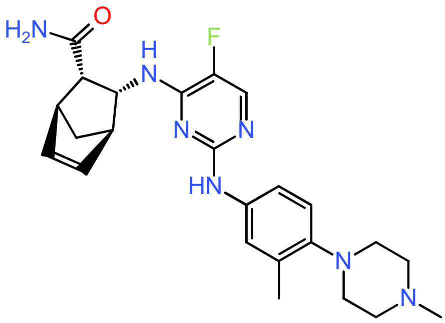

IC50(nM): 54
fringNplaN4B: 0
fsp3Csp2N5B: 1
N_H_2B: 6
fsp2Osp2C5B: 2
da_lipo_5B: 13
fringNC6B: 1
fOringC6B: 1

36 1062266

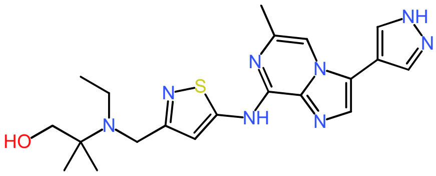

IC50(nM): 19
fringNplaN4B: 0
fsp3Csp2N5B: 1
N_H_2B: 6
fsp2Osp2C5B: 0
da_lipo_5B: 6
fringNC6B: 0
fOringC6B: 1

37 1062267

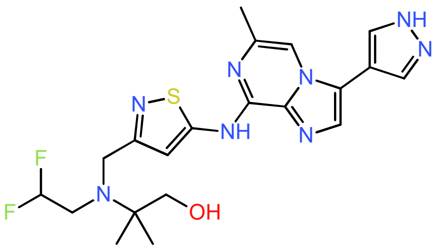

IC50(nM):159.5
fringNplaN4B:0
fsp3Csp2N5B:1
N_H_2B:6
fsp2Osp2C5B:0
da_lipo_5B:6
fringNC6B:0
fOringC6B:1

38 1062268

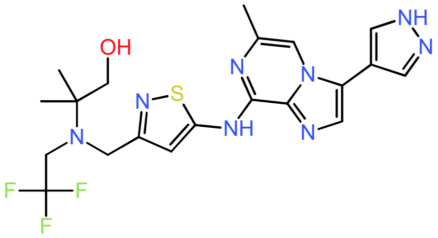

IC50(nM):105.5
fringNplaN4B:0
fsp3Csp2N5B:1
N_H_2B:6
fsp2Osp2C5B:0
da_lipo_5B:6
fringNC6B:0
fOringC6B:1

39 1062269

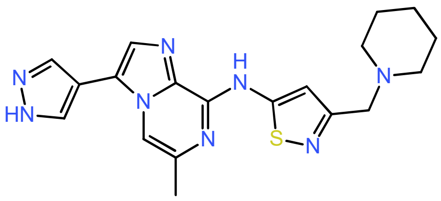

IC50(nM):27.1665
fringNplaN4B:0
fsp3Csp2N5B:1
N_H_2B:6
fsp2Osp2C5B:0
da_lipo_5B:3
fringNC6B:0
fOringC6B:0

40 1062270

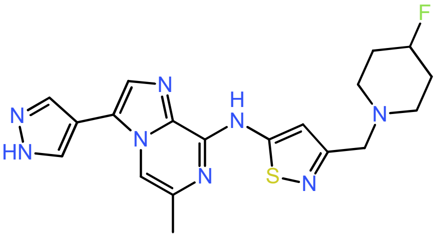

IC50(nM):49.5
fringNplaN4B:0
fsp3Csp2N5B:1
N_H_2B:6
fsp2Osp2C5B:0
da_lipo_5B:3
fringNC6B:0
fOringC6B:0

41 1062271

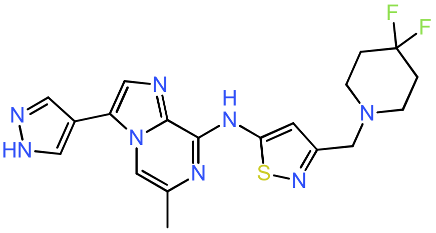

IC50(nM):43.5
fringNplaN4B:0
fsp3Csp2N5B:1
N_H_2B:6
fsp2Osp2C5B:0
da_lipo_5B:3
fringNC6B:0
fOringC6B:0

42 1062272

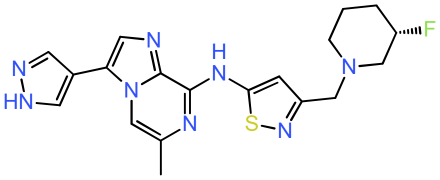

IC50(nM):103
fringNplaN4B:0
fsp3Csp2N5B:1
N_H_2B:6
fsp2Osp2C5B:0
da_lipo_5B:3
fringNC6B:0
fOringC6B:0

43 1062273

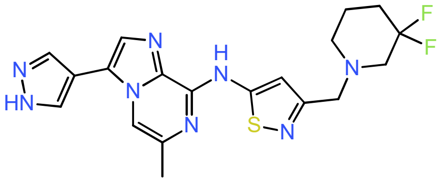

IC50(nM):46
fringNplaN4B:0
fsp3Csp2N5B:1
N_H_2B:6
fsp2Osp2C5B:0
da_lipo_5B:3
fringNC6B:0
fOringC6B:0

44 1062274

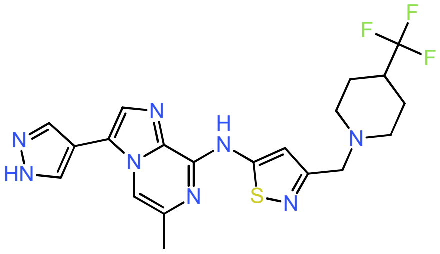

IC50(nM):115.5
fringNplaN4B:0
fsp3Csp2N5B:1
N_H_2B:6
fsp2Osp2C5B:0
da_lipo_5B:3
fringNC6B:0
fOringC6B:0

45 1062275

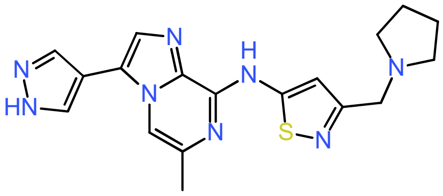

IC50(nM):50.5
fringNplaN4B:0
fsp3Csp2N5B:1
N_H_2B:6
fsp2Osp2C5B:0
da_lipo_5B:3
fringNC6B:0
fOringC6B:0

46 1062276

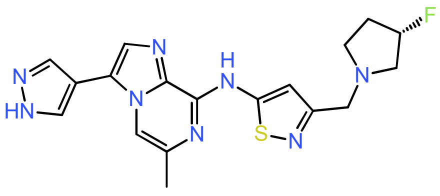

IC50(nM): 50.25
fringNplaN4B: 0
fsp3Csp2N5B: 1
N_H_2B: 6
fsp2Osp2C5B: 0
da_lipo_5B: 3
fringNC6B: 0
fOringC6B: 0

47 1062278

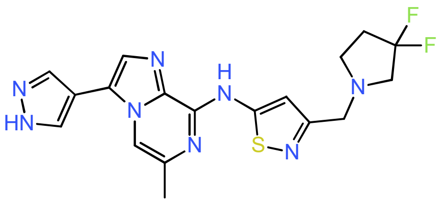

IC50(nM): 52
fringNplaN4B: 0
fsp3Csp2N5B: 1
N_H_2B: 6
fsp2Osp2C5B: 0
da_lipo_5B: 3
fringNC6B: 0
fOringC6B: 0

48 1062279

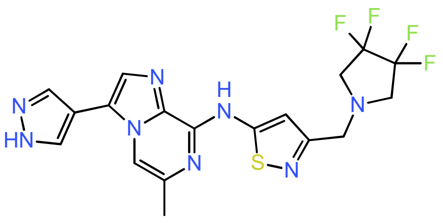

IC50(nM): 31.5
fringNplaN4B: 0
fsp3Csp2N5B: 1
N_H_2B: 6
fsp2Osp2C5B: 0
da_lipo_5B: 3
fringNC6B: 0
fOringC6B: 0

49 1062280

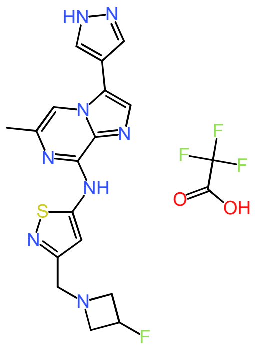

IC50(nM): 102
fringNplaN4B: 0
fsp3Csp2N5B: 1
N_H_2B: 6
fsp2Osp2C5B: 0
da_lipo_5B: 7
fringNC6B: 0
fOringC6B: 0

50 1062281

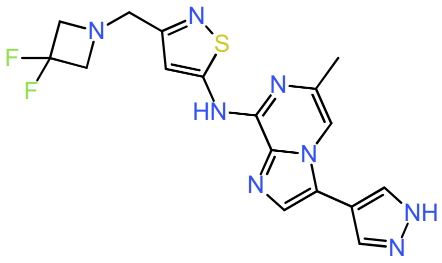

IC50(nM): 44.5
fringNplaN4B: 0
fsp3Csp2N5B: 1
N_H_2B: 6
fsp2Osp2C5B: 0
da_lipo_5B: 7
fringNC6B: 0
fOringC6B: 0

51 1062282

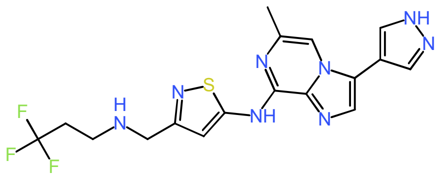

IC50(nM): 38
fringNplaN4B: 0
fsp3Csp2N5B: 1
N_H_2B: 6
fsp2Osp2C5B: 0
da_lipo_5B: 8
fringNC6B: 1
fOringC6B: 0

52 1062283

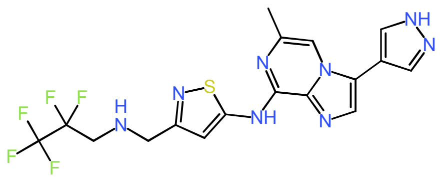

IC50(nM): 32
fringNplaN4B: 0
fsp3Csp2N5B: 1
N_H_2B: 6
fsp2Osp2C5B: 0
da_lipo_5B: 10
fringNC6B: 1
fOringC6B: 0

53 1172566

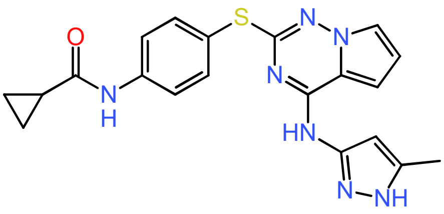

IC50(nM): 225.5
fringNplaN4B: 0
fsp3Csp2N5B: 0
N_H_2B: 5
fsp2Osp2C5B: 2
da_lipo_5B: 13
fringNC6B: 1
fOringC6B: 1

54 1172572

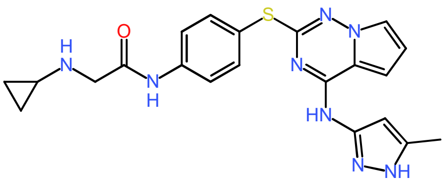

IC50(nM): 164.5
fringNplaN4B: 0
fsp3Csp2N5B: 0
N_H_2B: 6
fsp2Osp2C5B: 2
da_lipo_5B: 12
fringNC6B: 1
fOringC6B: 1

55 1172573

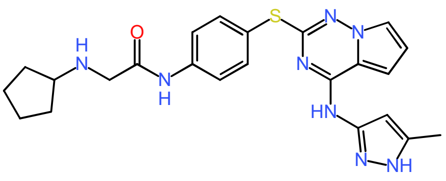

IC50(nM):348.5
fringNplaN4B:0
fsp3Csp2N5B:0
N_H_2B:6
fsp2Osp2C5B:2
da_lipo_5B:14
fringNC6B:1
fOringC6B:3

56 1172575

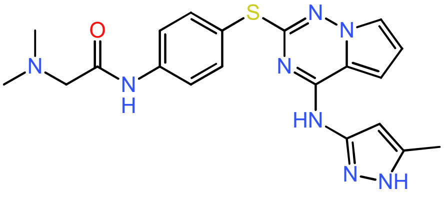

IC50(nM):150.5
fringNplaN4B:0
fsp3Csp2N5B:0
N_H_2B:6
fsp2Osp2C5B:2
da_lipo_5B:10
fringNC6B:1
fOringC6B:1

57 1172576

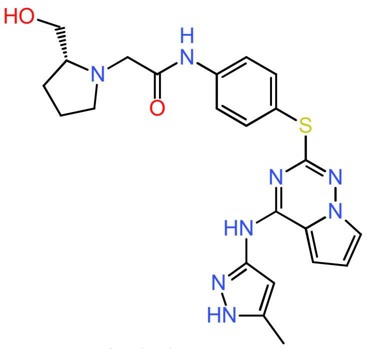

IC50(nM):75
fringNplaN4B:0
fsp3Csp2N5B:0
N_H_2B:6
fsp2Osp2C5B:2
da_lipo_5B:12
fringNC6B:0
fOringC6B:1

58 1172722

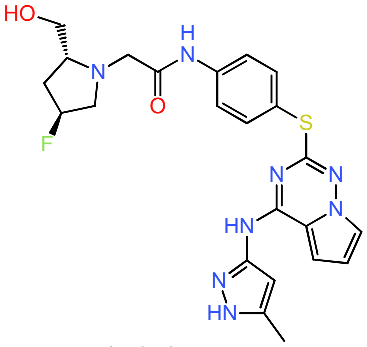

IC50(nM):50
fringNplaN4B:0
fsp3Csp2N5B:0
N_H_2B:6
fsp2Osp2C5B:2
da_lipo_5B:13
fringNC6B:0
fOringC6B:1

59 1172723

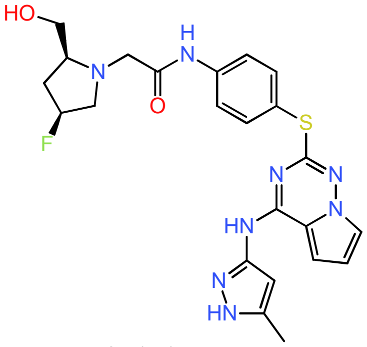

IC50(nM):16
fringNplaN4B:0
fsp3Csp2N5B:0
N_H_2B:6
fsp2Osp2C5B:2
da_lipo_5B:13
fringNC6B:0
fOringC6B:1

60 1172724

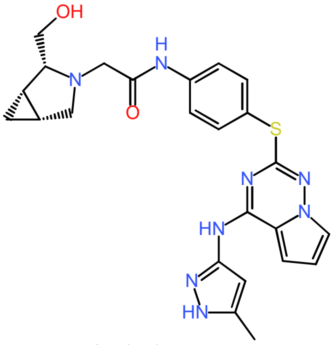

IC50(nM):97
fringNplaN4B:0
fsp3Csp2N5B:0
N_H_2B:6
fsp2Osp2C5B:2
da_lipo_5B:13
fringNC6B:0
fOringC6B:1

61 1172731

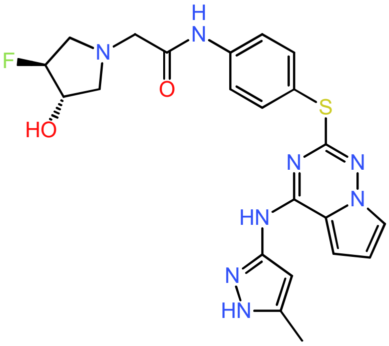

IC50(nM):24
fringNplaN4B:0
fsp3Csp2N5B:0
N_H_2B:6
fsp2Osp2C5B:2
da_lipo_5B:12
fringNC6B:0
fOringC6B:1

62 1178804

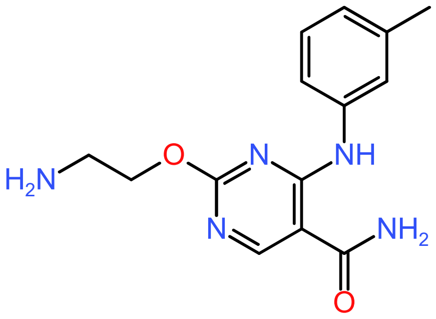

IC50(nM):1995.3
fringNplaN4B:1
fsp3Csp2N5B:0
N_H_2B:4
fsp2Osp2C5B:2
da_lipo_5B:7
fringNC6B:2
fOringC6B:2

63 1178805

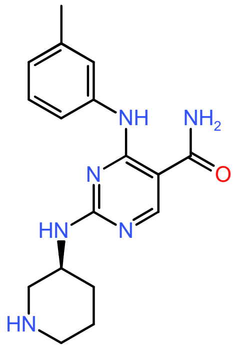

IC50(nM):1780.205
fringNplaN4B:1
fsp3Csp2N5B:0
N_H_2B:5
fsp2Osp2C5B:2
da_lipo_5B:9
fringNC6B:2
fOringC6B:2

64 1179108

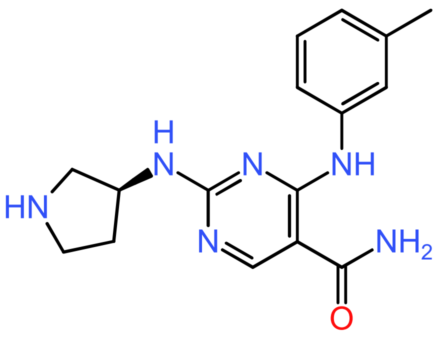

IC50(nM):794.33
fringNplaN4B:1
fsp3Csp2N5B:0
N_H_2B:5
fsp2Osp2C5B:2
da_lipo_5B:8
fringNC6B:2
fOringC6B:2

65 1179115

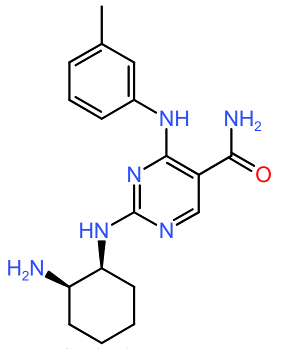

IC50(nM):1995.3
fringNplaN4B:1
fsp3Csp2N5B:0
N_H_2B:5
fsp2Osp2C5B:2
da_lipo_5B:11
fringNC6B:3
fOringC6B:2

66 1179117

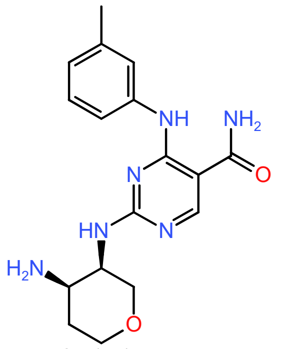

IC50(nM):3981.1
fringNplaN4B:1
fsp3Csp2N5B:0
N_H_2B:5
fsp2Osp2C5B:2
da_lipo_5B:8
fringNC6B:3
fOringC6B:2

67 1179118

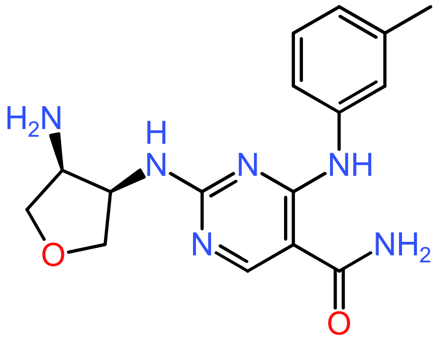

IC50(nM):1995.3
fringNplaN4B:1
fsp3Csp2N5B:0
N_H_2B:5
fsp2Osp2C5B:2
da_lipo_5B:7
fringNC6B:2
fOringC6B:2

68 1179120

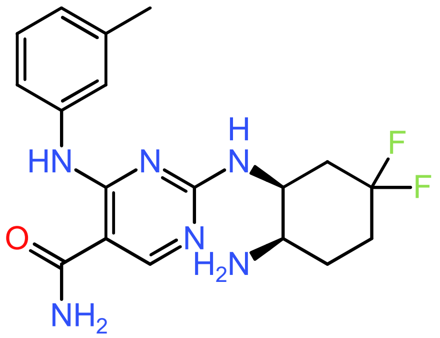

IC50(nM):1258.9
fringNplaN4B:1
fsp3Csp2N5B:0
N_H_2B:5
fsp2Osp2C5B:2
da_lipo_5B:13
fringNC6B:3
fOringC6B:2

69 1285567

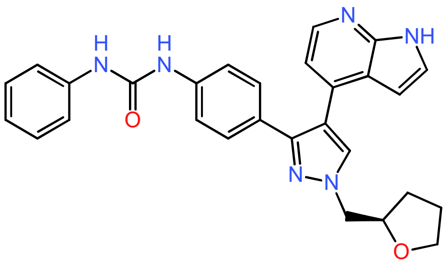

IC50(nM):97
fringNplaN4B:0
fsp3Csp2N5B:0
N_H_2B:5
fsp2Osp2C5B:4
da_lipo_5B:15
fringNC6B:0
fOringC6B:3

70 1288407

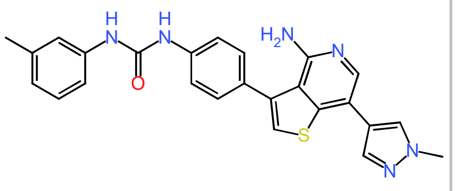

IC50(nM):24.33166667
fringNplaN4B:0
fsp3Csp2N5B:0
N_H_2B:6
fsp2Osp2C5B:4
da_lipo_5B:18
fringNC6B:2
fOringC6B:2

71 1290393

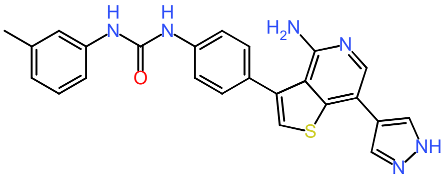

IC50(nM):15.81333333
fringNplaN4B:0
fsp3Csp2N5B:0
N_H_2B:6
fsp2Osp2C5B:4
da_lipo_5B:18
fringNC6B:2
fOringC6B:2

72 1291608

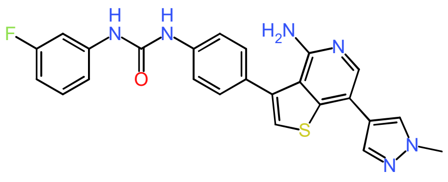

IC50(nM):7.5
fringNplaN4B:0
fsp3Csp2N5B:0
N_H_2B:6
fsp2Osp2C5B:4
da_lipo_5B:18
fringNC6B:2
fOringC6B:2

73 1301174

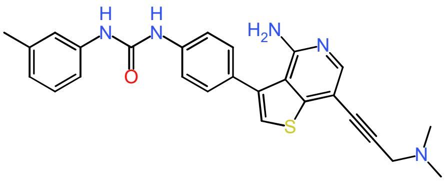

IC50(nM): 76
fringNplaN4B: 0
fsp3Csp2N5B: 1
N_H_2B: 5
fsp2Osp2C5B: 4
da_lipo_5B: 18
fringNC6B: 2
fOringC6B: 2

74 1303757

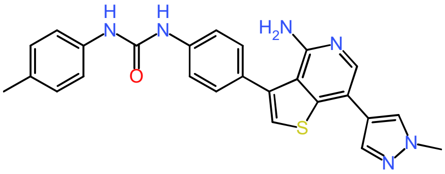

IC50(nM): 57
fringNplaN4B: 0
fsp3Csp2N5B: 0
N_H_2B: 6
fsp2Osp2C5B: 4
da_lipo_5B: 18
fringNC6B: 2
fOringC6B: 2

75 1307949

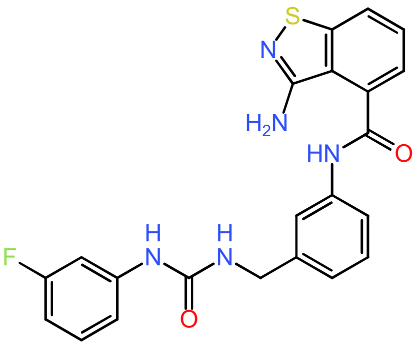

IC50(nM): 50
fringNplaN4B: 0
fsp3Csp2N5B: 0
N_H_2B: 4
fsp2Osp2C5B: 5
da_lipo_5B: 17
fringNC6B: 1
fOringC6B: 1

76 1315232

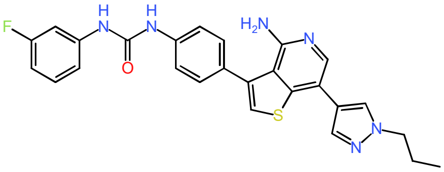

IC50(nM): 17.5
fringNplaN4B: 0
fsp3Csp2N5B: 0
N_H_2B: 6
fsp2Osp2C5B: 4
da_lipo_5B: 18
fringNC6B: 2
fOringC6B: 2

77 1332987

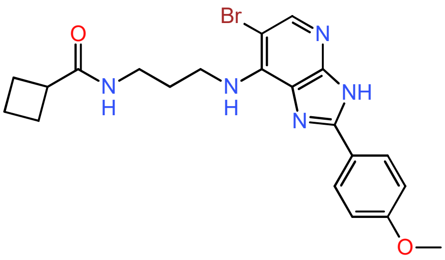

IC50(nM): 38
fringNplaN4B: 0
fsp3Csp2N5B: 1
N_H_2B: 4
fsp2Osp2C5B: 0
da_lipo_5B: 12
fringNC6B: 1
fOringC6B: 0

78 1332990

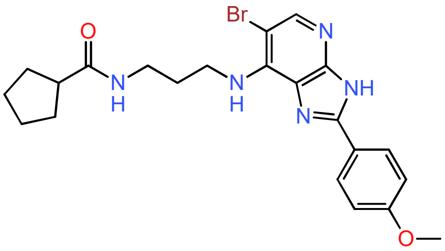

IC50(nM): 93
fringNplaN4B: 0
fsp3Csp2N5B: 1
N_H_2B: 4
fsp2Osp2C5B: 0
da_lipo_5B: 13
fringNC6B: 1
fOringC6B: 0

79 1337477

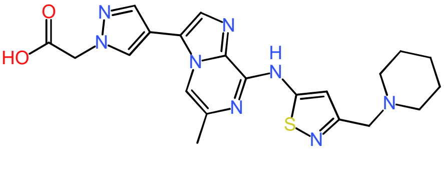

IC50(nM): 363
fringNplaN4B: 0
fsp3Csp2N5B: 1
N_H_2B: 6
fsp2Osp2C5B: 0
da_lipo_5B: 3
fringNC6B: 0
fOringC6B: 1

80 1337478

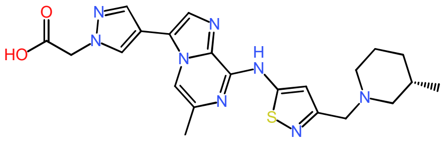

IC50(nM): 146
fringNplaN4B: 0
fsp3Csp2N5B: 1
N_H_2B: 6
fsp2Osp2C5B: 0
da_lipo_5B: 3
fringNC6B: 0
fOringC6B: 1

81 1337479

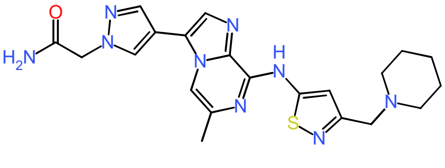

IC50(nM): 514
fringNplaN4B: 0
fsp3Csp2N5B: 1
N_H_2B: 7
fsp2Osp2C5B: 2
da_lipo_5B: 3
fringNC6B: 0
fOringC6B: 1

82 1337480

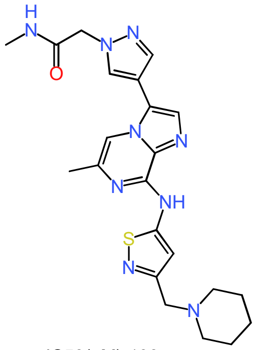

IC50(nM): 193
fringNplaN4B: 0
fsp3Csp2N5B: 1
N_H_2B: 7
fsp2Osp2C5B: 2
da_lipo_5B: 3
fringNC6B: 0
fOringC6B: 1

83 1338596

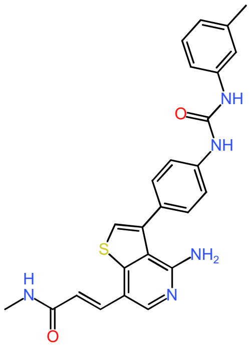

IC50(nM): 83.5
fringNplaN4B: 0
fsp3Csp2N5B: 0
N_H_2B: 5
fsp2Osp2C5B: 6
da_lipo_5B: 19
fringNC6B: 2
fOringC6B: 3

84 1338598

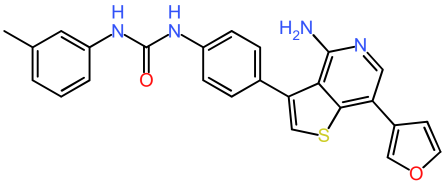

IC50(nM): 200
fringNplaN4B: 0
fsp3Csp2N5B: 0
N_H_2B: 4
fsp2Osp2C5B: 4
da_lipo_5B: 18
fringNC6B: 2
fOringC6B: 5

85 1338599

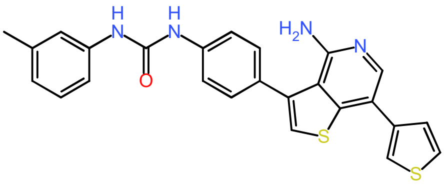

IC50(nM): 239.5
fringNplaN4B: 0
fsp3Csp2N5B: 0
N_H_2B: 4
fsp2Osp2C5B: 4
da_lipo_5B: 18
fringNC6B: 2
fOringC6B: 2

86 1338600

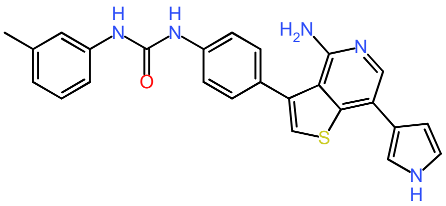

IC50(nM): 228.5
fringNplaN4B: 0
fsp3Csp2N5B: 0
N_H_2B: 5
fsp2Osp2C5B: 4
da_lipo_5B: 19
fringNC6B: 2
fOringC6B: 2

87 1338601

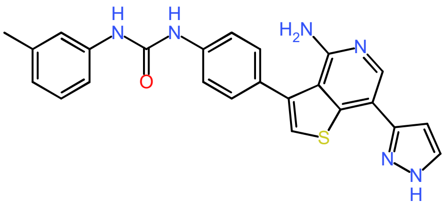

IC50(nM): 113.5
fringNplaN4B: 0
fsp3Csp2N5B: 0
N_H_2B: 6
fsp2Osp2C5B: 4
da_lipo_5B: 18
fringNC6B: 2
fOringC6B: 2

88 1338603

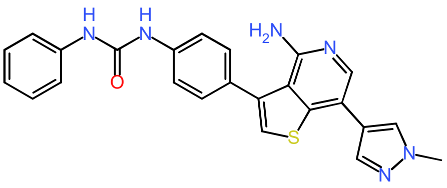

IC50(nM): 7.5
fringNplaN4B: 0
fsp3Csp2N5B: 0
N_H_2B: 6
fsp2Osp2C5B: 4
da_lipo_5B: 17
fringNC6B: 2
fOringC6B: 2

89 1338604

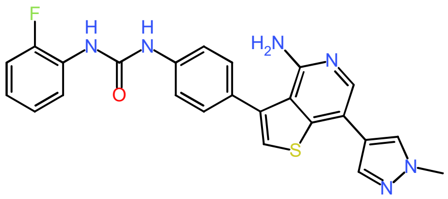

IC50(nM): 24
fringNplaN4B: 0
fsp3Csp2N5B: 0
N_H_2B: 6
fsp2Osp2C5B: 4
da_lipo_5B: 18
fringNC6B: 2
fOringC6B: 2

90 1338605

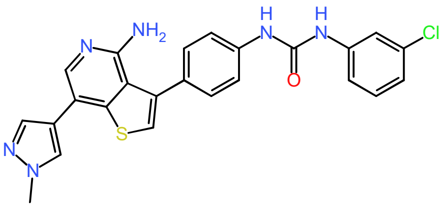

IC50(nM): 44
fringNplaN4B: 0
fsp3Csp2N5B: 0
N_H_2B: 6
fsp2Osp2C5B: 4
da_lipo_5B: 18
fringNC6B: 2
fOringC6B: 2

91 1338606

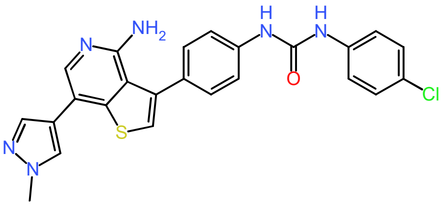

IC50(nM): 37.5
fringNplaN4B: 0
fsp3Csp2N5B: 0
N_H_2B: 6
fsp2Osp2C5B: 4
da_lipo_5B: 18
fringNC6B: 2
fOringC6B: 2

92 1338607

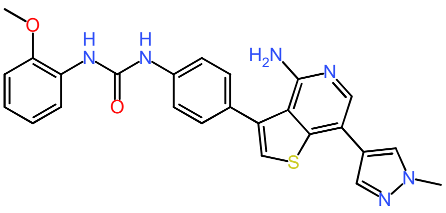

IC50(nM): 76
fringNplaN4B: 0
fsp3Csp2N5B: 0
N_H_2B: 6
fsp2Osp2C5B: 4
da_lipo_5B: 16
fringNC6B: 2
fOringC6B: 1

93 1338608

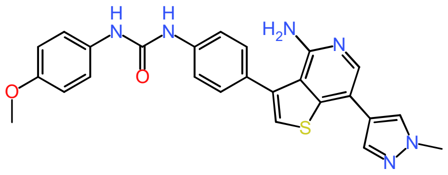

IC50(nM): 85.5
fringNplaN4B: 0
fsp3Csp2N5B: 0
N_H_2B: 6
fsp2Osp2C5B: 4
da_lipo_5B: 16
fringNC6B: 2
fOringC6B: 1

94 1356835

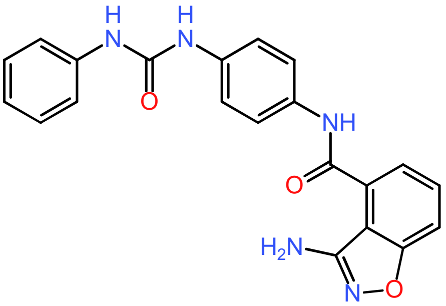

IC50(nM): 127
fringNplaN4B: 0
fsp3Csp2N5B: 0
N_H_2B: 4
fsp2Osp2C5B: 3
da_lipo_5B: 14
fringNC6B: 1
fOringC6B: 1

95 1356836

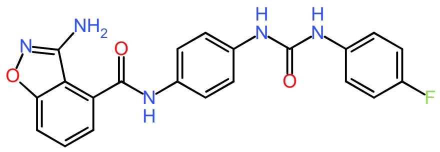

IC50(nM): 480
fringNplaN4B: 0
fsp3Csp2N5B: 0
N_H_2B: 4
fsp2Osp2C5B: 3
da_lipo_5B: 15
fringNC6B: 1
fOringC6B: 1

96 1356837

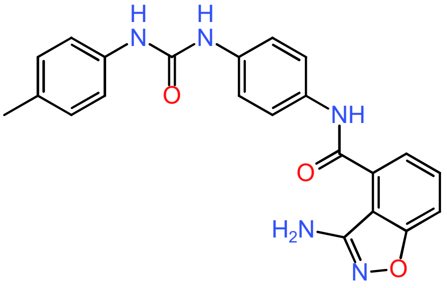

IC50(nM): 82
fringNplaN4B: 0
fsp3Csp2N5B: 0
N_H_2B: 4
fsp2Osp2C5B: 3
da_lipo_5B: 15
fringNC6B: 1
fOringC6B: 1

97 1356838

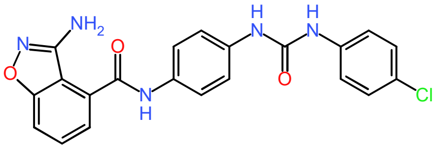

IC50(nM): 140
fringNplaN4B: 0
fsp3Csp2N5B: 0
N_H_2B: 4
fsp2Osp2C5B: 3
da_lipo_5B: 15
fringNC6B: 1
fOringC6B: 1

98 1356839

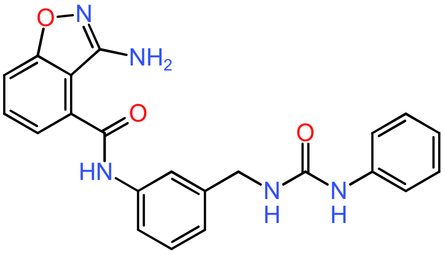

IC50(nM): 18
fringNplaN4B: 0
fsp3Csp2N5B: 0
N_H_2B: 4
fsp2Osp2C5B: 5
da_lipo_5B: 15
fringNC6B: 1
fOringC6B: 1

99 1356840

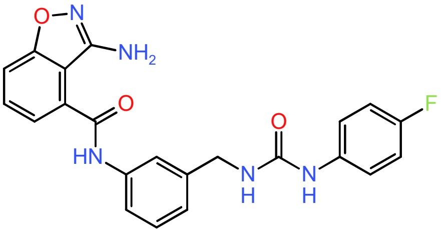

IC50(nM): 41
fringNplaN4B: 0
fsp3Csp2N5B: 0
N_H_2B: 4
fsp2Osp2C5B: 5
da_lipo_5B: 16
fringNC6B: 1
fOringC6B: 1

100 1356843

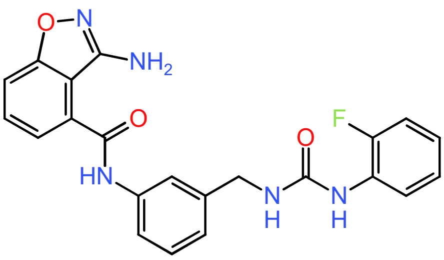

IC50(nM): 55
fringNplaN4B: 0
fsp3Csp2N5B: 0
N_H_2B: 4
fsp2Osp2C5B: 5
da_lipo_5B: 16
fringNC6B: 1
fOringC6B: 1

101 1356844

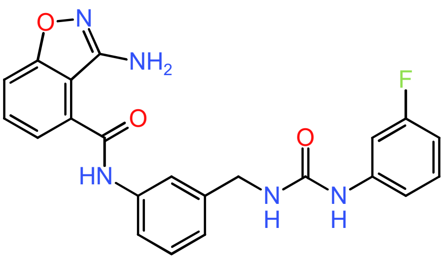

IC50(nM): 59
fringNplaN4B: 0
fsp3Csp2N5B: 0
N_H_2B: 4
fsp2Osp2C5B: 5
da_lipo_5B: 16
fringNC6B: 1
fOringC6B: 1

102 1356845

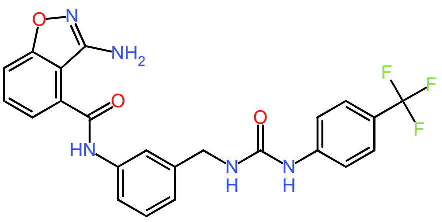

IC50(nM): 12
fringNplaN4B: 0
fsp3Csp2N5B: 0
N_H_2B: 4
fsp2Osp2C5B: 5
da_lipo_5B: 16
fringNC6B: 1
fOringC6B: 1

103 1356846

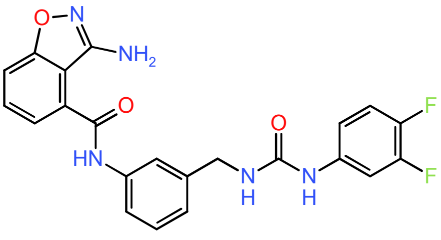

IC50(nM): 7
fringNplaN4B: 0
fsp3Csp2N5B: 0
N_H_2B: 4
fsp2Osp2C5B: 5
da_lipo_5B: 17
fringNC6B: 1
fOringC6B: 1

104 1356847

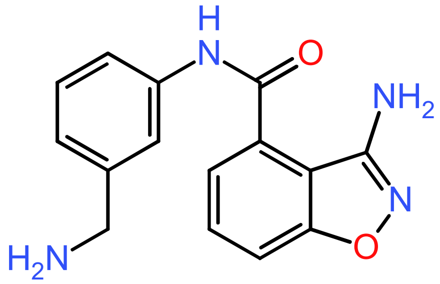

IC50(nM): 525
fringNplaN4B: 0
fsp3Csp2N5B: 0
N_H_2B: 3
fsp2Osp2C5B: 3
da_lipo_5B: 10
fringNC6B: 1
fOringC6B: 1

105 1356849

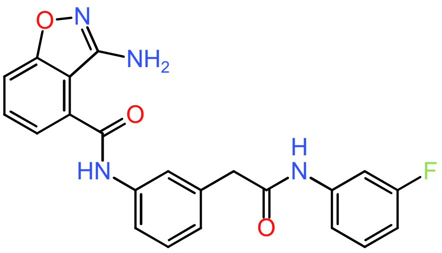

IC50(nM): 450
fringNplaN4B: 0
fsp3Csp2N5B: 0
N_H_2B: 3
fsp2Osp2C5B: 4
da_lipo_5B: 17
fringNC6B: 1
fOringC6B: 1

106 1356851

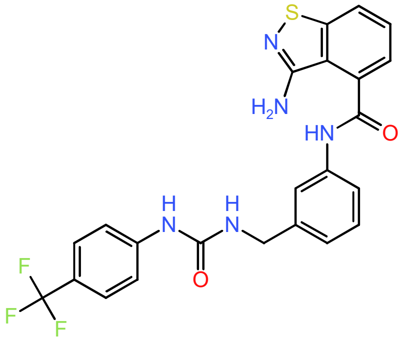

IC50(nM): 12
fringNplaN4B: 0
fsp3Csp2N5B: 0
N_H_2B: 4
fsp2Osp2C5B: 5
da_lipo_5B: 17
fringNC6B: 1
fOringC6B: 1

107 1356852

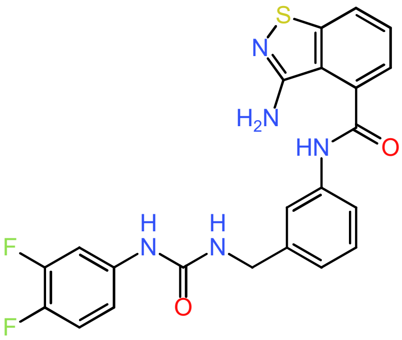

IC50(nM): 36
fringNplaN4B: 0
fsp3Csp2N5B: 0
N_H_2B: 4
fsp2Osp2C5B: 5
da_lipo_5B: 18
fringNC6B: 1
fOringC6B: 1

108 1356853

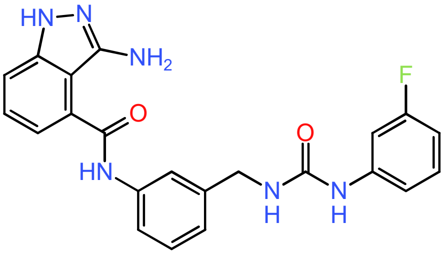

IC50(nM): 18
fringNplaN4B: 0
fsp3Csp2N5B: 0
N_H_2B: 6
fsp2Osp2C5B: 5
da_lipo_5B: 16
fringNC6B: 1
fOringC6B: 1

109 1356854

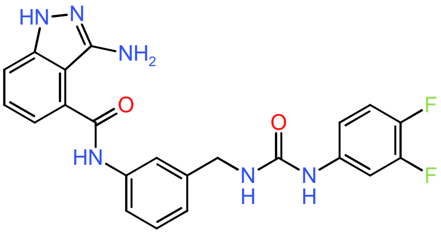

IC50(nM):28
fringNplaN4B:0
fsp3Csp2N5B:0
N_H_2B:6
fsp2Osp2C5B:5
da_lipo_5B:17
fringNC6B:1
fOringC6B:1

110 1356856

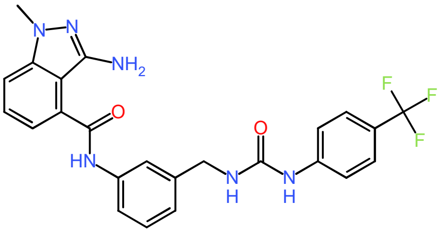

IC50(nM):172
fringNplaN4B:0
fsp3Csp2N5B:0
N_H_2B:5
fsp2Osp2C5B:5
da_lipo_5B:16
fringNC6B:1
fOringC6B:1

111 1356858

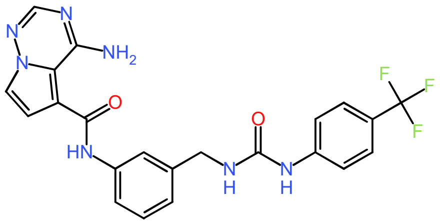

IC50(nM):24
fringNplaN4B:1
fsp3Csp2N5B:0
N_H_2B:7
fsp2Osp2C5B:4
da_lipo_5B:13
fringNC6B:2
fOringC6B:2

112 1356860

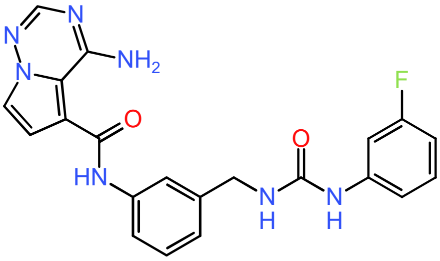

IC50(nM):4
fringNplaN4B:1
fsp3Csp2N5B:0
N_H_2B:7
fsp2Osp2C5B:4
da_lipo_5B:13
fringNC6B:2
fOringC6B:2

113 1356861

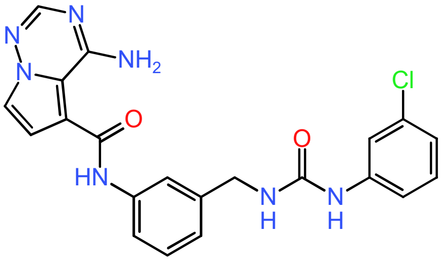

IC50(nM):10
fringNplaN4B:1
fsp3Csp2N5B:0
N_H_2B:7
fsp2Osp2C5B:4
da_lipo_5B:13
fringNC6B:2
fOringC6B:2

114 1356862

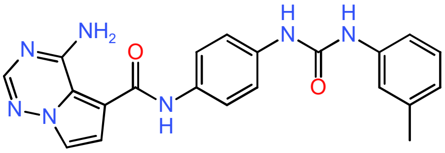

IC50(nM):38
fringNplaN4B:1
fsp3Csp2N5B:0
N_H_2B:7
fsp2Osp2C5B:2
da_lipo_5B:12
fringNC6B:2
fOringC6B:2

115 1356863

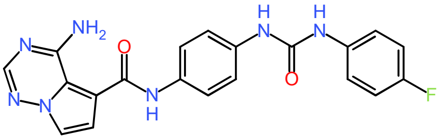

IC50(nM):36
fringNplaN4B:1
fsp3Csp2N5B:0
N_H_2B:7
fsp2Osp2C5B:2
da_lipo_5B:12
fringNC6B:2
fOringC6B:2

116 1356864

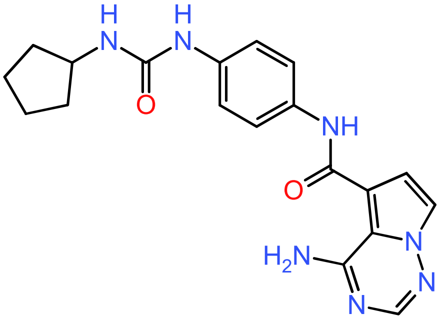

IC50(nM):33
fringNplaN4B:1
fsp3Csp2N5B:0
N_H_2B:7
fsp2Osp2C5B:0
da_lipo_5B:10
fringNC6B:2
fOringC6B:1

117 1361351

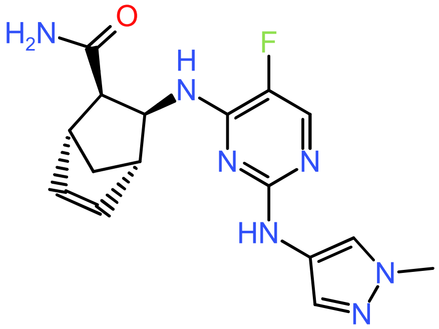

IC50(nM):29
fringNplaN4B:0
fsp3Csp2N5B:1
N_H_2B:6
fsp2Osp2C5B:2
da_lipo_5B:8
fringNC6B:1
fOringC6B:1

118 1361352

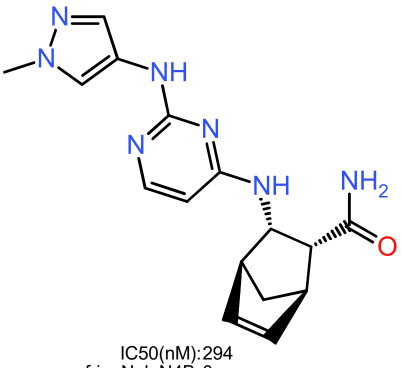

IC50(nM):294
fringNplaN4B:0
fsp3Csp2N5B:1
N_H_2B:6
fsp2Osp2C5B:2
da_lipo_5B:7
fringNC6B:1
fOringC6B:1

119 1361353

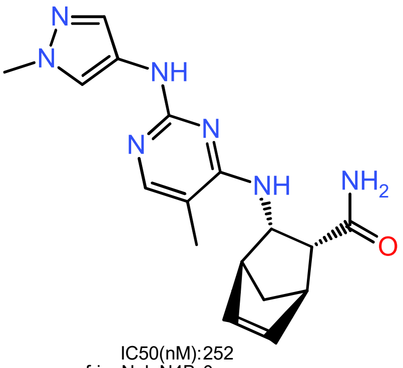

IC50(nM):252
fringNplaN4B:0
fsp3Csp2N5B:0
N_H_2B:6
fsp2Osp2C5B:2
da_lipo_5B:8
fringNC6B:1
fOringC6B:1

120 1361354

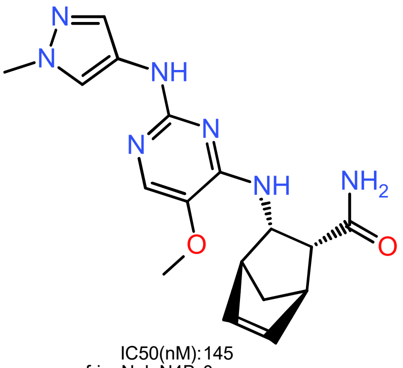

IC50(nM):145
fringNplaN4B:0
fsp3Csp2N5B:0
N_H_2B:6
fsp2Osp2C5B:2
da_lipo_5B:6
fringNC6B:1
fOringC6B:1

121 1361357

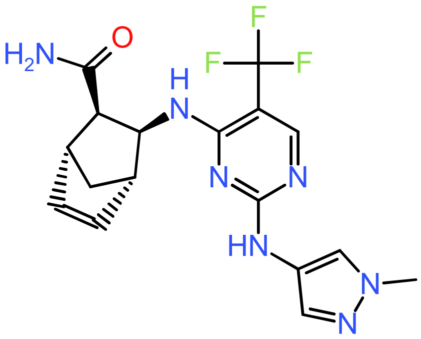

IC50(nM):28
fringNplaN4B:0
fsp3Csp2N5B:0
N_H_2B:6
fsp2Osp2C5B:2
da_lipo_5B:11
fringNC6B:1
fOringC6B:1

122 1361358

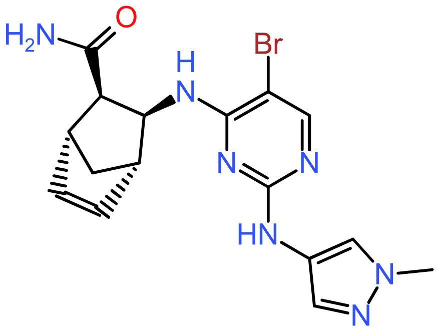

IC50(nM):42
fringNplaN4B:0
fsp3Csp2N5B:1
N_H_2B:6
fsp2Osp2C5B:2
da_lipo_5B:8
fringNC6B:1
fOringC6B:1

123 1361359

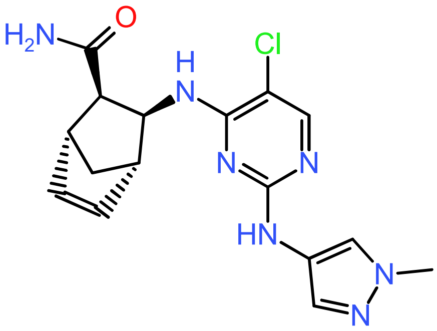

IC50(nM):29
fringNplaN4B:0
fsp3Csp2N5B:1
N_H_2B:6
fsp2Osp2C5B:2
da_lipo_5B:8
fringNC6B:1
fOringC6B:1

124 1361360

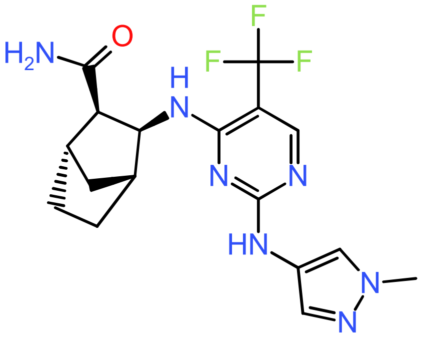

IC50(nM):66
fringNplaN4B:0
fsp3Csp2N5B:0
N_H_2B:6
fsp2Osp2C5B:1
da_lipo_5B:11
fringNC6B:1
fOringC6B:1

125 1361361

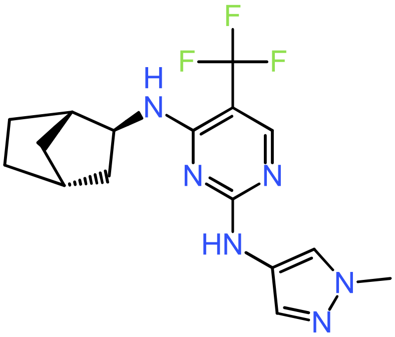

IC50(nM):61
fringNplaN4B:0
fsp3Csp2N5B:0
N_H_2B:5
fsp2Osp2C5B:0
da_lipo_5B:11
fringNC6B:1
fOringC6B:0

126 1361363

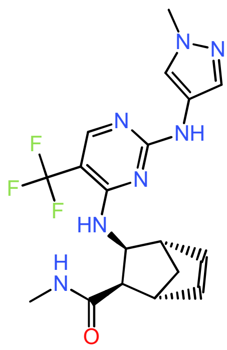

IC50(nM):45
fringNplaN4B:0
fsp3Csp2N5B:0
N_H_2B:6
fsp2Osp2C5B:2
da_lipo_5B:11
fringNC6B:1
fOringC6B:1

127 1361364

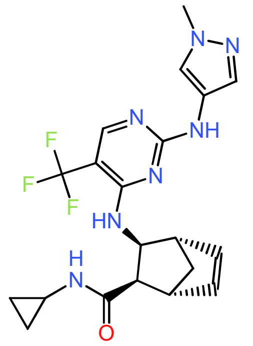

IC50(nM): 51
fringNplaN4B: 0
fsp3Csp2N5B: 0
N_H_2B: 6
fsp2Osp2C5B: 2
da_lipo_5B: 13
fringNC6B: 1
fOringC6B: 1

128 1361365

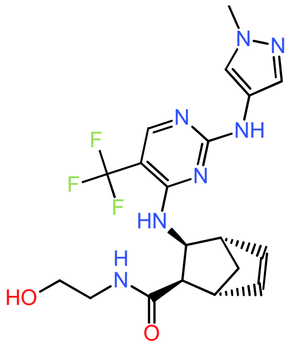

IC50(nM): 99
fringNplaN4B: 0
fsp3Csp2N5B: 0
N_H_2B: 6
fsp2Osp2C5B: 2
da_lipo_5B: 11
fringNC6B: 1
fOringC6B: 1

129 1361368

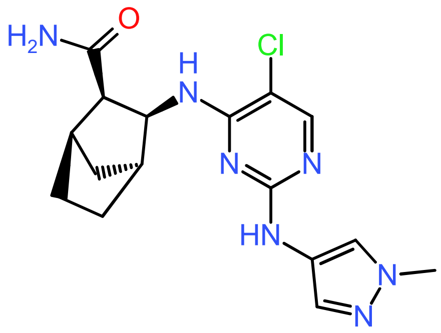

IC50(nM): 31
fringNplaN4B: 0
fsp3Csp2N5B: 1
N_H_2B: 6
fsp2Osp2C5B: 1
da_lipo_5B: 8
fringNC6B: 1
fOringC6B: 1

130 1361369

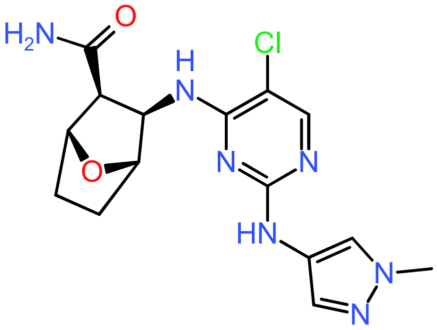

IC50(nM): 118
fringNplaN4B: 0
fsp3Csp2N5B: 1
N_H_2B: 6
fsp2Osp2C5B: 1
da_lipo_5B: 5
fringNC6B: 1
fOringC6B: 2

131 1361370

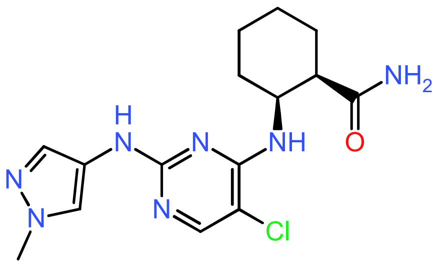

IC50(nM): 135
fringNplaN4B: 0
fsp3Csp2N5B: 1
N_H_2B: 6
fsp2Osp2C5B: 1
da_lipo_5B: 7
fringNC6B: 1
fOringC6B: 1

132 1361371

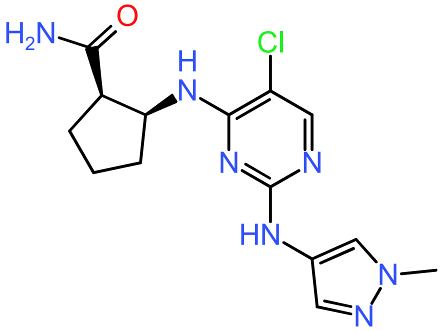

IC50(nM): 32
fringNplaN4B: 0
fsp3Csp2N5B: 1
N_H_2B: 6
fsp2Osp2C5B: 1
da_lipo_5B: 6
fringNC6B: 0
fOringC6B: 1

133 1361372

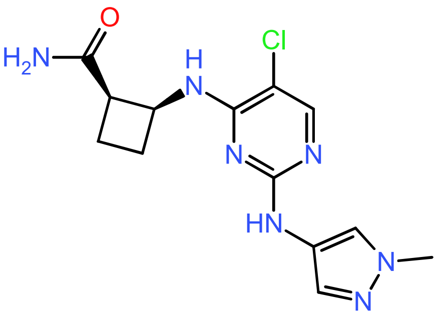

IC50(nM): 44
fringNplaN4B: 0
fsp3Csp2N5B: 1
N_H_2B: 6
fsp2Osp2C5B: 1
da_lipo_5B: 5
fringNC6B: 0
fOringC6B: 1

134 1361374

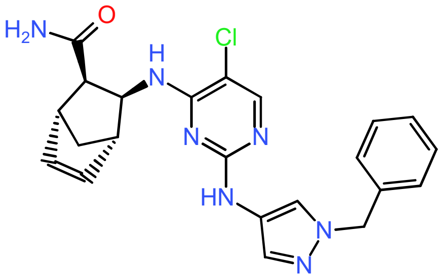

IC50(nM): 12
fringNplaN4B: 0
fsp3Csp2N5B: 1
N_H_2B: 6
fsp2Osp2C5B: 2
da_lipo_5B: 9
fringNC6B: 1
fOringC6B: 1

135 1361428

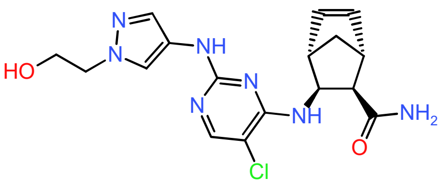

IC50(nM): 19
fringNplaN4B: 0
fsp3Csp2N5B: 1
N_H_2B: 6
fsp2Osp2C5B: 2
da_lipo_5B: 8
fringNC6B: 1
fOringC6B: 1

136 1361429

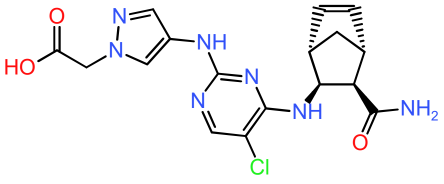

IC50(nM): 32
fringNplaN4B: 0
fsp3Csp2N5B: 1
N_H_2B: 6
fsp2Osp2C5B: 2
da_lipo_5B: 8
fringNC6B: 1
fOringC6B: 1

137 1361431

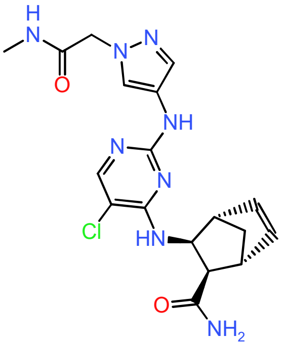

IC50(nM): 26
fringNplaN4B: 0
fsp3Csp2N5B: 1
N_H_2B: 7
fsp2Osp2C5B: 4
da_lipo_5B: 8
fringNC6B: 1
fOringC6B: 1

138 1361432

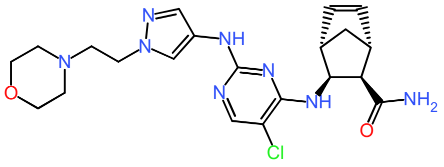

IC50(nM): 27
fringNplaN4B: 0
fsp3Csp2N5B: 1
N_H_2B: 7
fsp2Osp2C5B: 2
da_lipo_5B: 8
fringNC6B: 1
fOringC6B: 1

139 1361433

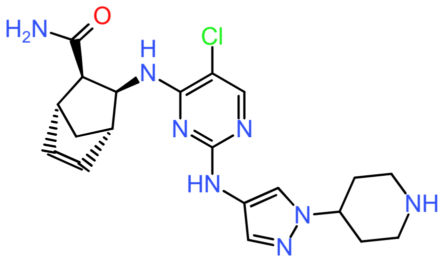

IC50(nM): 12
fringNplaN4B: 0
fsp3Csp2N5B: 1
N_H_2B: 7
fsp2Osp2C5B: 2
da_lipo_5B: 10
fringNC6B: 1
fOringC6B: 1

140 1361434

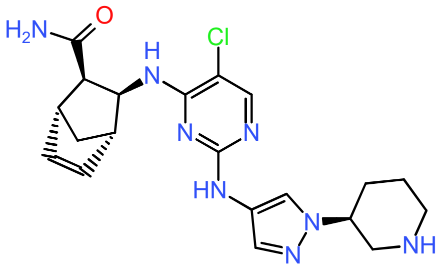

IC50(nM): 12
fringNplaN4B: 0
fsp3Csp2N5B: 1
N_H_2B: 7
fsp2Osp2C5B: 2
da_lipo_5B: 10
fringNC6B: 1
fOringC6B: 1

141 1361435

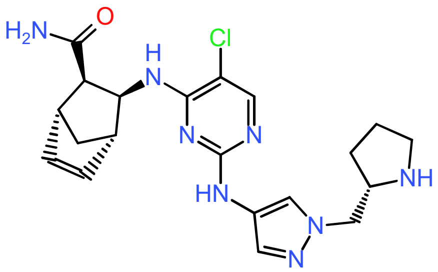

IC50(nM): 15
fringNplaN4B: 0
fsp3Csp2N5B: 1
N_H_2B: 7
fsp2Osp2C5B: 2
da_lipo_5B: 10
fringNC6B: 1
fOringC6B: 1

142 1426822

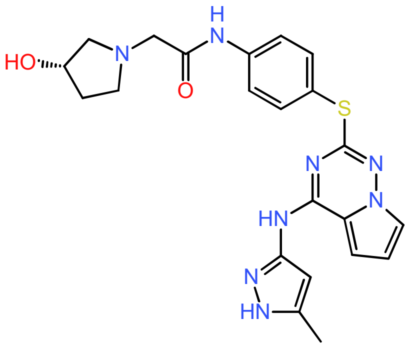

IC50(nM): 136.1
fringNplaN4B: 0
fsp3Csp2N5B: 0
N_H_2B: 6
fsp2Osp2C5B: 2
da_lipo_5B: 11
fringNC6B: 0
fOringC6B: 1

143 1429887

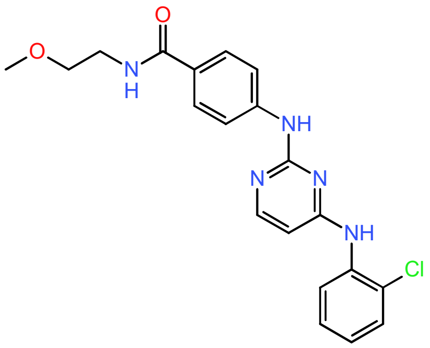

IC50(nM): 561.5
fringNplaN4B: 0
fsp3Csp2N5B: 0
N_H_2B: 4
fsp2Osp2C5B: 1
da_lipo_5B: 12
fringNC6B: 2
fOringC6B: 0

144 1429888

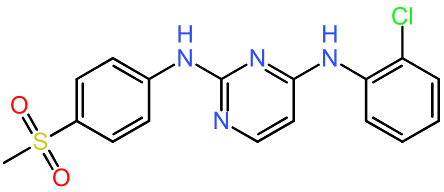

IC50(nM): 5090
fringNplaN4B: 0
fsp3Csp2N5B: 0
N_H_2B: 3
fsp2Osp2C5B: 1
da_lipo_5B: 12
fringNC6B: 2
fOringC6B: 0

145 1429890

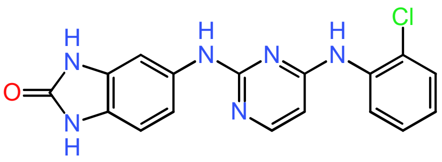

IC50(nM):680.5
fringNplaN4B:0
fsp3Csp2N5B:0
N_H_2B:5
fsp2Osp2C5B:2
da_lipo_5B: 10
fringNC6B:1
fOringC6B:0

146 1429891

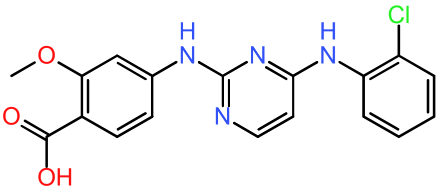

IC50(nM):5755
fringNplaN4B:0
fsp3Csp2N5B:0
N_H_2B:3
fsp2Osp2C5B:0
da_lipo_5B: 11
fringNC6B:2
fOringC6B:0

147 1429892

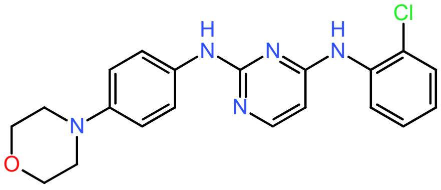

IC50(nM):597
fringNplaN4B:0
fsp3Csp2N5B:0
N_H_2B:4
fsp2Osp2C5B:0
da_lipo_5B: 11
fringNC6B:1
fOringC6B:2

148 1429893

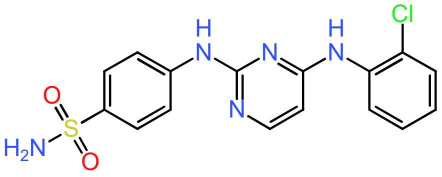

IC50(nM):521.65
fringNplaN4B:0
fsp3Csp2N5B:0
N_H_2B:4
fsp2Osp2C5B:1
da_lipo_5B: 12
fringNC6B:2
fOringC6B:0

149 1429894

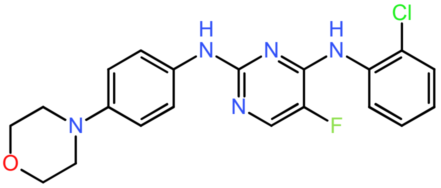

IC50(nM):512.8
fringNplaN4B:0
fsp3Csp2N5B:0
N_H_2B:4
fsp2Osp2C5B:0
da_lipo_5B: 12
fringNC6B:1
fOringC6B:2

150 1429895

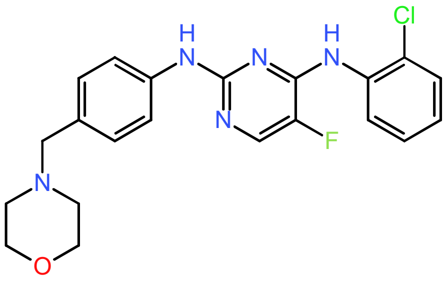

IC50(nM):515
fringNplaN4B:0
fsp3Csp2N5B:0
N_H_2B:4
fsp2Osp2C5B:0
da_lipo_5B: 13
fringNC6B:1
fOringC6B:2

151 1429896

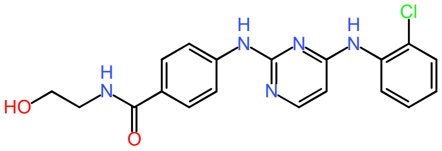

IC50(nM):520.2
fringNplaN4B:0
fsp3Csp2N5B:0
N_H_2B:4
fsp2Osp2C5B:1
da_lipo_5B: 12
fringNC6B:2
fOringC6B:0

152 1429897

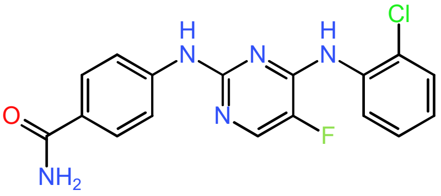

IC50(nM):367.9
fringNplaN4B:0
fsp3Csp2N5B:0
N_H_2B:4
fsp2Osp2C5B:1
da_lipo_5B: 13
fringNC6B:2
fOringC6B:0

153 1429902

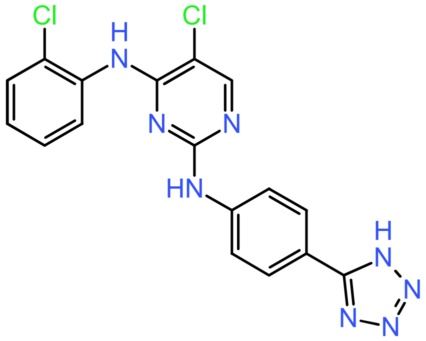

IC50(nM):553
fringNplaN4B:0
fsp3Csp2N5B:0
N_H_2B:5
fsp2Osp2C5B:0
da_lipo_5B: 13
fringNC6B:1
fOringC6B:0

154 1429926

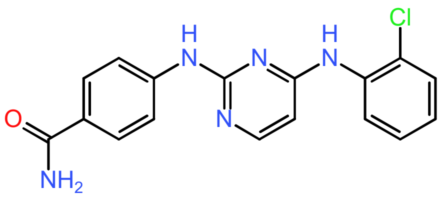

IC50(nM):525.1
fringNplaN4B:0
fsp3Csp2N5B:0
N_H_2B:4
fsp2Osp2C5B:1
da_lipo_5B:12
fringNC6B:2
fOringC6B:0

155 1430075

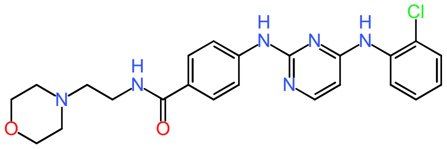

IC50(nM):616
fringNplaN4B:0
fsp3Csp2N5B:0
N_H_2B:5
fsp2Osp2C5B:1
da_lipo_5B:12
fringNC6B:1
fOringC6B:0

156 1430076

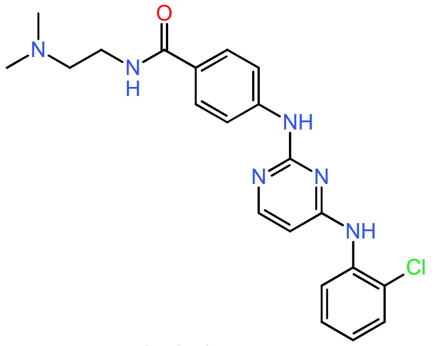

IC50(nM):546.95
fringNplaN4B:0
fsp3Csp2N5B:0
N_H_2B:5
fsp2Osp2C5B:1
da_lipo_5B:12
fringNC6B:2
fOringC6B:0

157 1447148

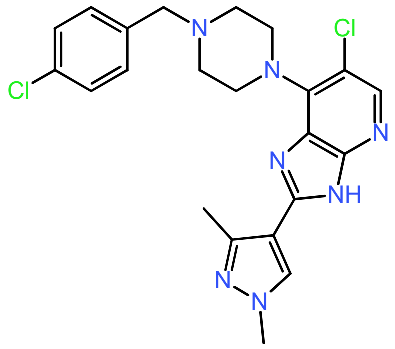

IC50(nM):81.33333333
fringNplaN4B:0
fsp3Csp2N5B:1
N_H_2B:5
fsp2Osp2C5B:0
da_lipo_5B:4
fringNC6B:0
fOringC6B:0

158 1523084

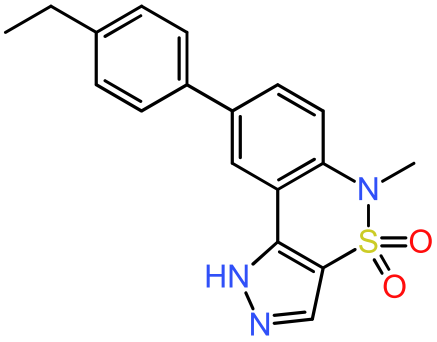

IC50(nM):2593
fringNplaN4B:0
fsp3Csp2N5B:1
N_H_2B:3
fsp2Osp2C5B:2
da_lipo_5B:7
fringNC6B:2
fOringC6B:1

159 1528877

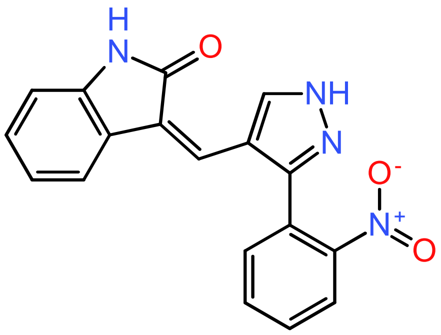

IC50(nM):20.43333333
fringNplaN4B:1
fsp3Csp2N5B:0
N_H_2B:3
fsp2Osp2C5B:4
da_lipo_5B:12
fringNC6B:0
fOringC6B:0

160 1560226

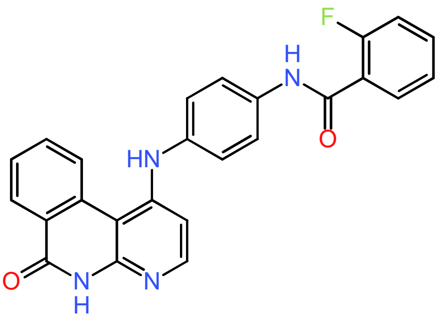

IC50(nM):2
fringNplaN4B:1
fsp3Csp2N5B:0
N_H_2B:4
fsp2Osp2C5B:6
da_lipo_5B:19
fringNC6B:2
fOringC6B:2

161 1560227

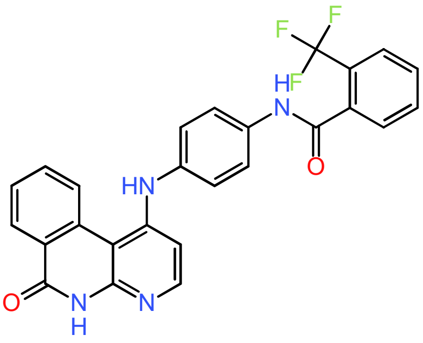

IC50(nM):1
fringNplaN4B:1
fsp3Csp2N5B:0
N_H_2B:4
fsp2Osp2C5B:6
da_lipo_5B:22
fringNC6B:2
fOringC6B:2

162 1560231

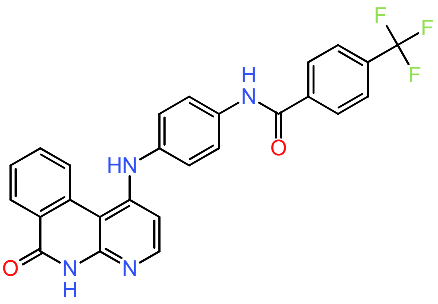

IC50(nM):6
fringNplaN4B:1
fsp3Csp2N5B:0
N_H_2B:4
fsp2Osp2C5B:6
da_lipo_5B:18
fringNC6B:2
fOringC6B:2

163 1560237

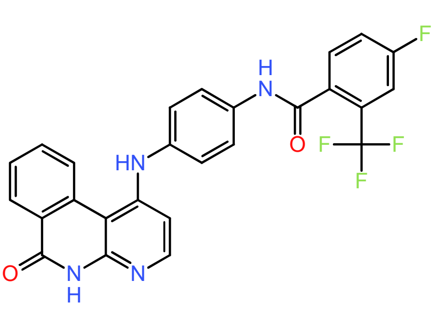

IC50(nM): 26.2
fringNplaN4B: 1
fsp3Csp2N5B: 0
N_H_2B: 4
fsp2Osp2C5B: 6
da_lipo_5B: 22
fringNC6B: 2
fOringC6B: 2

164 1560243

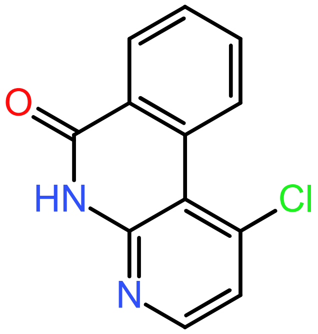

IC50(nM): 1440
fringNplaN4B: 0
fsp3Csp2N5B: 0
N_H_2B: 2
fsp2Osp2C5B: 3
da_lipo_5B: 10
fringNC6B: 0
fOringC6B: 1

165 1560246

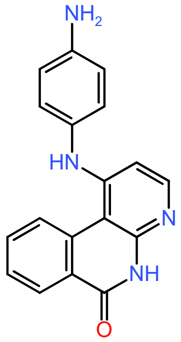

IC50(nM): 174
fringNplaN4B: 1
fsp3Csp2N5B: 0
N_H_2B: 4
fsp2Osp2C5B: 3
da_lipo_5B: 12
fringNC6B: 2
fOringC6B: 1

166 1560248

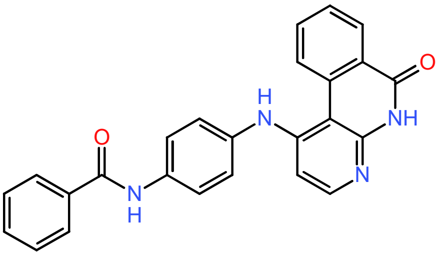

IC50(nM): 104.5
fringNplaN4B: 1
fsp3Csp2N5B: 0
N_H_2B: 4
fsp2Osp2C5B: 6
da_lipo_5B: 18
fringNC6B: 2
fOringC6B: 2

167 1560249

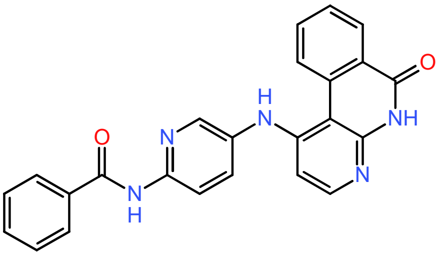

IC50(nM): 13
fringNplaN4B: 0
fsp3Csp2N5B: 0
N_H_2B: 5
fsp2Osp2C5B: 6
da_lipo_5B: 16
fringNC6B: 2
fOringC6B: 2

168 1560250

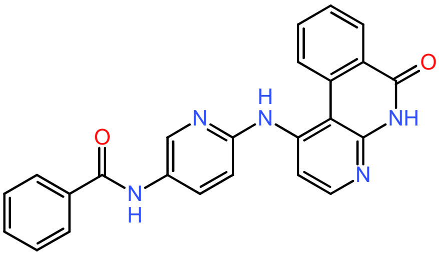

IC50(nM): 79
fringNplaN4B: 0
fsp3Csp2N5B: 0
N_H_2B: 5
fsp2Osp2C5B: 5
da_lipo_5B: 16
fringNC6B: 2
fOringC6B: 2

169 1560251

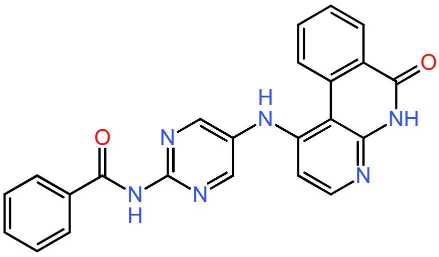

IC50(nM): 21
fringNplaN4B: 0
fsp3Csp2N5B: 0
N_H_2B: 6
fsp2Osp2C5B: 6
da_lipo_5B: 14
fringNC6B: 2
fOringC6B: 2

170 1565680

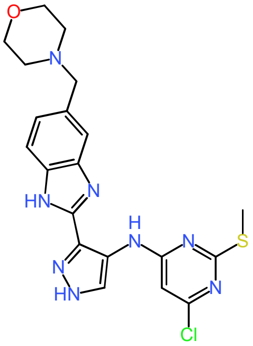

IC50(nM): 143.9
fringNplaN4B: 0
fsp3Csp2N5B: 0
N_H_2B: 5
fsp2Osp2C5B: 0
da_lipo_5B: 8
fringNC6B: 0
fOringC6B: 2

171 1565683

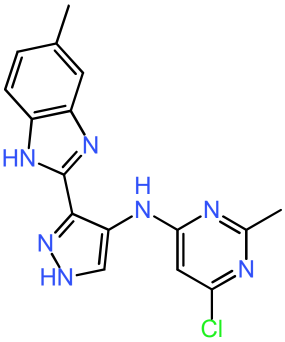

IC50(nM): 138.6
fringNplaN4B: 0
fsp3Csp2N5B: 0
N_H_2B: 4
fsp2Osp2C5B: 0
da_lipo_5B: 8
fringNC6B: 0
fOringC6B: 0

172 1565684

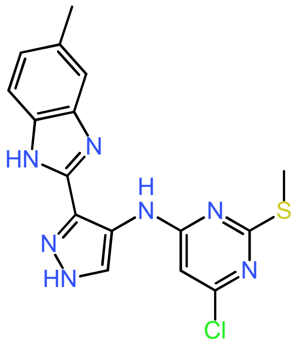

IC50(nM):231.3
fringNplaN4B:0
fsp3Csp2N5B:0
N_H_2B:4
fsp2Osp2C5B:0
da_lipo_5B:9
fringNC6B:0
fOringC6B:0

173 1565686

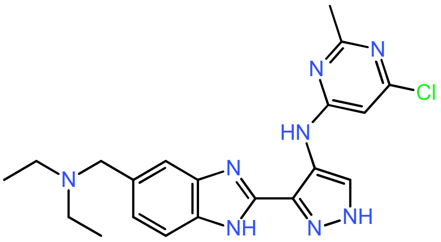

IC50(nM):1201
fringNplaN4B:0
fsp3Csp2N5B:0
N_H_2B:5
fsp2Osp2C5B:0
da_lipo_5B:7
fringNC6B:2
fOringC6B:0

174 1565691

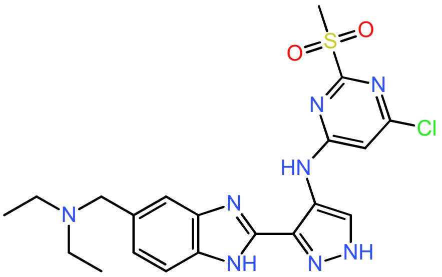

IC50(nM):392.3
fringNplaN4B:0
fsp3Csp2N5B:0
N_H_2B:5
fsp2Osp2C5B:1
da_lipo_5B:7
fringNC6B:2
fOringC6B:1

175 1569417

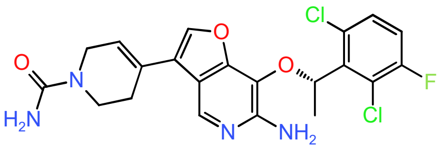

IC50(nM):3670.333333
fringNplaN4B:0
fsp3Csp2N5B:0
N_H_2B:4
fsp2Osp2C5B:1
da_lipo_5B:7
fringNC6B:2
fOringC6B:0

176 1572155

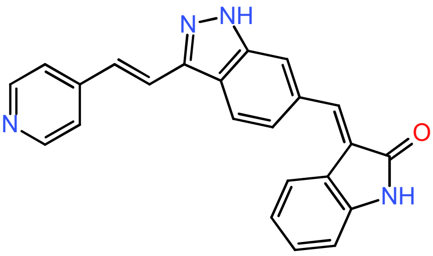

IC50(nM):24
fringNplaN4B:0
fsp3Csp2N5B:0
N_H_2B:4
fsp2Osp2C5B:4
da_lipo_5B:15
fringNC6B:0
fOringC6B:2

177 1572308

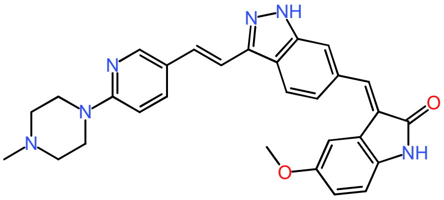

IC50(nM):210
fringNplaN4B:0
fsp3Csp2N5B:0
N_H_2B:6
fsp2Osp2C5B:4
da_lipo_5B:14
fringNC6B:1
fOringC6B:2

178 1573025

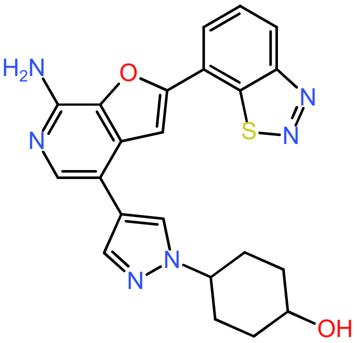

IC50(nM):160
fringNplaN4B:0
fsp3Csp2N5B:0
N_H_2B:4
fsp2Osp2C5B:0
da_lipo_5B:9
fringNC6B:0
fOringC6B:0

179 1573032

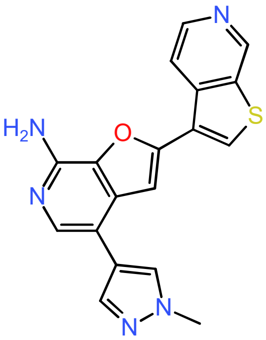

IC50(nM):3000
fringNplaN4B:0
fsp3Csp2N5B:0
N_H_2B:5
fsp2Osp2C5B:0
da_lipo_5B:5
fringNC6B:0
fOringC6B:0

180 1586133

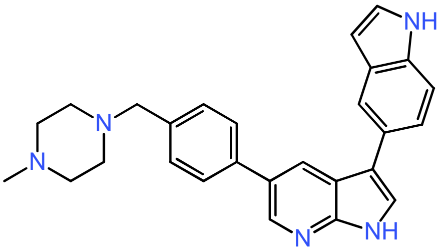

IC50(nM):123
fringNplaN4B:0
fsp3Csp2N5B:0
N_H_2B:5
fsp2Osp2C5B:0
da_lipo_5B:11
fringNC6B:0
fOringC6B:0

181 1587785

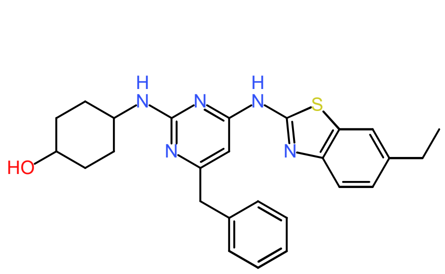

IC50(nM):398.11
fringNplaN4B:0
fsp3Csp2N5B:1
N_H_2B:2
fsp2Osp2C5B:0
da_lipo_5B:13
fringNC6B:3
fOringC6B:1

182 1605253

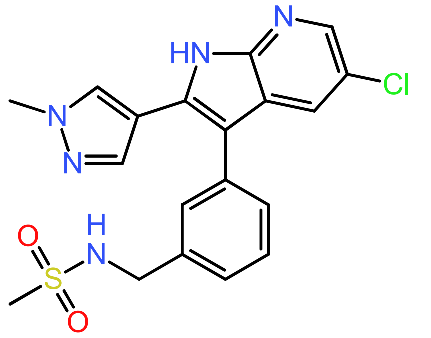

IC50(nM):441
fringNplaN4B:0
fsp3Csp2N5B:0
N_H_2B:5
fsp2Osp2C5B:2
da_lipo_5B:13
fringNC6B:2
fOringC6B:2

183 1605254

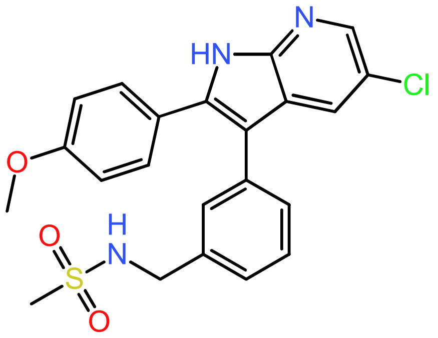

IC50(nM):7095
fringNplaN4B:0
fsp3Csp2N5B:0
N_H_2B:3
fsp2Osp2C5B:2
da_lipo_5B:17
fringNC6B:2
fOringC6B:3

184 1605255

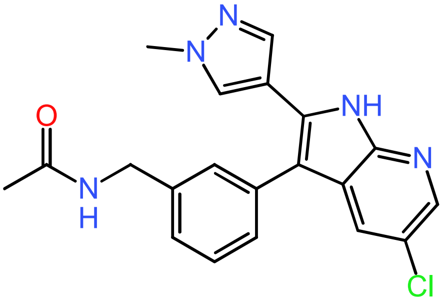

IC50(nM):97
fringNplaN4B:0
fsp3Csp2N5B:0
N_H_2B:5
fsp2Osp2C5B:2
da_lipo_5B:13
fringNC6B:2
fOringC6B:2

185 1605256

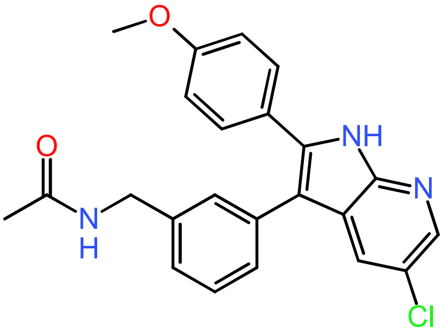

IC50(nM):1087
fringNplaN4B:0
fsp3Csp2N5B:0
N_H_2B:3
fsp2Osp2C5B:2
da_lipo_5B:17
fringNC6B:2
fOringC6B:3

186 1605257

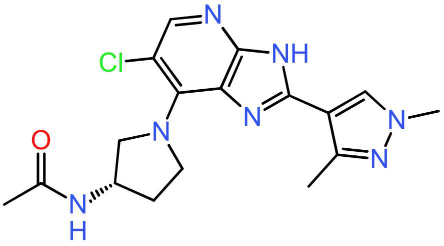

IC50(nM):41
fringNplaN4B:0
fsp3Csp2N5B:1
N_H_2B:5
fsp2Osp2C5B:0
da_lipo_5B:6
fringNC6B:0
fOringC6B:1

187 1605263

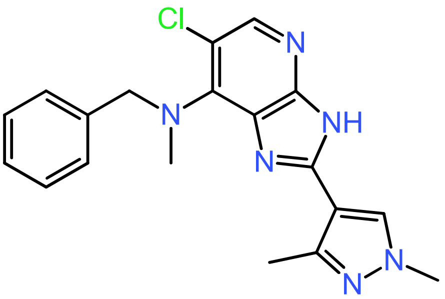

IC50(nM):169
fringNplaN4B:0
fsp3Csp2N5B:1
N_H_2B:4
fsp2Osp2C5B:0
da_lipo_5B:4
fringNC6B:2
fOringC6B:0

188 1605300

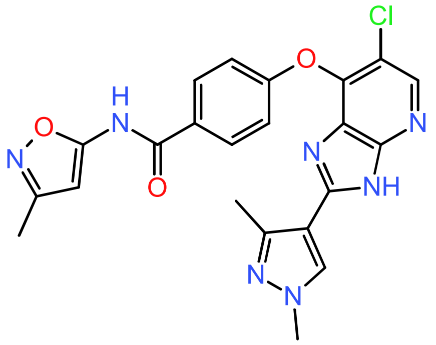

IC50(nM):2811.5
fringNplaN4B:0
fsp3Csp2N5B:0
N_H_2B:4
fsp2Osp2C5B:2
da_lipo_5B:11
fringNC6B:2
fOringC6B:2

189 1605303

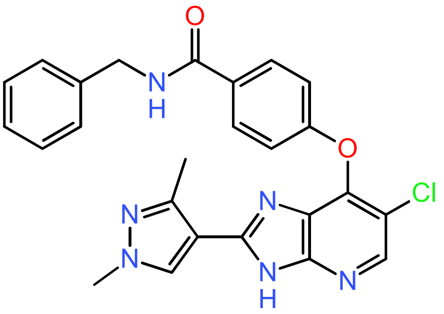

IC50(nM):3910
fringNplaN4B:0
fsp3Csp2N5B:0
N_H_2B:4
fsp2Osp2C5B:3
da_lipo_5B:15
fringNC6B:2
fOringC6B:4

190 1605304

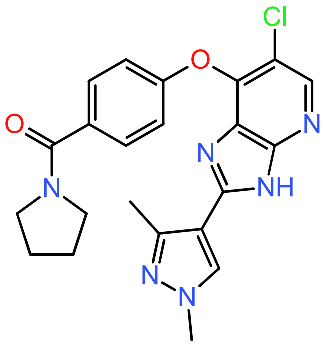

IC50(nM):1817
fringNplaN4B:0
fsp3Csp2N5B:0
N_H_2B:4
fsp2Osp2C5B:1
da_lipo_5B:4
fringNC6B:0
fOringC6B:2

191 1612940

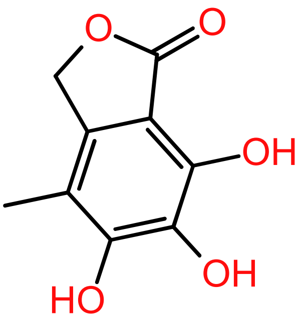

IC50(nM):8690
fringNplaN4B:0
fsp3Csp2N5B:0
N_H_2B:0
fsp2Osp2C5B:0
da_lipo_5B:4
fringNC6B:0
fOringC6B:0

192 1612941

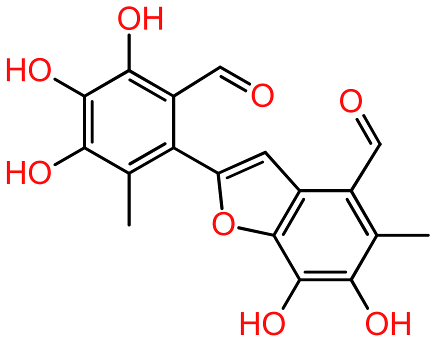

IC50(nM):7290
fringNplaN4B:0
fsp3Csp2N5B:0
N_H_2B:0
fsp2Osp2C5B:2
da_lipo_5B:9
fringNC6B:0
fOringC6B:0

193 1614604

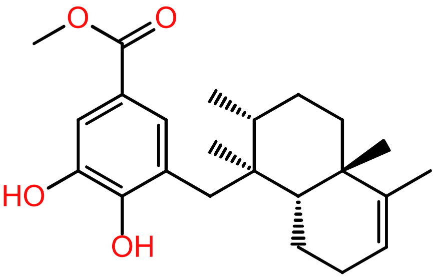

IC50(nM):3890
fringNplaN4B:0
fsp3Csp2N5B:0
N_H_2B:0
fsp2Osp2C5B:0
da_lipo_5B:9
fringNC6B:0
fOringC6B:3

194 1615168

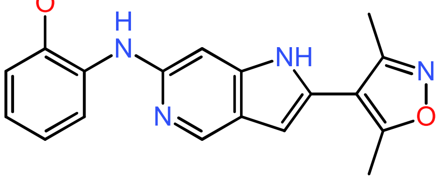

IC50(nM):16000
fringNplaN4B:0
fsp3Csp2N5B:0
N_H_2B:3
fsp2Osp2C5B:0
da_lipo_5B:10
fringNC6B:2
fOringC6B:1

195 1622333

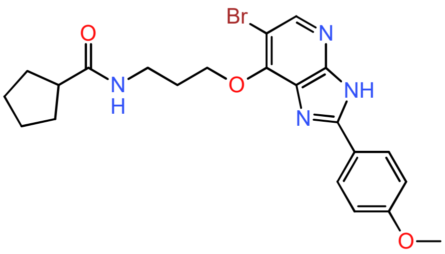

IC50(nM):156
fringNplaN4B:0
fsp3Csp2N5B:1
N_H_2B:3
fsp2Osp2C5B:0
da_lipo_5B:13
fringNC6B:1
fOringC6B:0

196 1622334

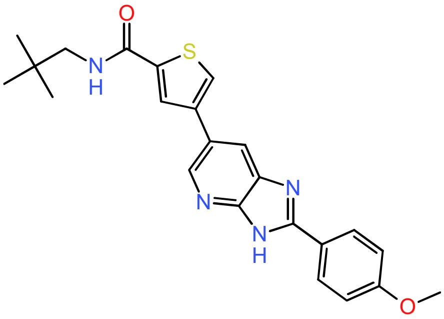

IC50(nM):757.5
fringNplaN4B:0
fsp3Csp2N5B:0
N_H_2B:3
fsp2Osp2C5B:1
da_lipo_5B:16
fringNC6B:1
fOringC6B:2

197 1622335

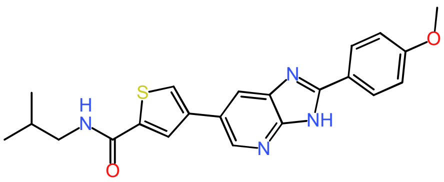

IC50(nM):2981.5
fringNplaN4B:0
fsp3Csp2N5B:0
N_H_2B:3
fsp2Osp2C5B:1
da_lipo_5B:15
fringNC6B:1
fOringC6B:2

198 1622337

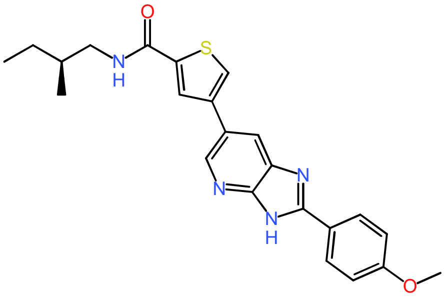

IC50(nM):2820
fringNplaN4B:0
fsp3Csp2N5B:0
N_H_2B:3
fsp2Osp2C5B:1
da_lipo_5B:16
fringNC6B:1
fOringC6B:2

199 1622338

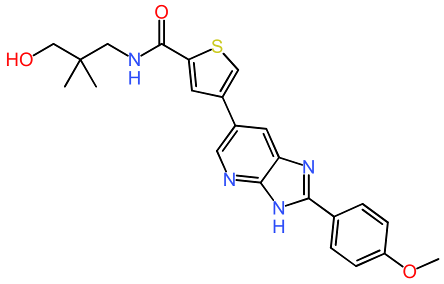

IC50(nM):1680
fringNplaN4B:0
fsp3Csp2N5B:0
N_H_2B:3
fsp2Osp2C5B:1
da_lipo_5B:15
fringNC6B:1
fOringC6B:2

200 1622339

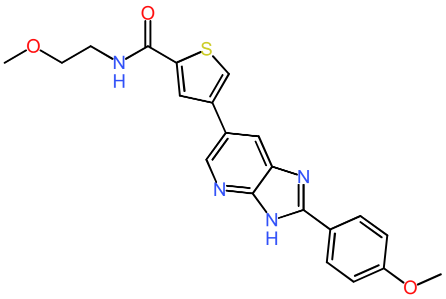

IC50(nM):1380
fringNplaN4B:0
fsp3Csp2N5B:0
N_H_2B:3
fsp2Osp2C5B:1
da_lipo_5B:12
fringNC6B:1
fOringC6B:2

201 1622340

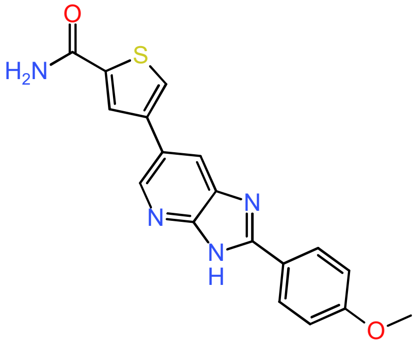

IC50(nM):2300
fringNplaN4B:0
fsp3Csp2N5B:0
N_H_2B:3
fsp2Osp2C5B:1
da_lipo_5B:12
fringNC6B:1
fOringC6B:2

202 1622342

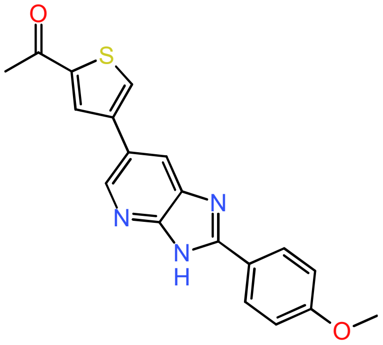

IC50(nM):12100
fringNplaN4B:0
fsp3Csp2N5B:0
N_H_2B:2
fsp2Osp2C5B:1
da_lipo_5B:8
fringNC6B:1
fOringC6B:2

203 1782650

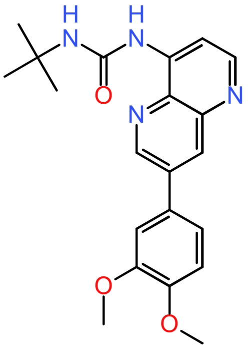

IC50(nM):714
fringNplaN4B:0
fsp3Csp2N5B:0
N_H_2B:4
fsp2Osp2C5B:2
da_lipo_5B:6
fringNC6B:2
fOringC6B:0

204 1782664

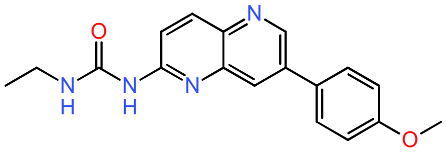

IC50(nM):2230
fringNplaN4B:1
fsp3Csp2N5B:1
N_H_2B:3
fsp2Osp2C5B:2
da_lipo_5B:5
fringNC6B:2
fOringC6B:3

205 1782665

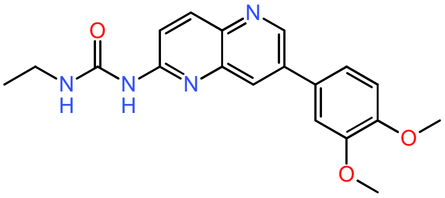

IC50(nM):624
fringNplaN4B:1
fsp3Csp2N5B:1
N_H_2B:3
fsp2Osp2C5B:2
da_lipo_5B:5
fringNC6B:2
fOringC6B:1

206 1782666

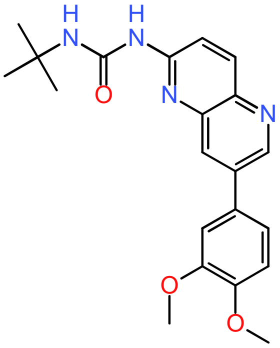

IC50(nM):805
fringNplaN4B:1
fsp3Csp2N5B:1
N_H_2B:3
fsp2Osp2C5B:2
da_lipo_5B:7
fringNC6B:4
fOringC6B:1

207 1782667

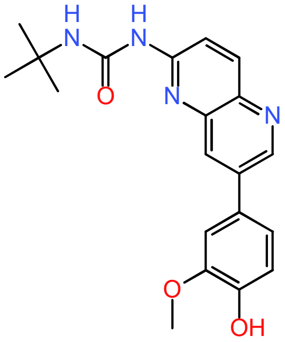

IC50(nM):227
fringNplaN4B:1
fsp3Csp2N5B:1
N_H_2B:3
fsp2Osp2C5B:2
da_lipo_5B:11
fringNC6B:4
fOringC6B:1

208 1782668

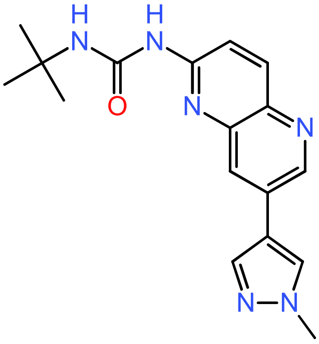

IC50(nM): 260
fringNplaN4B: 1
fsp3Csp2N5B: 1
N_H_2B: 5
fsp2Osp2C5B: 2
da_lipo_5B: 7
fringNC6B: 3
fOringC6B: 2

209 1782669

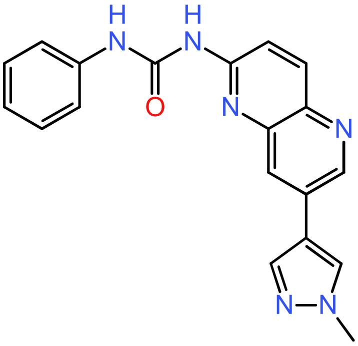

IC50(nM): 1140
fringNplaN4B: 1
fsp3Csp2N5B: 0
N_H_2B: 5
fsp2Osp2C5B: 4
da_lipo_5B: 9
fringNC6B: 2
fOringC6B: 3

210 1782671

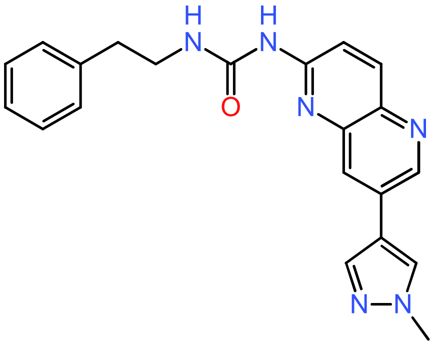

IC50(nM): 171
fringNplaN4B: 1
fsp3Csp2N5B: 1
N_H_2B: 5
fsp2Osp2C5B: 3
da_lipo_5B: 10
fringNC6B: 1
fOringC6B: 4

211 1782672

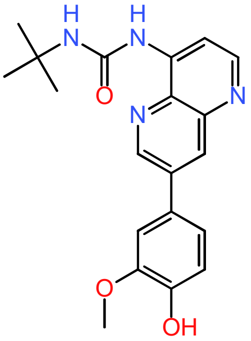

IC50(nM): 1580
fringNplaN4B: 0
fsp3Csp2N5B: 0
N_H_2B: 4
fsp2Osp2C5B: 2
da_lipo_5B: 10
fringNC6B: 2
fOringC6B: 0

212 1782673

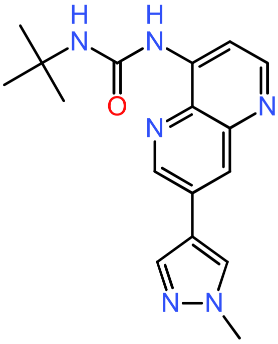

IC50(nM): 112
fringNplaN4B: 0
fsp3Csp2N5B: 0
N_H_2B: 6
fsp2Osp2C5B: 2
da_lipo_5B: 6
fringNC6B: 1
fOringC6B: 2

213 1782674

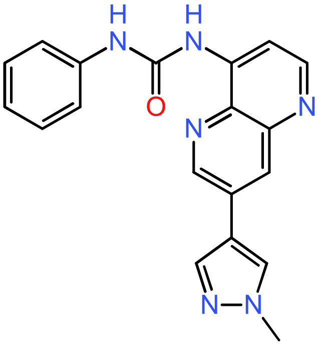

IC50(nM): 1260
fringNplaN4B: 0
fsp3Csp2N5B: 0
N_H_2B: 6
fsp2Osp2C5B: 4
da_lipo_5B: 8
fringNC6B: 1
fOringC6B: 3

214 1782675

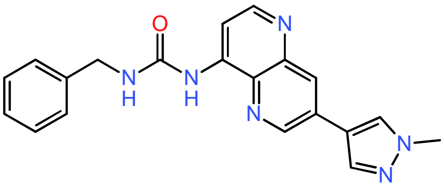

IC50(nM): 66
fringNplaN4B: 0
fsp3Csp2N5B: 0
N_H_2B: 6
fsp2Osp2C5B: 4
da_lipo_5B: 9
fringNC6B: 1
fOringC6B: 4

215 1782676

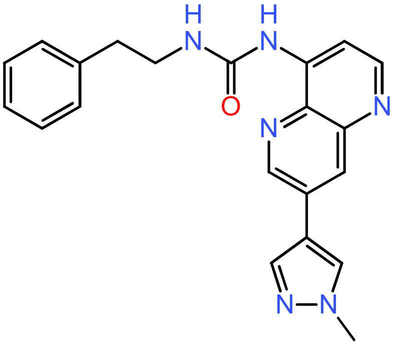

IC50(nM): 86
fringNplaN4B: 0
fsp3Csp2N5B: 0
N_H_2B: 6
fsp2Osp2C5B: 3
da_lipo_5B: 9
fringNC6B: 1
fOringC6B: 4

216 1785128

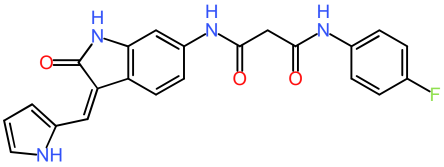

IC50(nM): 25
fringNplaN4B: 1
fsp3Csp2N5B: 0
N_H_2B: 4
fsp2Osp2C5B: 3
da_lipo_5B: 15
fringNC6B: 1
fOringC6B: 3

217 1785129

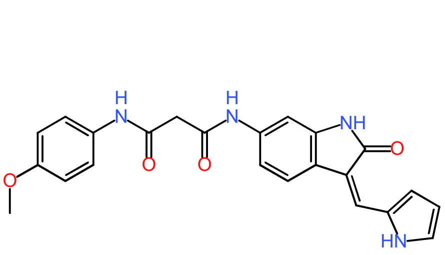

IC50(nM):29.6
fringNplaN4B:1
fsp3Csp2N5B:0
N_H_2B:4
fsp2Osp2C5B:3
da_lipo_5B: 13
fringNC6B:1
fOringC6B:2

218 1785130

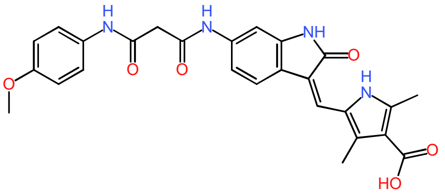

IC50(nM):5.9
fringNplaN4B:1
fsp3Csp2N5B:0
N_H_2B:4
fsp2Osp2C5B:3
da_lipo_5B: 15
fringNC6B:1
fOringC6B:0

219 1785132

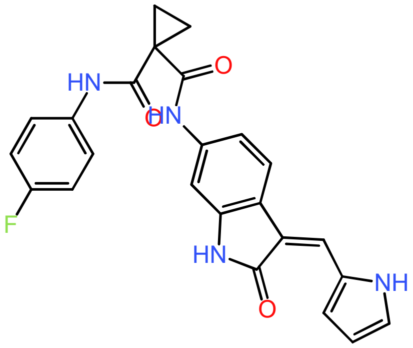

IC50(nM):252.6
fringNplaN4B:1
fsp3Csp2N5B:0
N_H_2B:4
fsp2Osp2C5B:3
da_lipo_5B: 17
fringNC6B:1
fOringC6B:3

220 1785133

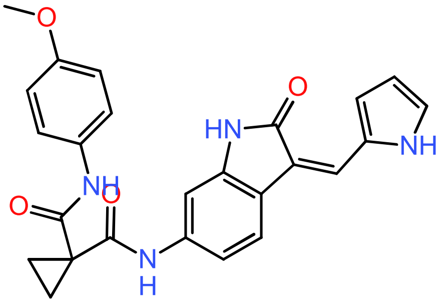

IC50(nM):347
fringNplaN4B:1
fsp3Csp2N5B:0
N_H_2B:4
fsp2Osp2C5B:3
da_lipo_5B: 15
fringNC6B:1
fOringC6B:2

221 1785138

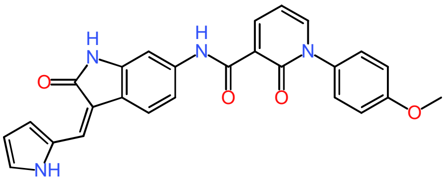

IC50(nM):209.3
fringNplaN4B:1
fsp3Csp2N5B:0
N_H_2B:4
fsp2Osp2C5B:3
da_lipo_5B: 11
fringNC6B:1
fOringC6B:2

222 1785139

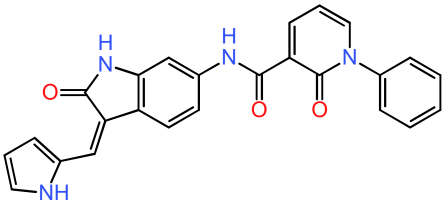

IC50(nM):87.4
fringNplaN4B:1
fsp3Csp2N5B:0
N_H_2B:4
fsp2Osp2C5B:3
da_lipo_5B: 11
fringNC6B:0
fOringC6B:3

223 1785140

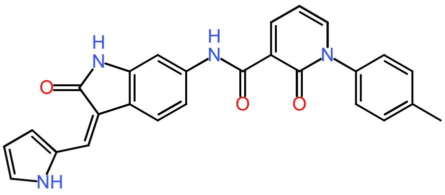

IC50(nM):209.4
fringNplaN4B:1
fsp3Csp2N5B:0
N_H_2B:4
fsp2Osp2C5B:3
da_lipo_5B: 11
fringNC6B:0
fOringC6B:3

224 1785143

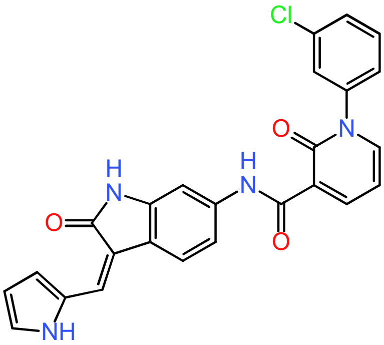

IC50(nM):250.6
fringNplaN4B:1
fsp3Csp2N5B:0
N_H_2B:4
fsp2Osp2C5B:3
da_lipo_5B: 11
fringNC6B:0
fOringC6B:3

225 1785144

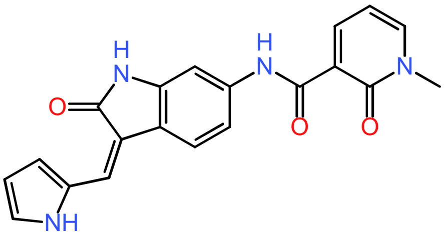

IC50(nM):102.2
fringNplaN4B:1
fsp3Csp2N5B:0
N_H_2B:4
fsp2Osp2C5B:1
da_lipo_5B: 11
fringNC6B:0
fOringC6B:2

226 1785145 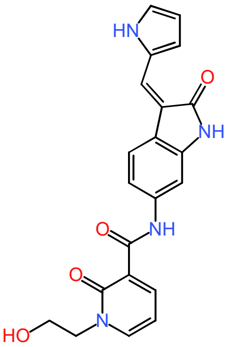 IC50(nM):32.5 fringNplaN4B:1 fsp3Csp2N5B:0 N_H_2B:4 fsp2Osp2C5B:1 da_lipo_5B:11 fringNC6B:0 fOringC6B:2	227 1785147 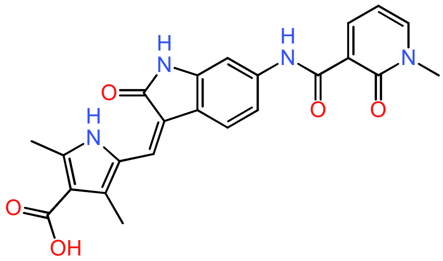 IC50(nM):14.3 fringNplaN4B:1 fsp3Csp2N5B:0 N_H_2B:4 fsp2Osp2C5B:1 da_lipo_5B:13 fringNC6B:0 fOringC6B:0	228 1785148 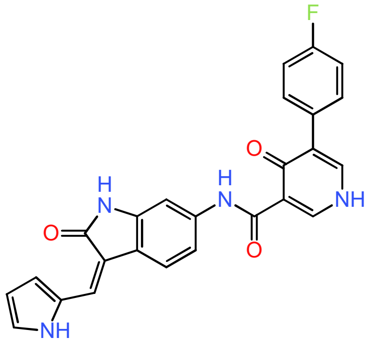 IC50(nM):127.6 fringNplaN4B:1 fsp3Csp2N5B:0 N_H_2B:4 fsp2Osp2C5B:3 da_lipo_5B:15 fringNC6B:1 fOringC6B:3
229 1785149 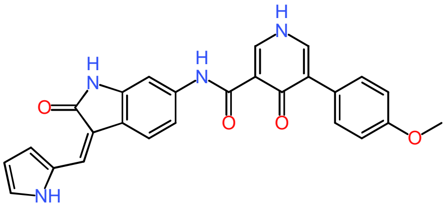 IC50(nM):10.2 fringNplaN4B:1 fsp3Csp2N5B:0 N_H_2B:4 fsp2Osp2C5B:3 da_lipo_5B:15 fringNC6B:1 fOringC6B:2	230 1785150 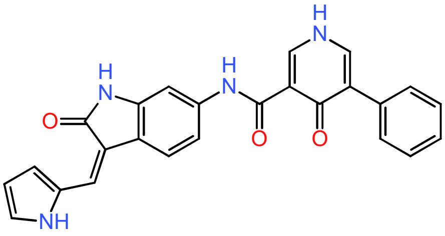 IC50(nM):33.1 fringNplaN4B:1 fsp3Csp2N5B:0 N_H_2B:4 fsp2Osp2C5B:3 da_lipo_5B:15 fringNC6B:1 fOringC6B:3	231 1785151 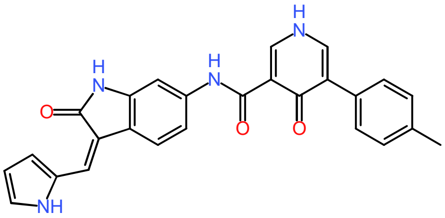 IC50(nM):63.2 fringNplaN4B:1 fsp3Csp2N5B:0 N_H_2B:4 fsp2Osp2C5B:3 da_lipo_5B:15 fringNC6B:1 fOringC6B:3
232 1785152 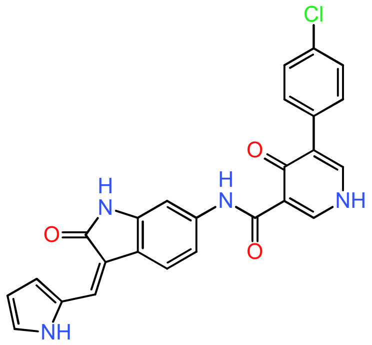 IC50(nM):53.8 fringNplaN4B:1 fsp3Csp2N5B:0 N_H_2B:4 fsp2Osp2C5B:3 da_lipo_5B:15 fringNC6B:1 fOringC6B:3	233 1785153 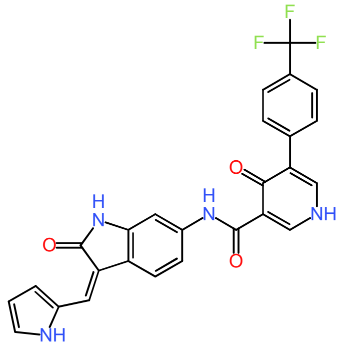 IC50(nM):348 fringNplaN4B:1 fsp3Csp2N5B:0 N_H_2B:4 fsp2Osp2C5B:3 da_lipo_5B:15 fringNC6B:1 fOringC6B:3	234 1785154 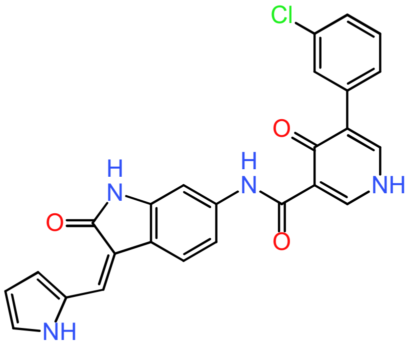 IC50(nM):161.4 fringNplaN4B:1 fsp3Csp2N5B:0 N_H_2B:4 fsp2Osp2C5B:3 da_lipo_5B:15 fringNC6B:1 fOringC6B:3

235 1785155

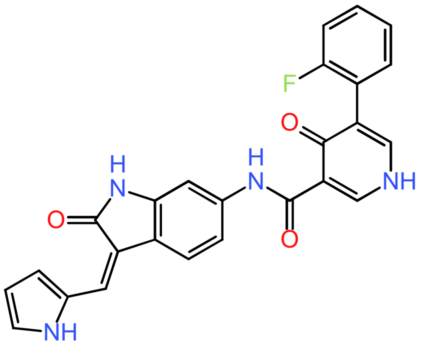

IC50(nM):25.5
fringNplaN4B:1
fsp3Csp2N5B:0
N_H_2B:4
fsp2Osp2C5B:3
da_lipo_5B: 16
fringNC6B:1
fOringC6B:3

236 1785156

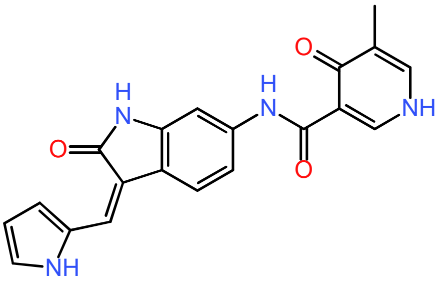

IC50(nM):29.2
fringNplaN4B:1
fsp3Csp2N5B:0
N_H_2B:4
fsp2Osp2C5B:1
da_lipo_5B: 11
fringNC6B:0
fOringC6B:2

237 1785504

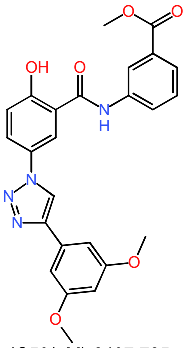

IC50(nM):2407.725
fringNplaN4B:0
fsp3Csp2N5B:0
N_H_2B:2
fsp2Osp2C5B:3
da_lipo_5B: 9
fringNC6B:3
fOringC6B:0

238 1788640

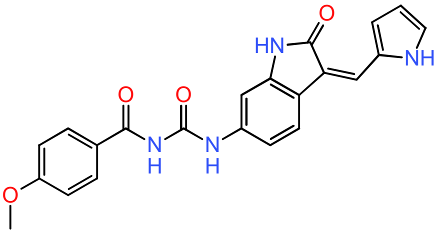

IC50(nM):54.45
fringNplaN4B:1
fsp3Csp2N5B:0
N_H_2B:4
fsp2Osp2C5B:2
da_lipo_5B: 13
fringNC6B:0
fOringC6B:2

239 1788644

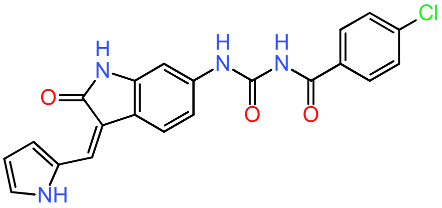

IC50(nM):6.5
fringNplaN4B:1
fsp3Csp2N5B:0
N_H_2B:4
fsp2Osp2C5B:2
da_lipo_5B: 14
fringNC6B:0
fOringC6B:2

240 1788645

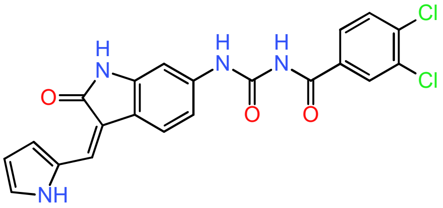

IC50(nM):7.7
fringNplaN4B:1
fsp3Csp2N5B:0
N_H_2B:4
fsp2Osp2C5B:2
da_lipo_5B: 15
fringNC6B:0
fOringC6B:2

241 1788646

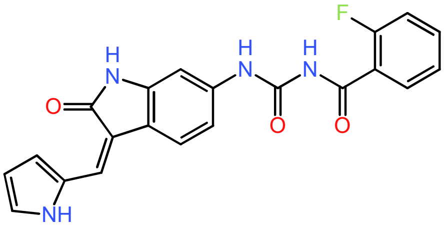

IC50(nM):6.5
fringNplaN4B:1
fsp3Csp2N5B:0
N_H_2B:4
fsp2Osp2C5B:2
da_lipo_5B: 15
fringNC6B:0
fOringC6B:2

242 1788647

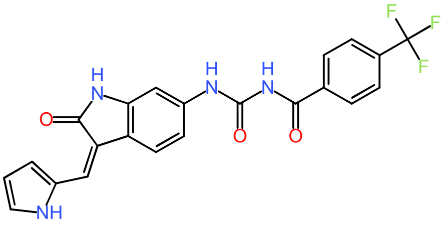

IC50(nM):40.9
fringNplaN4B:1
fsp3Csp2N5B:0
N_H_2B:4
fsp2Osp2C5B:2
da_lipo_5B: 14
fringNC6B:0
fOringC6B:2

243 1788648

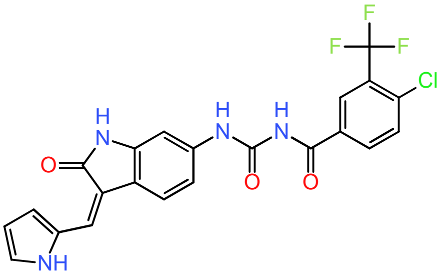

IC50(nM):15
fringNplaN4B:1
fsp3Csp2N5B:0
N_H_2B:4
fsp2Osp2C5B:2
da_lipo_5B: 15
fringNC6B:0
fOringC6B:2

244 1788649

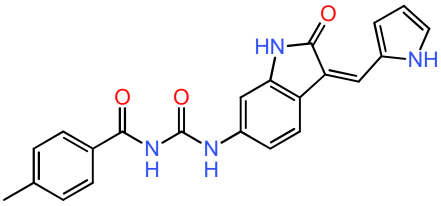

IC50(nM): 18.4
fringNplaN4B: 1
fsp3Csp2N5B: 0
N_H_2B: 4
fsp2Osp2C5B: 2
da_lipo_5B: 14
fringNC6B: 0
fOringC6B: 2

245 1788650

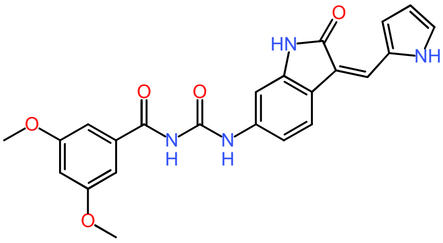

IC50(nM): 29.8
fringNplaN4B: 1
fsp3Csp2N5B: 0
N_H_2B: 4
fsp2Osp2C5B: 2
da_lipo_5B: 12
fringNC6B: 0
fOringC6B: 2

246 1788651

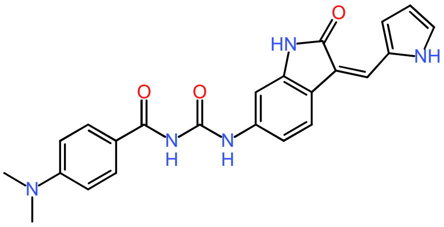

IC50(nM): 158.7
fringNplaN4B: 1
fsp3Csp2N5B: 0
N_H_2B: 5
fsp2Osp2C5B: 2
da_lipo_5B: 13
fringNC6B: 0
fOringC6B: 2

247 1788653

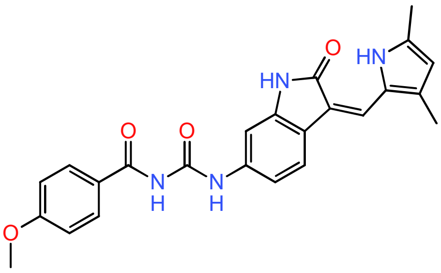

IC50(nM): 55.5
fringNplaN4B: 1
fsp3Csp2N5B: 0
N_H_2B: 4
fsp2Osp2C5B: 2
da_lipo_5B: 15
fringNC6B: 0
fOringC6B: 2

248 1788656

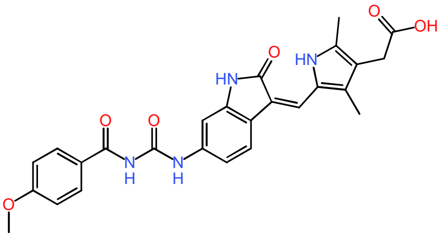

IC50(nM): 1.8
fringNplaN4B: 1
fsp3Csp2N5B: 0
N_H_2B: 4
fsp2Osp2C5B: 2
da_lipo_5B: 16
fringNC6B: 0
fOringC6B: 0

249 1788660

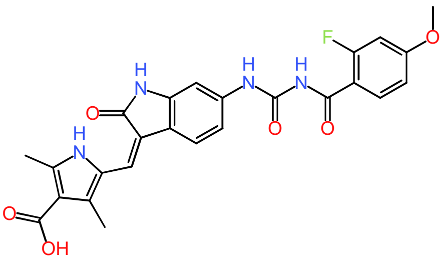

IC50(nM): 2.3
fringNplaN4B: 1
fsp3Csp2N5B: 0
N_H_2B: 4
fsp2Osp2C5B: 2
da_lipo_5B: 16
fringNC6B: 0
fOringC6B: 0

250 1795182

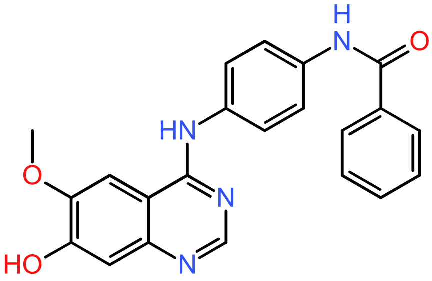

IC50(nM): 275
fringNplaN4B: 0
fsp3Csp2N5B: 0
N_H_2B: 4
fsp2Osp2C5B: 3
da_lipo_5B: 13
fringNC6B: 2
fOringC6B: 1

251 1795186

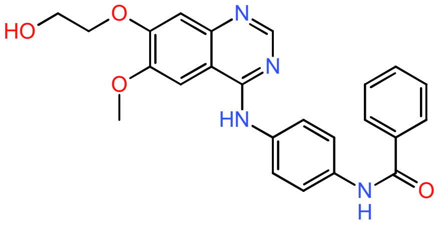

IC50(nM): 27.3
fringNplaN4B: 0
fsp3Csp2N5B: 1
N_H_2B: 4
fsp2Osp2C5B: 3
da_lipo_5B: 13
fringNC6B: 3
fOringC6B: 1

252 1795187

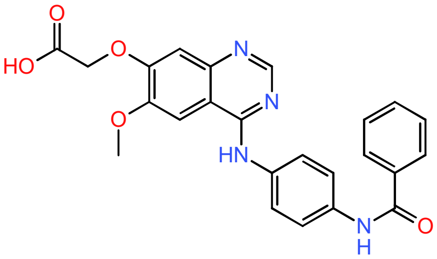

IC50(nM): 689
fringNplaN4B: 0
fsp3Csp2N5B: 1
N_H_2B: 4
fsp2Osp2C5B: 3
da_lipo_5B: 13
fringNC6B: 3
fOringC6B: 1

253 1795188

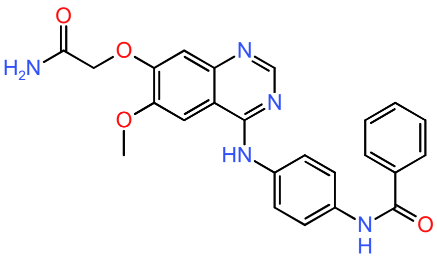

IC50(nM):155
fringNplaN4B:0
fsp3Csp2N5B:1
N_H_2B:5
fsp2Osp2C5B:5
da_lipo_5B:13
fringNC6B:3
fOringC6B:1

254 1817447

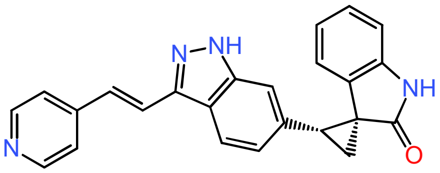

IC50(nM):7.4
fringNplaN4B:0
fsp3Csp2N5B:1
N_H_2B:4
fsp2Osp2C5B:4
da_lipo_5B:16
fringNC6B:0
fOringC6B:2

255 1817451

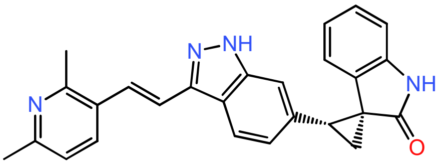

IC50(nM):10
fringNplaN4B:0
fsp3Csp2N5B:1
N_H_2B:3
fsp2Osp2C5B:4
da_lipo_5B:16
fringNC6B:0
fOringC6B:2

256 1817461

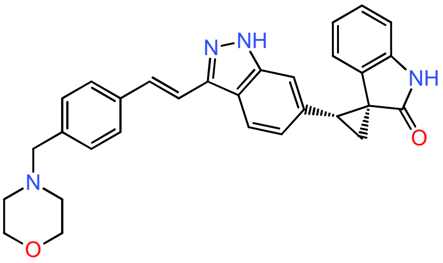

IC50(nM):49
fringNplaN4B:0
fsp3Csp2N5B:1
N_H_2B:4
fsp2Osp2C5B:4
da_lipo_5B:16
fringNC6B:0
fOringC6B:4

257 1817469

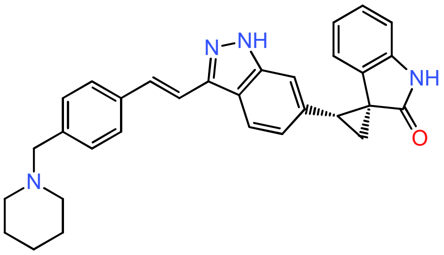

IC50(nM):14
fringNplaN4B:0
fsp3Csp2N5B:1
N_H_2B:4
fsp2Osp2C5B:4
da_lipo_5B:16
fringNC6B:0
fOringC6B:2

258 1818303

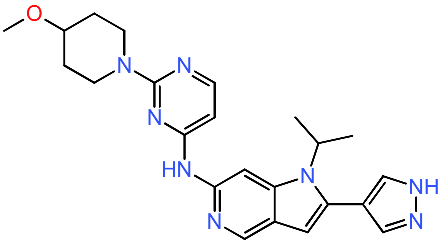

IC50(nM):107
fringNplaN4B:0
fsp3Csp2N5B:2
N_H_2B:7
fsp2Osp2C5B:0
da_lipo_5B:5
fringNC6B:0
fOringC6B:0

259 1820426

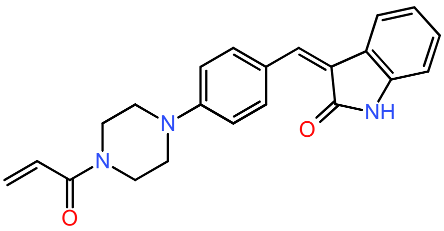

IC50(nM):586.15
fringNplaN4B:0
fsp3Csp2N5B:0
N_H_2B:3
fsp2Osp2C5B:4
da_lipo_5B:10
fringNC6B:0
fOringC6B:3

260 1820427

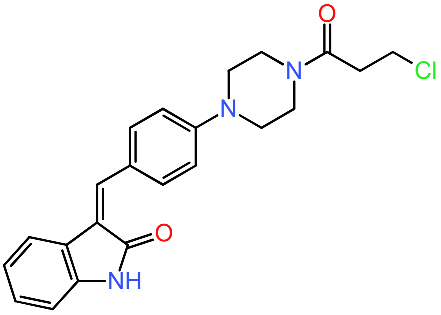

IC50(nM):185.8
fringNplaN4B:0
fsp3Csp2N5B:0
N_H_2B:3
fsp2Osp2C5B:4
da_lipo_5B:10
fringNC6B:0
fOringC6B:3

261 1825314

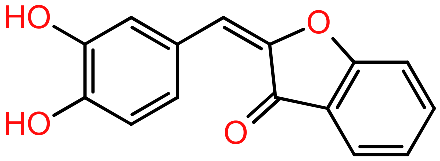

IC50(nM):1897.5
fringNplaN4B:0
fsp3Csp2N5B:0
N_H_2B:0
fsp2Osp2C5B:3
da_lipo_5B:5
fringNC6B:0
fOringC6B:0

262 1827269

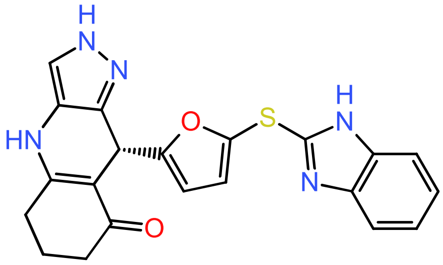

IC50(nM): 500
fringNplaN4B: 0
fsp3Csp2N5B: 0
N_H_2B: 4
fsp2Osp2C5B: 2
da_lipo_5B: 12
fringNC6B: 0
fOringC6B: 2

263 1827274

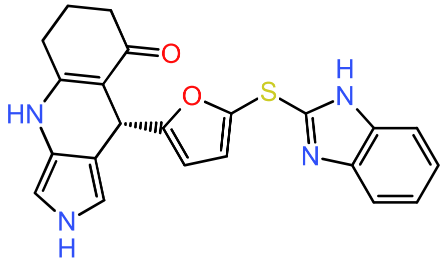

IC50(nM): 127
fringNplaN4B: 0
fsp3Csp2N5B: 0
N_H_2B: 3
fsp2Osp2C5B: 3
da_lipo_5B: 13
fringNC6B: 0
fOringC6B: 2

264 1827275

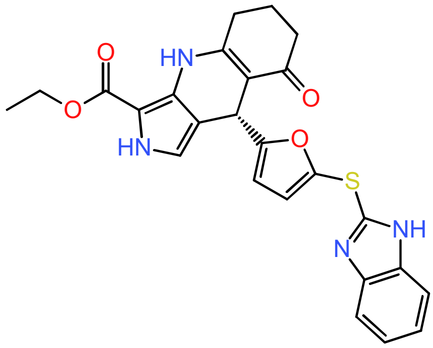

IC50(nM): 170.3333333
fringNplaN4B: 0
fsp3Csp2N5B: 0
N_H_2B: 3
fsp2Osp2C5B: 3
da_lipo_5B: 14
fringNC6B: 0
fOringC6B: 2

265 1827304

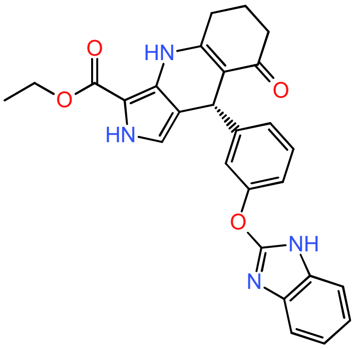

IC50(nM): 16.33
fringNplaN4B: 0
fsp3Csp2N5B: 0
N_H_2B: 3
fsp2Osp2C5B: 4
da_lipo_5B: 16
fringNC6B: 0
fOringC6B: 0

266 1840536

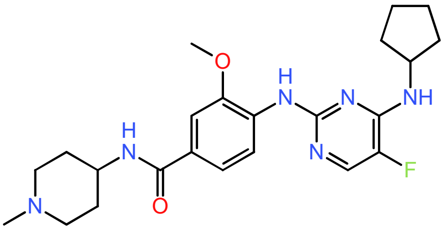

IC50(nM): 395
fringNplaN4B: 1
fsp3Csp2N5B: 1
N_H_2B: 5
fsp2Osp2C5B: 1
da_lipo_5B: 12
fringNC6B: 2
fOringC6B: 2

267 1928549

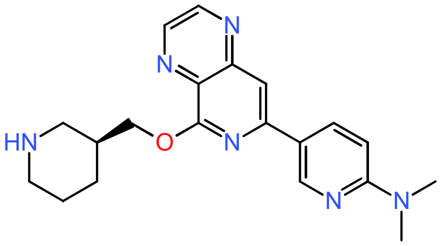

IC50(nM): 63.1
fringNplaN4B: 0
fsp3Csp2N5B: 1
N_H_2B: 5
fsp2Osp2C5B: 0
da_lipo_5B: 3
fringNC6B: 0
fOringC6B: 1

268 1949692

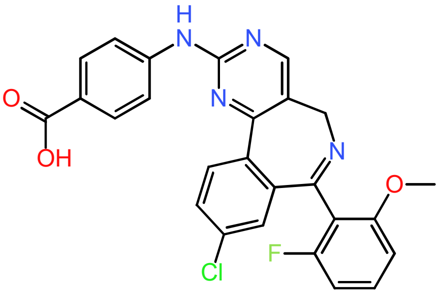

IC50(nM): 2500
fringNplaN4B: 0
fsp3Csp2N5B: 0
N_H_2B: 3
fsp2Osp2C5B: 0
da_lipo_5B: 9
fringNC6B: 1
fOringC6B: 4

269 1955097

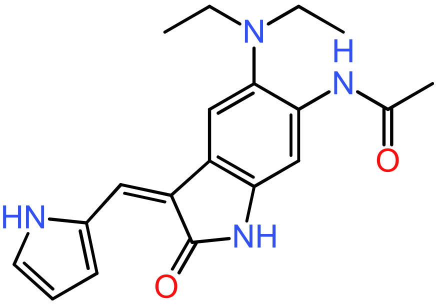

IC50(nM): 1291.5
fringNplaN4B: 1
fsp3Csp2N5B: 0
N_H_2B: 4
fsp2Osp2C5B: 1
da_lipo_5B: 10
fringNC6B: 3
fOringC6B: 2

270 1955098

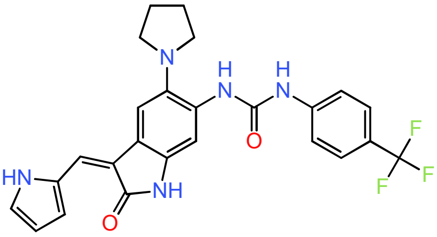

IC50(nM): 948.5
fringNplaN4B: 0
fsp3Csp2N5B: 0
N_H_2B: 5
fsp2Osp2C5B: 3
da_lipo_5B: 15
fringNC6B: 1
fOringC6B: 5

271 1965882

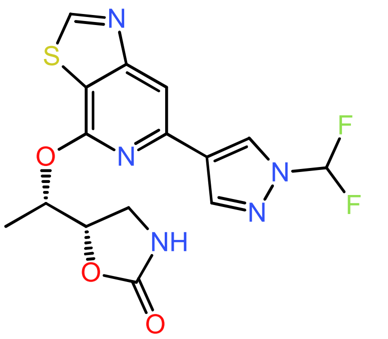

IC50(nM): 110
fringNplaN4B: 0
fsp3Csp2N5B: 1
N_H_2B: 4
fsp2Osp2C5B: 0
da_lipo_5B: 1
fringNC6B: 0
fOringC6B: 0

272 1966643

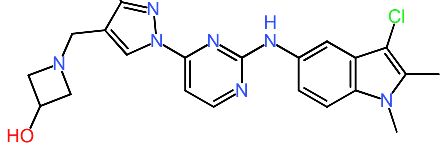

IC50(nM): 1711
fringNplaN4B: 0
fsp3Csp2N5B: 1
N_H_2B: 5
fsp2Osp2C5B: 0
da_lipo_5B: 8
fringNC6B: 0
fOringC6B: 2

273 1966645

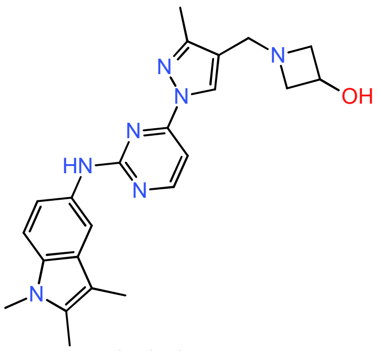

IC50(nM): 985.2
fringNplaN4B: 0
fsp3Csp2N5B: 1
N_H_2B: 5
fsp2Osp2C5B: 0
da_lipo_5B: 8
fringNC6B: 0
fOringC6B: 2

274 1978966

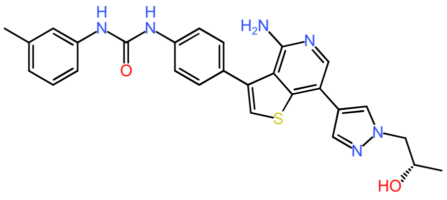

IC50(nM): 11.21
fringNplaN4B: 0
fsp3Csp2N5B: 0
N_H_2B: 6
fsp2Osp2C5B: 4
da_lipo_5B: 19
fringNC6B: 2
fOringC6B: 3

275 2003425

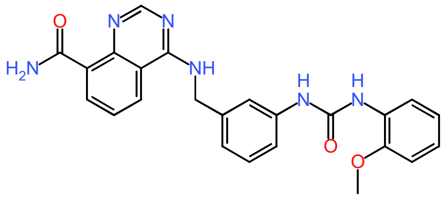

IC50(nM): 2.5
fringNplaN4B: 1
fsp3Csp2N5B: 1
N_H_2B: 6
fsp2Osp2C5B: 7
da_lipo_5B: 14
fringNC6B: 2
fOringC6B: 1

276 2003426

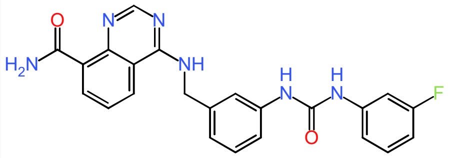

IC50(nM): 5.6
fringNplaN4B: 1
fsp3Csp2N5B: 1
N_H_2B: 6
fsp2Osp2C5B: 7
da_lipo_5B: 16
fringNC6B: 2
fOringC6B: 2

277 2003428

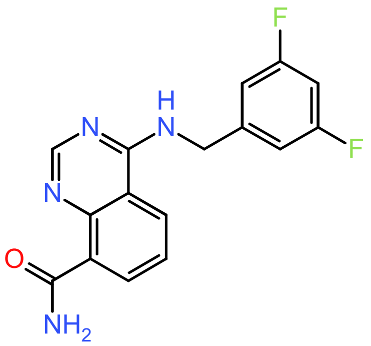

IC50(nM): 3.9
fringNplaN4B: 1
fsp3Csp2N5B: 1
N_H_2B: 4
fsp2Osp2C5B: 3
da_lipo_5B: 13
fringNC6B: 2
fOringC6B: 0

278 2003440

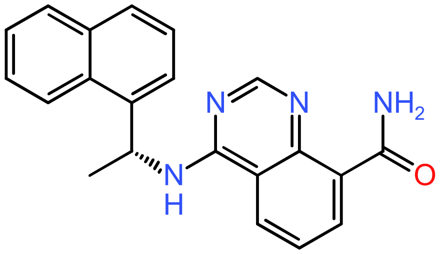

IC50(nM): 11
fringNplaN4B: 1
fsp3Csp2N5B: 1
N_H_2B: 4
fsp2Osp2C5B: 3
da_lipo_5B: 15
fringNC6B: 3
fOringC6B: 0

279 2003446

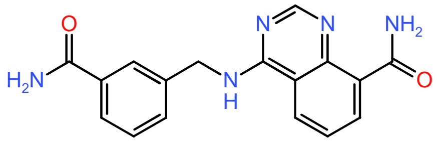

IC50(nM): 4.1
fringNplaN4B: 1
fsp3Csp2N5B: 1
N_H_2B: 5
fsp2Osp2C5B: 4
da_lipo_5B: 11
fringNC6B: 2
fOringC6B: 0

280 2003455

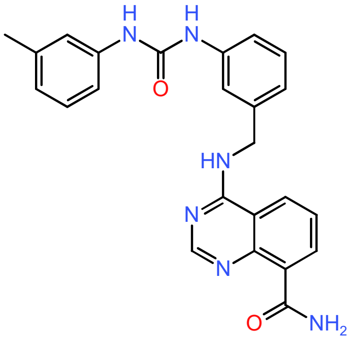

IC50(nM):4.3
fringNplaN4B:1
fsp3Csp2N5B:1
N_H_2B:6
fsp2Osp2C5B:7
da_lipo_5B:16
fringNC6B:2
fOringC6B:2

281 2003466

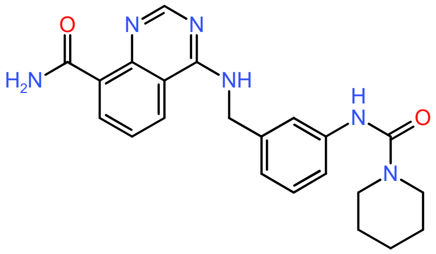

IC50(nM):2.2
fringNplaN4B:1
fsp3Csp2N5B:1
N_H_2B:6
fsp2Osp2C5B:5
da_lipo_5B:13
fringNC6B:0
fOringC6B:1

282 2003467

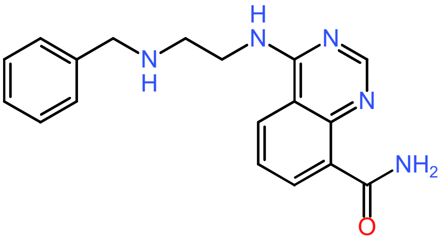

IC50(nM):23
fringNplaN4B:1
fsp3Csp2N5B:1
N_H_2B:5
fsp2Osp2C5B:3
da_lipo_5B:11
fringNC6B:1
fOringC6B:0

283 2003469

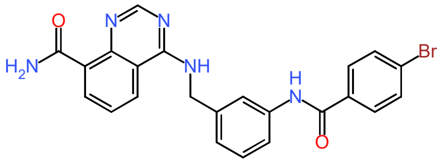

IC50(nM):0.71
fringNplaN4B:1
fsp3Csp2N5B:1
N_H_2B:5
fsp2Osp2C5B:6
da_lipo_5B:16
fringNC6B:2
fOringC6B:1

284 2003471

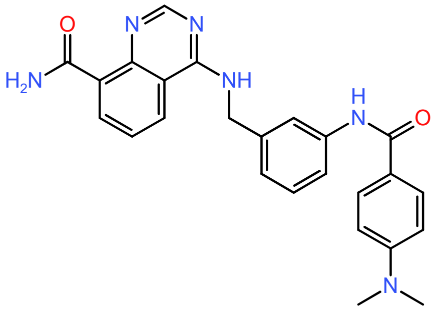

IC50(nM):0.52
fringNplaN4B:1
fsp3Csp2N5B:1
N_H_2B:6
fsp2Osp2C5B:6
da_lipo_5B:15
fringNC6B:2
fOringC6B:1

285 2003472

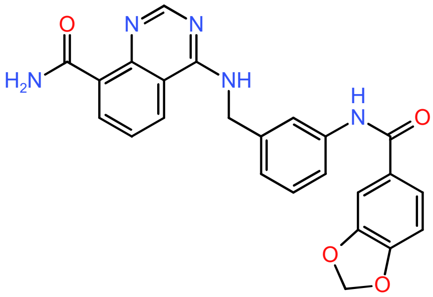

IC50(nM):1.2
fringNplaN4B:1
fsp3Csp2N5B:1
N_H_2B:5
fsp2Osp2C5B:6
da_lipo_5B:14
fringNC6B:2
fOringC6B:1

286 2003473

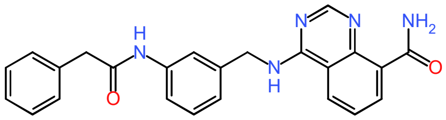

IC50(nM):2.1
fringNplaN4B:1
fsp3Csp2N5B:1
N_H_2B:5
fsp2Osp2C5B:7
da_lipo_5B:16
fringNC6B:2
fOringC6B:2

287 2003474

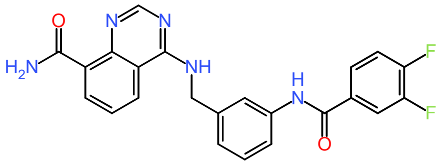

IC50(nM):3
fringNplaN4B:1
fsp3Csp2N5B:1
N_H_2B:5
fsp2Osp2C5B:6
da_lipo_5B:17
fringNC6B:2
fOringC6B:1

288 2003475

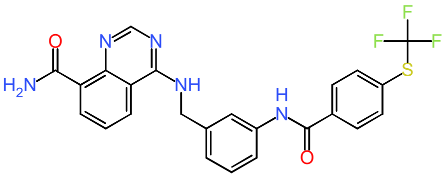

IC50(nM):1.2
fringNplaN4B:1
fsp3Csp2N5B:1
N_H_2B:5
fsp2Osp2C5B:6
da_lipo_5B:16
fringNC6B:2
fOringC6B:1

289 2003477

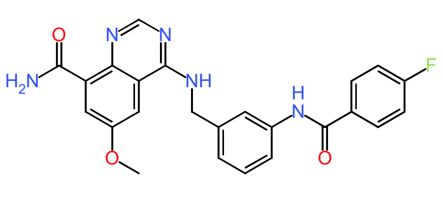

IC50(nM): 1.7
fringNplaN4B: 1
fsp3Csp2N5B: 1
N_H_2B: 5
fsp2Osp2C5B: 6
da_lipo_5B: 15
fringNC6B: 3
fOringC6B: 1

290 2003479

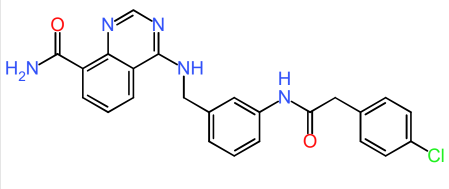

IC50(nM): 1.6
fringNplaN4B: 1
fsp3Csp2N5B: 1
N_H_2B: 5
fsp2Osp2C5B: 7
da_lipo_5B: 16
fringNC6B: 2
fOringC6B: 2

291 2003480

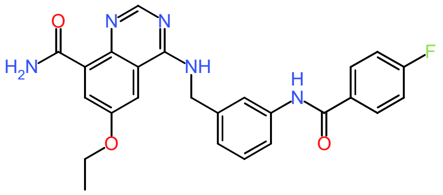

IC50(nM): 7.8
fringNplaN4B: 1
fsp3Csp2N5B: 1
N_H_2B: 5
fsp2Osp2C5B: 6
da_lipo_5B: 15
fringNC6B: 3
fOringC6B: 1

292 2003486

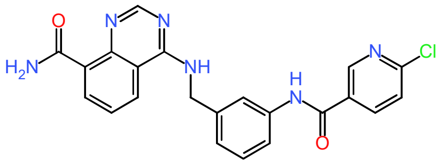

IC50(nM): 0.46
fringNplaN4B: 2
fsp3Csp2N5B: 1
N_H_2B: 6
fsp2Osp2C5B: 6
da_lipo_5B: 13
fringNC6B: 2
fOringC6B: 1

293 2003488

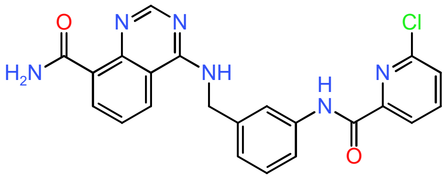

IC50(nM): 2.9
fringNplaN4B: 1
fsp3Csp2N5B: 1
N_H_2B: 5
fsp2Osp2C5B: 6
da_lipo_5B: 14
fringNC6B: 1
fOringC6B: 1

294 2003492

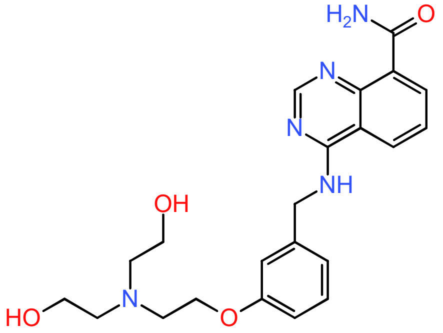

IC50(nM): 88
fringNplaN4B: 1
fsp3Csp2N5B: 1
N_H_2B: 5
fsp2Osp2C5B: 3
da_lipo_5B: 10
fringNC6B: 2
fOringC6B: 0

295 2003494

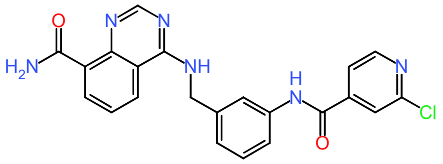

IC50(nM): 25
fringNplaN4B: 1
fsp3Csp2N5B: 1
N_H_2B: 6
fsp2Osp2C5B: 5
da_lipo_5B: 14
fringNC6B: 2
fOringC6B: 1

296 2003498

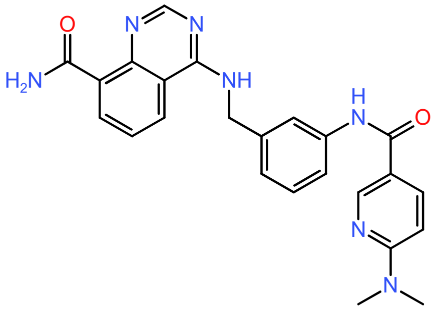

IC50(nM): 0.94
fringNplaN4B: 2
fsp3Csp2N5B: 1
N_H_2B: 7
fsp2Osp2C5B: 6
da_lipo_5B: 13
fringNC6B: 2
fOringC6B: 1

297 2003499

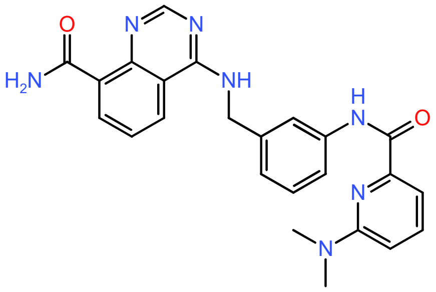

IC50(nM): 0.73
fringNplaN4B: 1
fsp3Csp2N5B: 1
N_H_2B: 6
fsp2Osp2C5B: 6
da_lipo_5B: 13
fringNC6B: 1
fOringC6B: 1

298 2003500

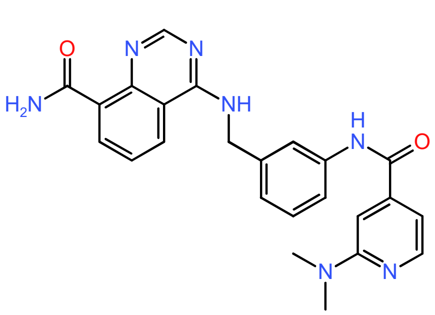

IC50(nM):2.6
fringNplaN4B:1
fsp3Csp2N5B:1
N_H_2B:7
fsp2Osp2C5B:5
da_lipo_5B:13
fringNC6B:2
fOringC6B:1

299 2005920

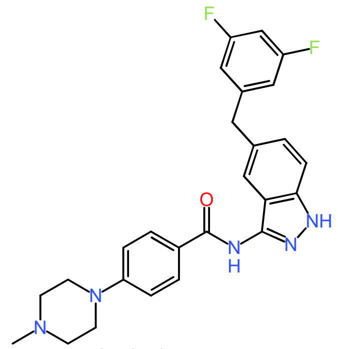

IC50(nM):484
fringNplaN4B:0
fsp3Csp2N5B:1
N_H_2B:5
fsp2Osp2C5B:3
da_lipo_5B:11
fringNC6B:1
fOringC6B:2

300 2008378

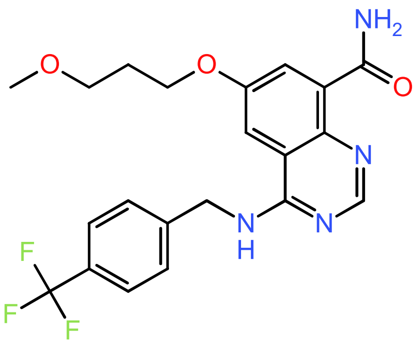

IC50(nM):170
fringNplaN4B:1
fsp3Csp2N5B:1
N_H_2B:4
fsp2Osp2C5B:3
da_lipo_5B:10
fringNC6B:3
fOringC6B:0

301 2008379

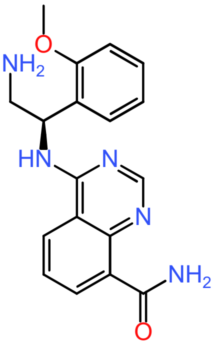

IC50(nM):48
fringNplaN4B:1
fsp3Csp2N5B:1
N_H_2B:5
fsp2Osp2C5B:3
da_lipo_5B:10
fringNC6B:2
fOringC6B:0

302 2008380

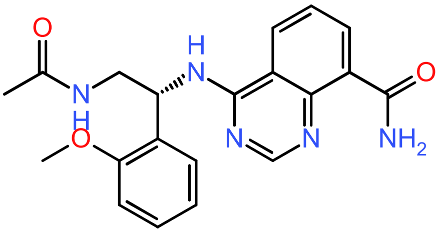

IC50(nM):7.1
fringNplaN4B:1
fsp3Csp2N5B:1
N_H_2B:5
fsp2Osp2C5B:4
da_lipo_5B:11
fringNC6B:3
fOringC6B:0

303 2008403

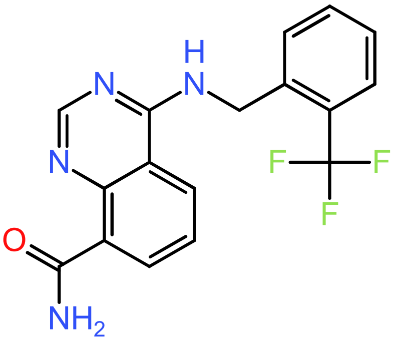

IC50(nM):7.6
fringNplaN4B:1
fsp3Csp2N5B:1
N_H_2B:4
fsp2Osp2C5B:3
da_lipo_5B:15
fringNC6B:3
fOringC6B:0

304 2008422

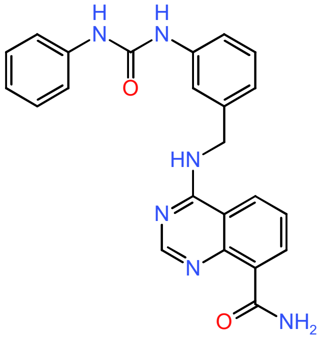

IC50(nM):1.5
fringNplaN4B:1
fsp3Csp2N5B:1
N_H_2B:6
fsp2Osp2C5B:7
da_lipo_5B:15
fringNC6B:2
fOringC6B:2

305 2008430

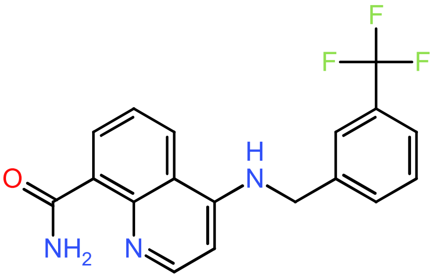

IC50(nM):12
fringNplaN4B:2
fsp3Csp2N5B:1
N_H_2B:3
fsp2Osp2C5B:3
da_lipo_5B:13
fringNC6B:1
fOringC6B:1

306 2008432

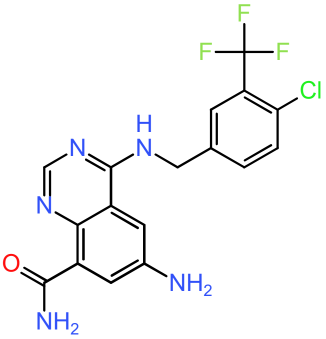

IC50(nM):13
fringNplaN4B:1
fsp3Csp2N5B:1
N_H_2B:5
fsp2Osp2C5B:3
da_lipo_5B:11
fringNC6B:2
fOringC6B:0

307 2008495

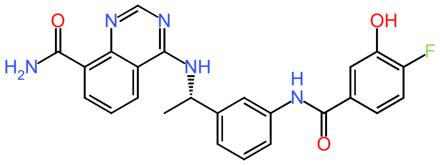

IC50(nM):0.4
fringNplaN4B:1
fsp3Csp2N5B:1
N_H_2B:5
fsp2Osp2C5B:6
da_lipo_5B:17
fringNC6B:2
fOringC6B:1

308 2008497

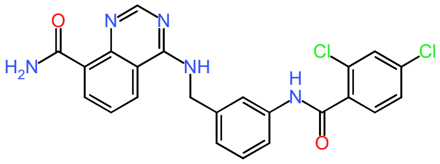

IC50(nM):0.35
fringNplaN4B:1
fsp3Csp2N5B:1
N_H_2B:5
fsp2Osp2C5B:6
da_lipo_5B:17
fringNC6B:2
fOringC6B:1

309 2008505

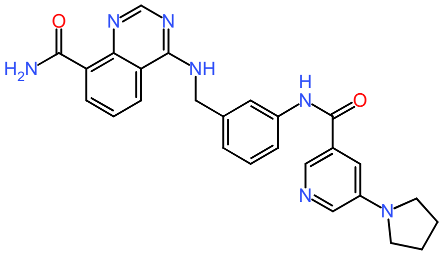

IC50(nM):1.1
fringNplaN4B:2
fsp3Csp2N5B:1
N_H_2B:7
fsp2Osp2C5B:6
da_lipo_5B:12
fringNC6B:2
fOringC6B:3

310 2008517

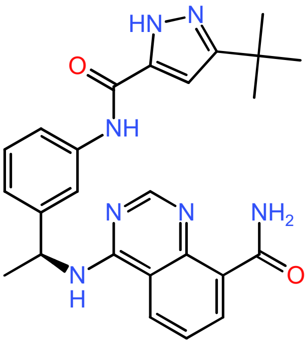

IC50(nM):1.3
fringNplaN4B:1
fsp3Csp2N5B:1
N_H_2B:7
fsp2Osp2C5B:5
da_lipo_5B:16
fringNC6B:1
fOringC6B:1

311 2008518

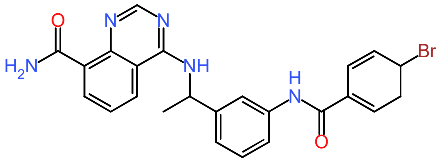

IC50(nM):0.66
fringNplaN4B:1
fsp3Csp2N5B:1
N_H_2B:5
fsp2Osp2C5B:5
da_lipo_5B:17
fringNC6B:2
fOringC6B:1

312 2008530

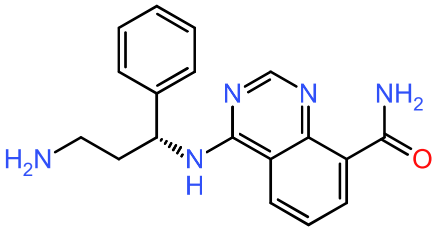

IC50(nM):9.6
fringNplaN4B:1
fsp3Csp2N5B:1
N_H_2B:5
fsp2Osp2C5B:3
da_lipo_5B:12
fringNC6B:2
fOringC6B:0

313 2008539

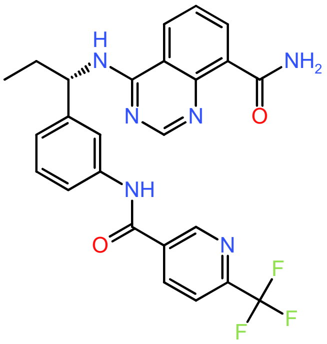

IC50(nM):1.4
fringNplaN4B:2
fsp3Csp2N5B:1
N_H_2B:6
fsp2Osp2C5B:6
da_lipo_5B:15
fringNC6B:2
fOringC6B:1

314 2008543

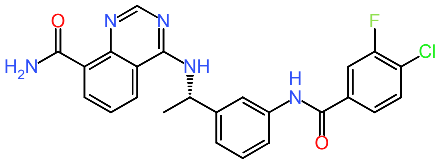

IC50(nM):1.2
fringNplaN4B:1
fsp3Csp2N5B:1
N_H_2B:5
fsp2Osp2C5B:6
da_lipo_5B:18
fringNC6B:2
fOringC6B:1

315 2008545

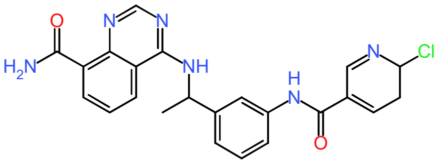

IC50(nM):0.4
fringNplaN4B:2
fsp3Csp2N5B:1
N_H_2B:6
fsp2Osp2C5B:5
da_lipo_5B:14
fringNC6B:2
fOringC6B:1

316 2008553

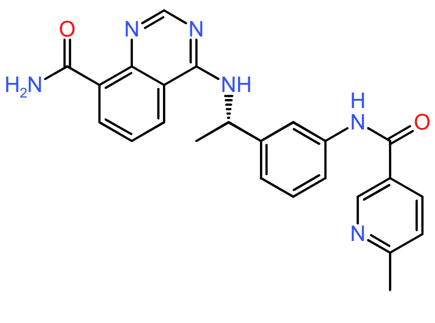

IC50(nM):0.32
fringNplaN4B:2
fsp3Csp2N5B:1
N_H_2B:6
fsp2Osp2C5B:6
da_lipo_5B:14
fringNC6B:2
fOringC6B:1

317 2008558

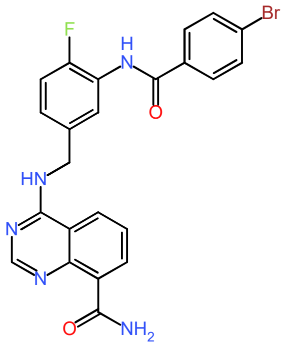

IC50(nM):0.69
fringNplaN4B:1
fsp3Csp2N5B:1
N_H_2B:5
fsp2Osp2C5B:6
da_lipo_5B:17
fringNC6B:2
fOringC6B:1

318 2008572

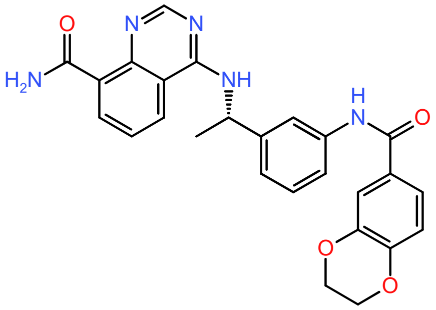

IC50(nM):0.89
fringNplaN4B:1
fsp3Csp2N5B:1
N_H_2B:5
fsp2Osp2C5B:6
da_lipo_5B:15
fringNC6B:2
fOringC6B:1

319 2008590

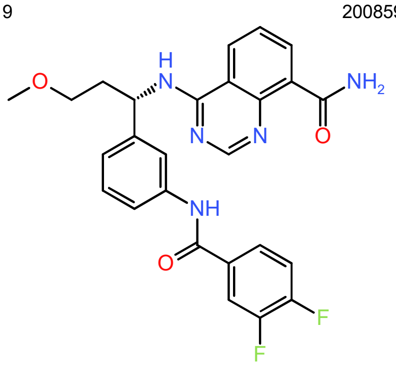

IC50(nM):3.1
fringNplaN4B:1
fsp3Csp2N5B:1
N_H_2B:5
fsp2Osp2C5B:6
da_lipo_5B:18
fringNC6B:2
fOringC6B:0

320 2013489

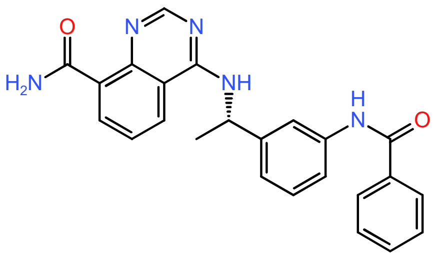

IC50(nM):2.4
fringNplaN4B:1
fsp3Csp2N5B:1
N_H_2B:5
fsp2Osp2C5B:6
da_lipo_5B:17
fringNC6B:2
fOringC6B:1

321 2013493

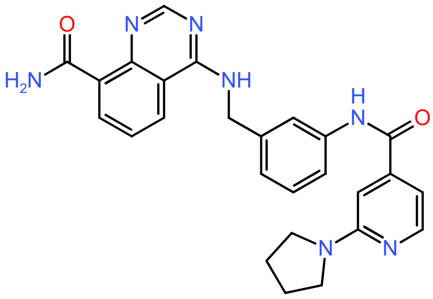

IC50(nM):2.1
fringNplaN4B:1
fsp3Csp2N5B:1
N_H_2B:7
fsp2Osp2C5B:5
da_lipo_5B:13
fringNC6B:2
fOringC6B:3

322 2013494

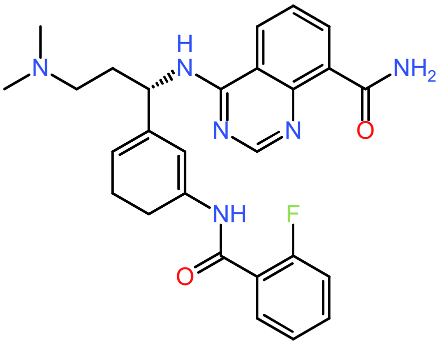

IC50(nM):7.8
fringNplaN4B:1
fsp3Csp2N5B:1
N_H_2B:6
fsp2Osp2C5B:5
da_lipo_5B:18
fringNC6B:2
fOringC6B:1

323 2013512

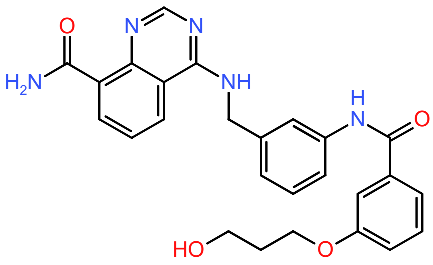

IC50(nM):13
fringNplaN4B:1
fsp3Csp2N5B:1
N_H_2B:5
fsp2Osp2C5B:6
da_lipo_5B:16
fringNC6B:2
fOringC6B:1

324 2013513

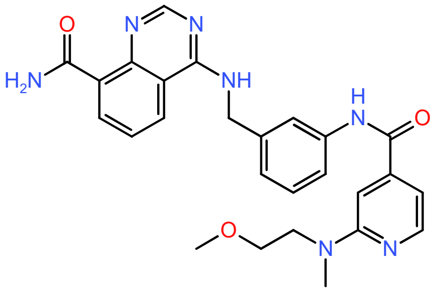

IC50(nM):3.5
fringNplaN4B:1
fsp3Csp2N5B:1
N_H_2B:7
fsp2Osp2C5B:5
da_lipo_5B:13
fringNC6B:3
fOringC6B:1

325 2013519

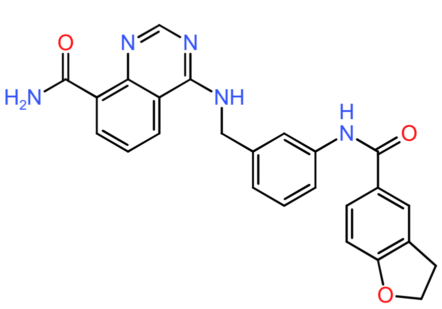

IC50(nM):0.46
fringNplaN4B:1
fsp3Csp2N5B:1
N_H_2B:5
fsp2Osp2C5B:6
da_lipo_5B:16
fringNC6B:2
fOringC6B:1

326 2013522

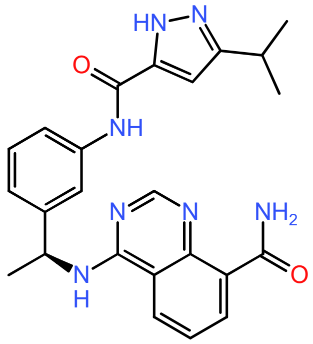

IC50(nM):0.27
fringNplaN4B:1
fsp3Csp2N5B:1
N_H_2B:7
fsp2Osp2C5B:5
da_lipo_5B:15
fringNC6B:1
fOringC6B:1

327 2013523

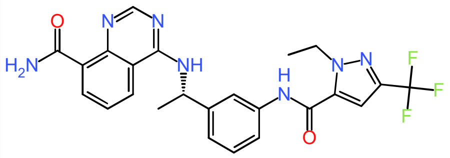

IC50(nM):0.58
fringNplaN4B:1
fsp3Csp2N5B:1
N_H_2B:6
fsp2Osp2C5B:5
da_lipo_5B:14
fringNC6B:1
fOringC6B:1

328 2013531

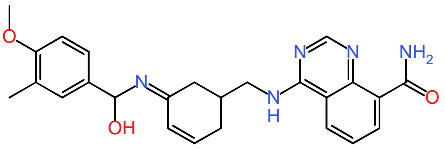

IC50(nM):3
fringNplaN4B:1
fsp3Csp2N5B:1
N_H_2B:5
fsp2Osp2C5B:3
da_lipo_5B:16
fringNC6B:2
fOringC6B:1

329 2013538

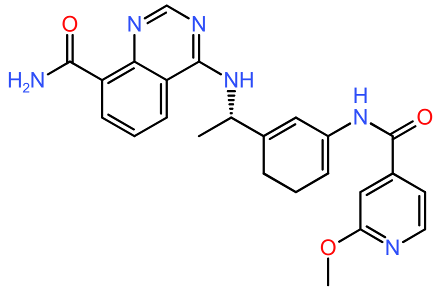

IC50(nM):2.3
fringNplaN4B:1
fsp3Csp2N5B:1
N_H_2B:6
fsp2Osp2C5B:4
da_lipo_5B:14
fringNC6B:2
fOringC6B:1

330 2013548

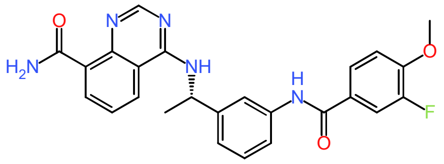

IC50(nM):0.57
fringNplaN4B:1
fsp3Csp2N5B:1
N_H_2B:5
fsp2Osp2C5B:6
da_lipo_5B:17
fringNC6B:2
fOringC6B:1

331 2013553

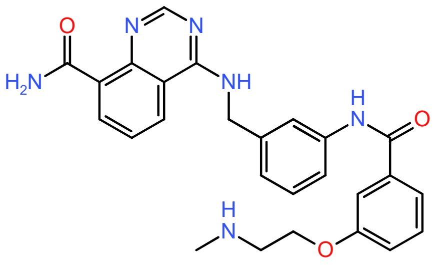

IC50(nM):13
fringNplaN4B:1
fsp3Csp2N5B:1
N_H_2B:6
fsp2Osp2C5B:6
da_lipo_5B:15
fringNC6B:2
fOringC6B:1

332 2013557

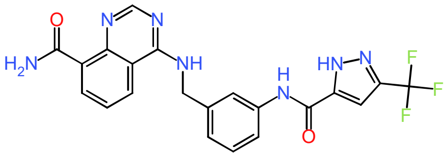

IC50(nM):0.51
fringNplaN4B:1
fsp3Csp2N5B:1
N_H_2B:7
fsp2Osp2C5B:5
da_lipo_5B:15
fringNC6B:1
fOringC6B:1

333 2013562

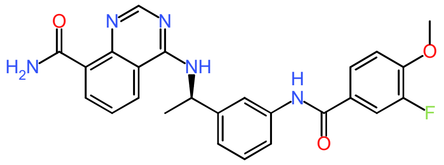

IC50(nM):0.41
fringNplaN4B:1
fsp3Csp2N5B:1
N_H_2B:5
fsp2Osp2C5B:6
da_lipo_5B:17
fringNC6B:2
fOringC6B:1

334 2013566

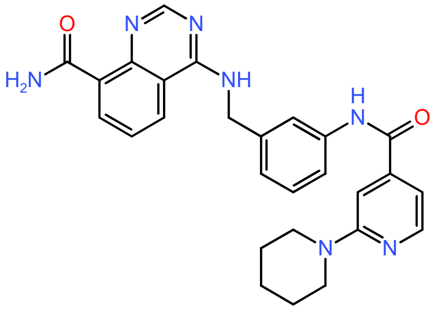

IC50(nM): 2.3
fringNplaN4B: 1
fsp3Csp2N5B: 1
N_H_2B: 7
fsp2Osp2C5B: 5
da_lipo_5B: 13
fringNC6B: 2
fOringC6B: 3

335 2013576

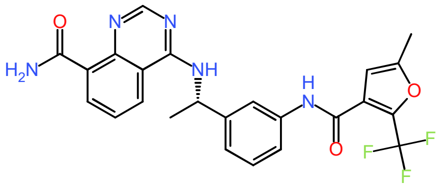

IC50(nM): 0.94
fringNplaN4B: 1
fsp3Csp2N5B: 1
N_H_2B: 5
fsp2Osp2C5B: 5
da_lipo_5B: 18
fringNC6B: 2
fOringC6B: 1

336 2013578

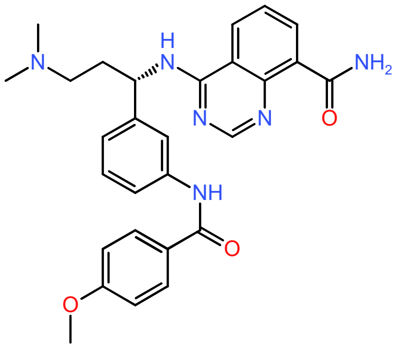

IC50(nM): 1
fringNplaN4B: 1
fsp3Csp2N5B: 1
N_H_2B: 6
fsp2Osp2C5B: 6
da_lipo_5B: 16
fringNC6B: 2
fOringC6B: 1

337 2013582

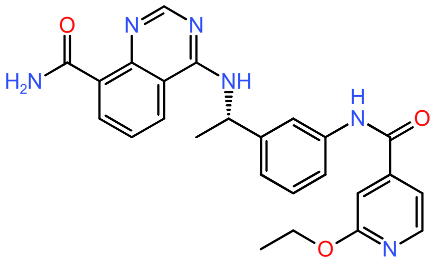

IC50(nM): 2.1
fringNplaN4B: 1
fsp3Csp2N5B: 1
N_H_2B: 6
fsp2Osp2C5B: 5
da_lipo_5B: 14
fringNC6B: 2
fOringC6B: 1

338 2013602

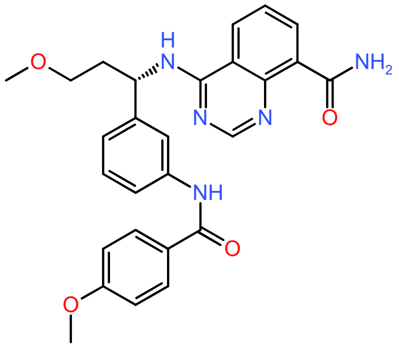

IC50(nM): 0.94
fringNplaN4B: 1
fsp3Csp2N5B: 1
N_H_2B: 5
fsp2Osp2C5B: 6
da_lipo_5B: 16
fringNC6B: 2
fOringC6B: 0

339 2013603

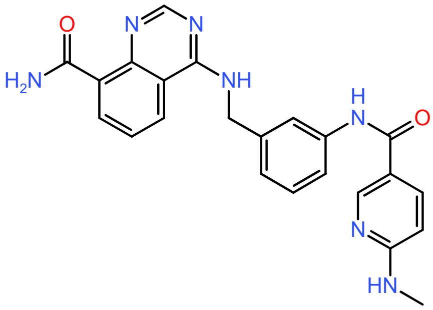

IC50(nM): 0.26
fringNplaN4B: 2
fsp3Csp2N5B: 1
N_H_2B: 7
fsp2Osp2C5B: 6
da_lipo_5B: 13
fringNC6B: 2
fOringC6B: 1

340 2013612

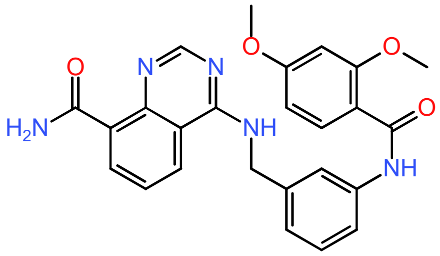

IC50(nM): 3.6
fringNplaN4B: 1
fsp3Csp2N5B: 1
N_H_2B: 5
fsp2Osp2C5B: 6
da_lipo_5B: 14
fringNC6B: 2
fOringC6B: 1

341 2013621

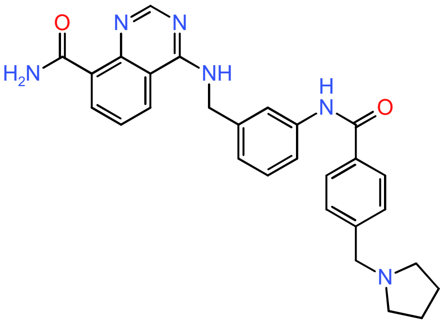

IC50(nM): 11
fringNplaN4B: 1
fsp3Csp2N5B: 1
N_H_2B: 6
fsp2Osp2C5B: 6
da_lipo_5B: 16
fringNC6B: 3
fOringC6B: 1

342 2013624

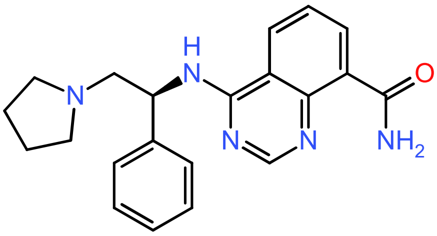

IC50(nM): 16
fringNplaN4B: 1
fsp3Csp2N5B: 1
N_H_2B: 5
fsp2Osp2C5B: 3
da_lipo_5B: 13
fringNC6B: 1
fOringC6B: 0

343 2013625

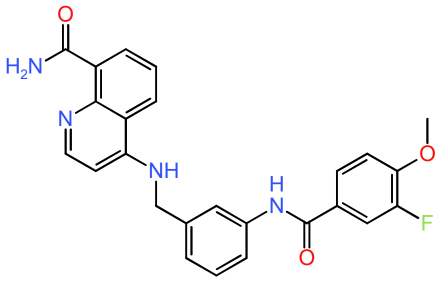

IC50(nM): 0.5
fringNplaN4B: 2
fsp3Csp2N5B: 1
N_H_2B: 4
fsp2Osp2C5B: 6
da_lipo_5B: 17
fringNC6B: 1
fOringC6B: 2

344 2013626

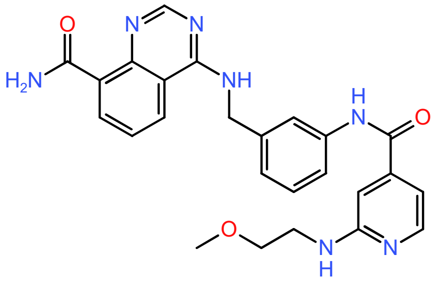

IC50(nM): 20
fringNplaN4B: 1
fsp3Csp2N5B: 1
N_H_2B: 7
fsp2Osp2C5B: 5
da_lipo_5B: 13
fringNC6B: 3
fOringC6B: 1

345 2013634

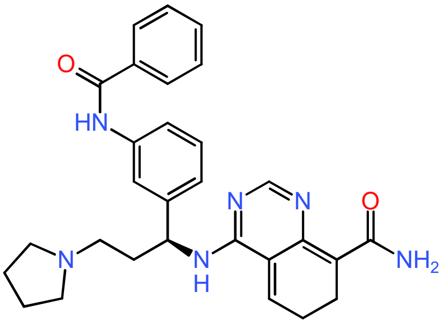

IC50(nM): 2.5
fringNplaN4B: 1
fsp3Csp2N5B: 0
N_H_2B: 6
fsp2Osp2C5B: 6
da_lipo_5B: 17
fringNC6B: 2
fOringC6B: 1

346 2013635

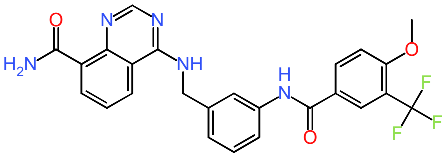

IC50(nM): 1.8
fringNplaN4B: 1
fsp3Csp2N5B: 1
N_H_2B: 5
fsp2Osp2C5B: 6
da_lipo_5B: 16
fringNC6B: 2
fOringC6B: 1

347 2013645

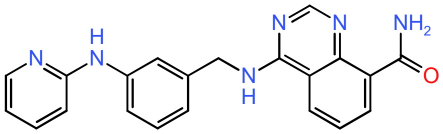

IC50(nM): 0.94
fringNplaN4B: 1
fsp3Csp2N5B: 1
N_H_2B: 6
fsp2Osp2C5B: 3
da_lipo_5B: 13
fringNC6B: 0
fOringC6B: 0

348 2013651

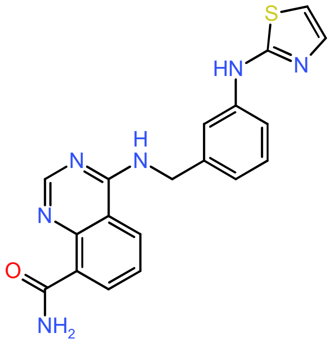

IC50(nM): 1
fringNplaN4B: 1
fsp3Csp2N5B: 1
N_H_2B: 6
fsp2Osp2C5B: 3
da_lipo_5B: 12
fringNC6B: 0
fOringC6B: 0

349 2013653

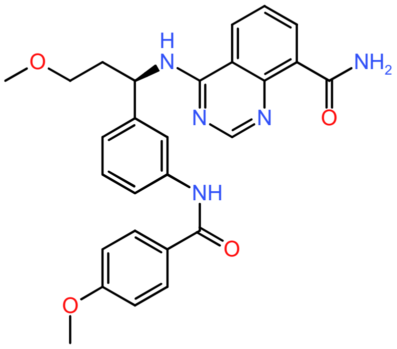

IC50(nM): 0.38
fringNplaN4B: 1
fsp3Csp2N5B: 1
N_H_2B: 5
fsp2Osp2C5B: 6
da_lipo_5B: 16
fringNC6B: 2
fOringC6B: 0

350 2013657

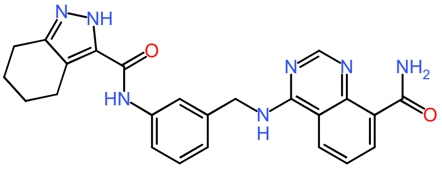

IC50(nM): 0.3
fringNplaN4B: 1
fsp3Csp2N5B: 1
N_H_2B: 7
fsp2Osp2C5B: 5
da_lipo_5B: 15
fringNC6B: 1
fOringC6B: 2

351 2013659

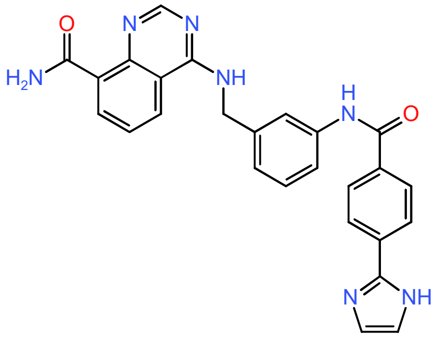

IC50(nM): 1
fringNplaN4B: 1
fsp3Csp2N5B: 1
N_H_2B: 7
fsp2Osp2C5B: 6
da_lipo_5B: 16
fringNC6B: 3
fOringC6B: 2

352 2013660

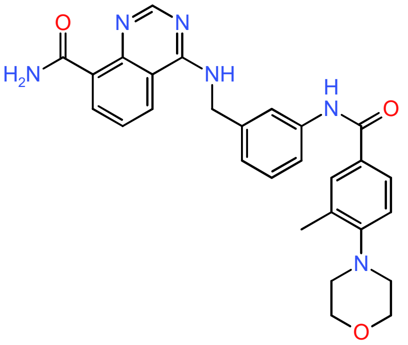

IC50(nM):2.2
fringNplaN4B:1
fsp3Csp2N5B:1
N_H_2B:6
fsp2Osp2C5B:6
da_lipo_5B:16
fringNC6B:2
fOringC6B:1

353 2013670

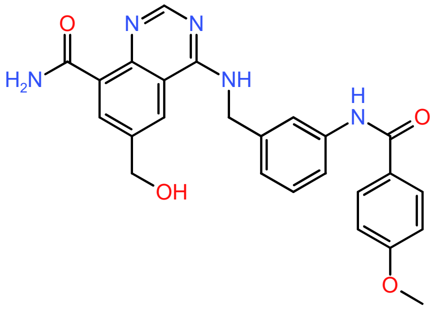

IC50(nM):0.96
fringNplaN4B:1
fsp3Csp2N5B:1
N_H_2B:5
fsp2Osp2C5B:6
da_lipo_5B:15
fringNC6B:2
fOringC6B:1

354 2013674

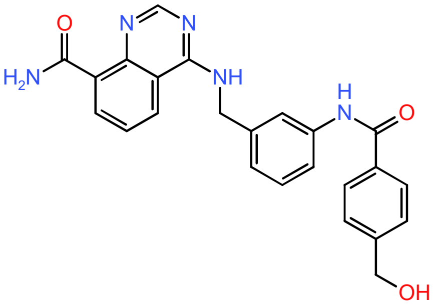

IC50(nM):0.73
fringNplaN4B:1
fsp3Csp2N5B:1
N_H_2B:5
fsp2Osp2C5B:6
da_lipo_5B:16
fringNC6B:2
fOringC6B:1

355 2013675

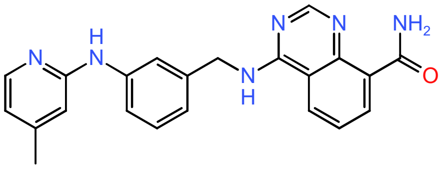

IC50(nM):0.39
fringNplaN4B:1
fsp3Csp2N5B:1
N_H_2B:6
fsp2Osp2C5B:3
da_lipo_5B:14
fringNC6B:0
fOringC6B:0

356 2013677

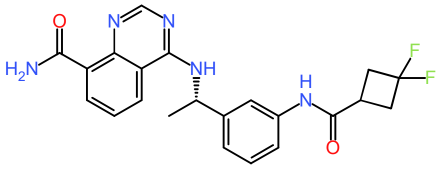

IC50(nM):6.5
fringNplaN4B:1
fsp3Csp2N5B:1
N_H_2B:5
fsp2Osp2C5B:5
da_lipo_5B:17
fringNC6B:2
fOringC6B:1

357 2013680

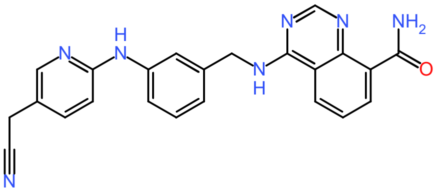

IC50(nM):0.76
fringNplaN4B:1
fsp3Csp2N5B:1
N_H_2B:6
fsp2Osp2C5B:3
da_lipo_5B:14
fringNC6B:0
fOringC6B:0

358 2013681

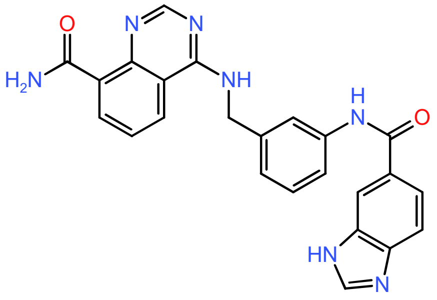

IC50(nM):4.7
fringNplaN4B:1
fsp3Csp2N5B:1
N_H_2B:7
fsp2Osp2C5B:6
da_lipo_5B:14
fringNC6B:2
fOringC6B:2

359 2013687

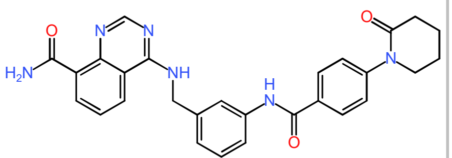

IC50(nM):17
fringNplaN4B:1
fsp3Csp2N5B:1
N_H_2B:6
fsp2Osp2C5B:5
da_lipo_5B:15
fringNC6B:2
fOringC6B:1

360 2013689

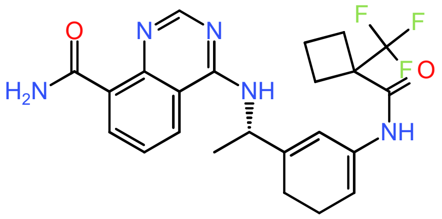

IC50(nM):9.4
fringNplaN4B:1
fsp3Csp2N5B:1
N_H_2B:5
fsp2Osp2C5B:4
da_lipo_5B:19
fringNC6B:2
fOringC6B:1

361 2013690

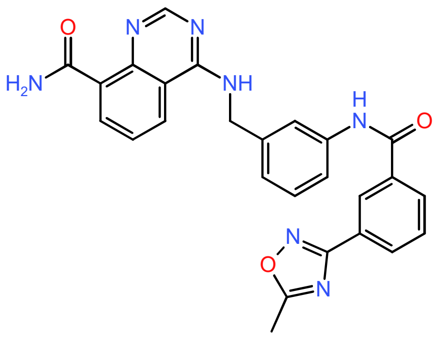

IC50(nM):0.5
fringNplaN4B:1
fsp3Csp2N5B:1
N_H_2B:5
fsp2Osp2C5B:7
da_lipo_5B:16
fringNC6B:2
fOringC6B:1

362 2013691

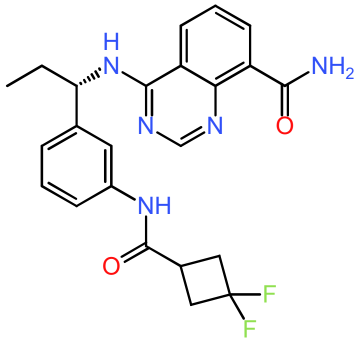

IC50(nM):0.5
fringNplaN4B:1
fsp3Csp2N5B:1
N_H_2B:5
fsp2Osp2C5B:5
da_lipo_5B:18
fringNC6B:2
fOringC6B:1

363 2013692

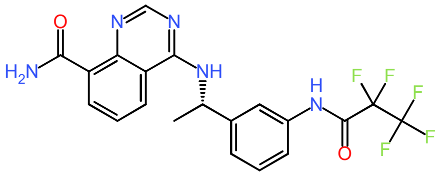

IC50(nM):15
fringNplaN4B:1
fsp3Csp2N5B:1
N_H_2B:5
fsp2Osp2C5B:5
da_lipo_5B:18
fringNC6B:2
fOringC6B:1

364 2013693

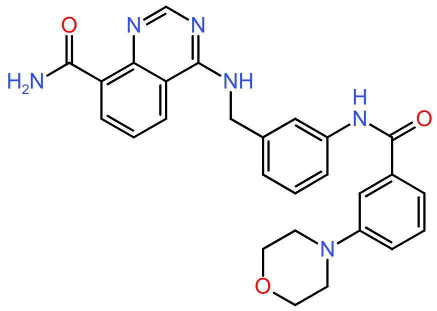

IC50(nM):5.5
fringNplaN4B:1
fsp3Csp2N5B:1
N_H_2B:6
fsp2Osp2C5B:6
da_lipo_5B:15
fringNC6B:2
fOringC6B:1

365 2013700

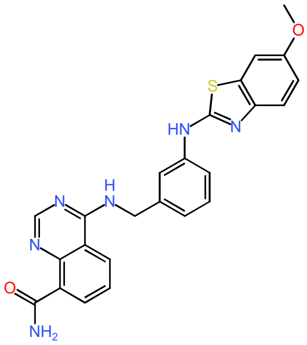

IC50(nM):16
fringNplaN4B:1
fsp3Csp2N5B:1
N_H_2B:5
fsp2Osp2C5B:3
da_lipo_5B:15
fringNC6B:1
fOringC6B:0

366 2013701

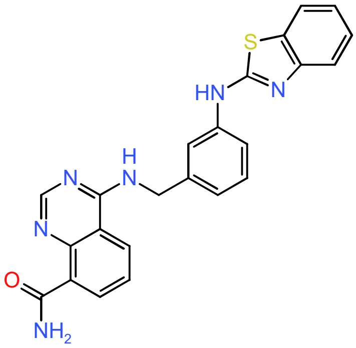

IC50(nM):2.5
fringNplaN4B:1
fsp3Csp2N5B:1
N_H_2B:5
fsp2Osp2C5B:3
da_lipo_5B:16
fringNC6B:0
fOringC6B:0

367 2013703

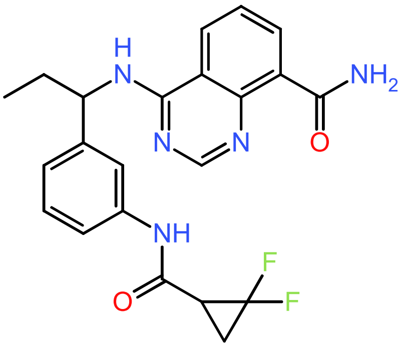

IC50(nM):0.54
fringNplaN4B:1
fsp3Csp2N5B:1
N_H_2B:5
fsp2Osp2C5B:5
da_lipo_5B:17
fringNC6B:2
fOringC6B:1

368 2013705

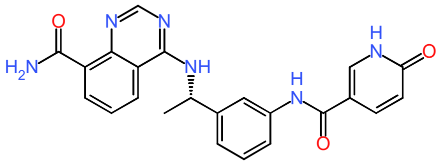

IC50(nM):6.8
fringNplaN4B:2
fsp3Csp2N5B:1
N_H_2B:6
fsp2Osp2C5B:5
da_lipo_5B:14
fringNC6B:2
fOringC6B:1

369 2013706

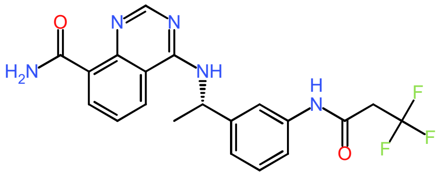

IC50(nM):4.2
fringNplaN4B:1
fsp3Csp2N5B:1
N_H_2B:5
fsp2Osp2C5B:5
da_lipo_5B:16
fringNC6B:2
fOringC6B:1

370 2013709

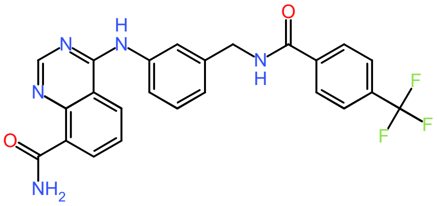

IC50(nM):4.7
fringNplaN4B:1
fsp3Csp2N5B:0
N_H_2B:5
fsp2Osp2C5B:6
da_lipo_5B: 16
fringNC6B:2
fOringC6B:2

371 2018350

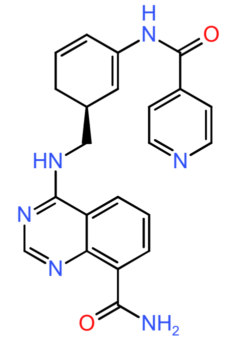

IC50(nM):8.9
fringNplaN4B:1
fsp3Csp2N5B:1
N_H_2B:6
fsp2Osp2C5B:4
da_lipo_5B: 13
fringNC6B:2
fOringC6B:1

372 2018352

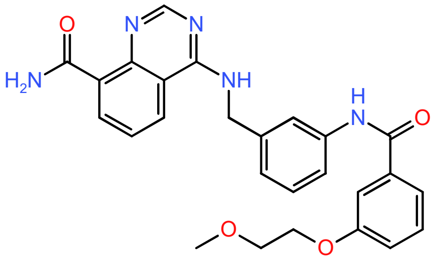

IC50(nM):18
fringNplaN4B:1
fsp3Csp2N5B:1
N_H_2B:5
fsp2Osp2C5B:6
da_lipo_5B: 15
fringNC6B:2
fOringC6B:1

373 2018360

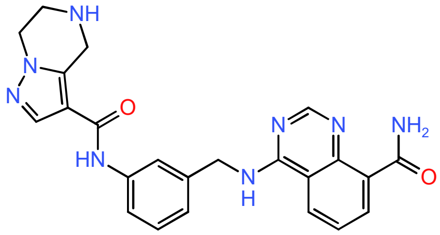

IC50(nM):1.6
fringNplaN4B:2
fsp3Csp2N5B:1
N_H_2B:8
fsp2Osp2C5B:5
da_lipo_5B: 11
fringNC6B:2
fOringC6B:2

374 2018361

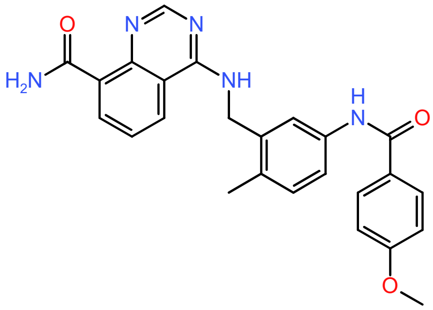

IC50(nM):32
fringNplaN4B:1
fsp3Csp2N5B:1
N_H_2B:5
fsp2Osp2C5B:6
da_lipo_5B: 16
fringNC6B:3
fOringC6B:1

375 2018362

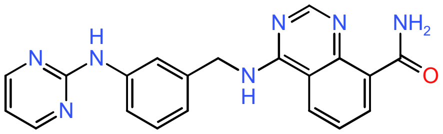

IC50(nM):3.3
fringNplaN4B:1
fsp3Csp2N5B:1
N_H_2B:7
fsp2Osp2C5B:3
da_lipo_5B: 11
fringNC6B:0
fOringC6B:0

376 2018364

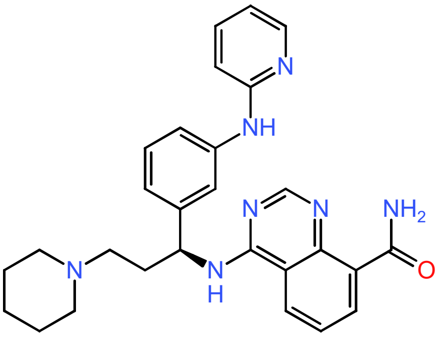

IC50(nM):1.5
fringNplaN4B:1
fsp3Csp2N5B:1
N_H_2B:7
fsp2Osp2C5B:3
da_lipo_5B: 14
fringNC6B:0
fOringC6B:0

377 2018365

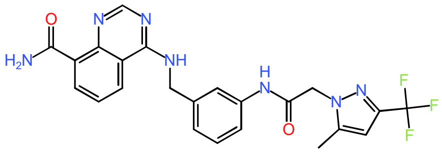

IC50(nM):4.4
fringNplaN4B:1
fsp3Csp2N5B:1
N_H_2B:6
fsp2Osp2C5B:7
da_lipo_5B: 12
fringNC6B:1
fOringC6B:1

378 2018366

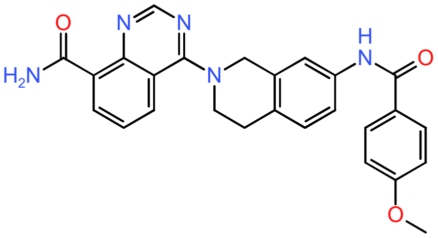

IC50(nM):20
fringNplaN4B:1
fsp3Csp2N5B:1
N_H_2B:5
fsp2Osp2C5B:6
da_lipo_5B: 16
fringNC6B:1
fOringC6B:2

379 2018367

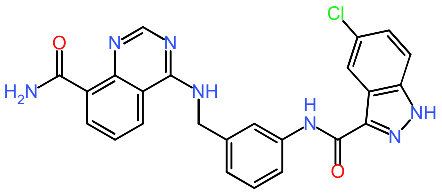

IC50(nM):5
fringNplaN4B:1
fsp3Csp2N5B:1
N_H_2B:7
fsp2Osp2C5B:7
da_lipo_5B: 16
fringNC6B:1
fOringC6B:2

380 2018371

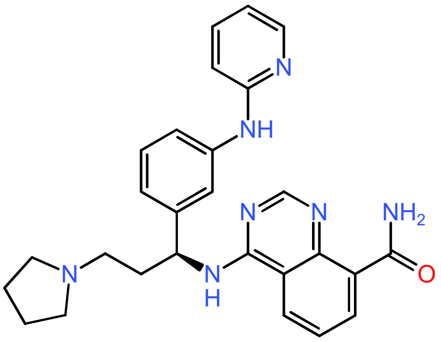

IC50(nM):5
fringNplaN4B:1
fsp3Csp2N5B:1
N_H_2B:7
fsp2Osp2C5B:3
da_lipo_5B: 14
fringNC6B:0
fOringC6B:0

381 2018372

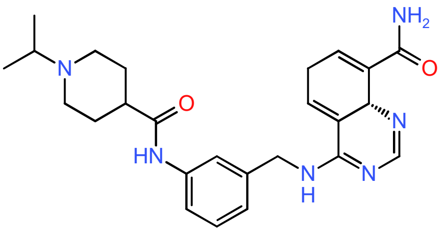

IC50(nM):1.9
fringNplaN4B:1
fsp3Csp2N5B:0
N_H_2B:6
fsp2Osp2C5B:5
da_lipo_5B: 13
fringNC6B:2
fOringC6B:1

382 2018382

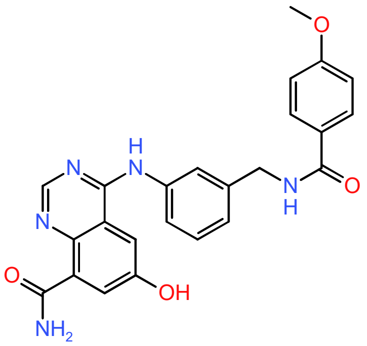

IC50(nM):8.7
fringNplaN4B:1
fsp3Csp2N5B:0
N_H_2B:5
fsp2Osp2C5B:6
da_lipo_5B: 14
fringNC6B:2
fOringC6B:2

383 2018387

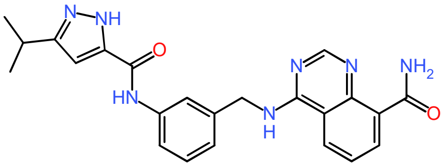

IC50(nM):0.33
fringNplaN4B:1
fsp3Csp2N5B:1
N_H_2B:7
fsp2Osp2C5B:5
da_lipo_5B: 14
fringNC6B:1
fOringC6B:1

384 2018388

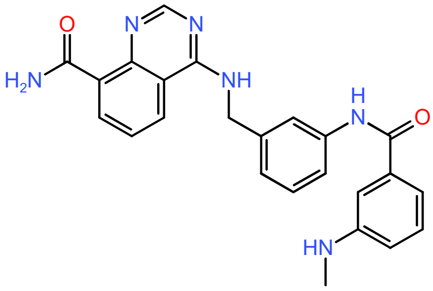

IC50(nM):15
fringNplaN4B:1
fsp3Csp2N5B:1
N_H_2B:6
fsp2Osp2C5B:6
da_lipo_5B: 15
fringNC6B:2
fOringC6B:1

385 2018390

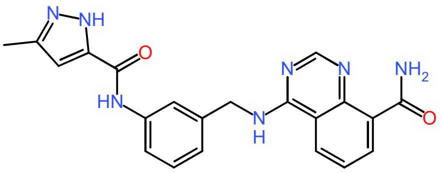

IC50(nM):2.3
fringNplaN4B:1
fsp3Csp2N5B:1
N_H_2B:7
fsp2Osp2C5B:5
da_lipo_5B: 12
fringNC6B:1
fOringC6B:1

386 2018391

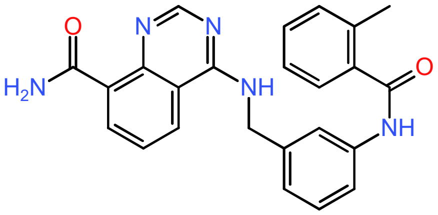

IC50(nM):1.2
fringNplaN4B:1
fsp3Csp2N5B:1
N_H_2B:5
fsp2Osp2C5B:6
da_lipo_5B: 17
fringNC6B:2
fOringC6B:1

387 2018392

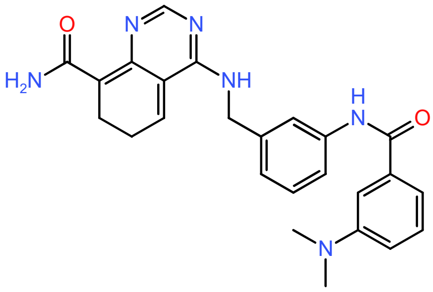

IC50(nM):1.7
fringNplaN4B:1
fsp3Csp2N5B:0
N_H_2B:6
fsp2Osp2C5B:6
da_lipo_5B: 15
fringNC6B:2
fOringC6B:1

388 2018396

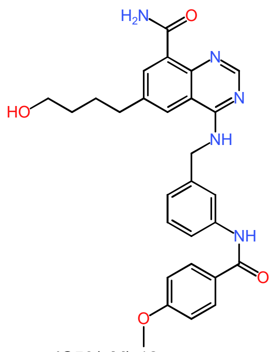

IC50(nM): 12
fringNplaN4B: 1
fsp3Csp2N5B: 1
N_H_2B: 5
fsp2Osp2C5B: 6
da_lipo_5B: 18
fringNC6B: 3
fOringC6B: 1

389 2018397

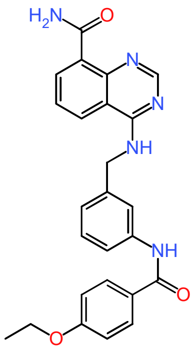

IC50(nM): 1.8
fringNplaN4B: 1
fsp3Csp2N5B: 1
N_H_2B: 5
fsp2Osp2C5B: 6
da_lipo_5B: 15
fringNC6B: 2
fOringC6B: 1

390 2018398

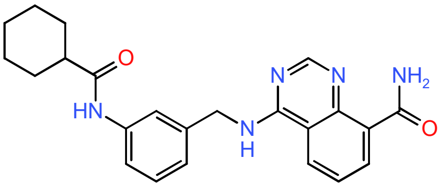

IC50(nM): 1.1
fringNplaN4B: 1
fsp3Csp2N5B: 1
N_H_2B: 5
fsp2Osp2C5B: 5
da_lipo_5B: 16
fringNC6B: 2
fOringC6B: 1

391 2018399

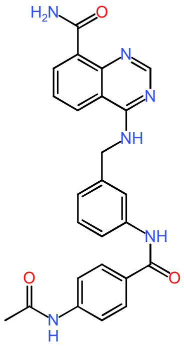

IC50(nM): 0.5
fringNplaN4B: 1
fsp3Csp2N5B: 1
N_H_2B: 6
fsp2Osp2C5B: 5
da_lipo_5B: 16
fringNC6B: 2
fOringC6B: 1

392 2018401

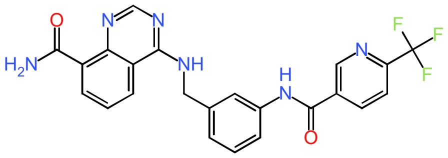

IC50(nM): 0.38
fringNplaN4B: 2
fsp3Csp2N5B: 1
N_H_2B: 6
fsp2Osp2C5B: 6
da_lipo_5B: 13
fringNC6B: 2
fOringC6B: 1

393 2018402

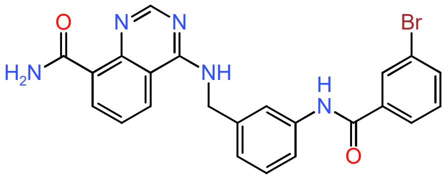

IC50(nM): 0.79
fringNplaN4B: 1
fsp3Csp2N5B: 1
N_H_2B: 5
fsp2Osp2C5B: 6
da_lipo_5B: 17
fringNC6B: 2
fOringC6B: 1

394 2018403

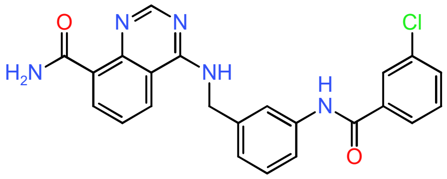

IC50(nM): 0.38
fringNplaN4B: 1
fsp3Csp2N5B: 1
N_H_2B: 5
fsp2Osp2C5B: 6
da_lipo_5B: 17
fringNC6B: 2
fOringC6B: 1

395 2018404

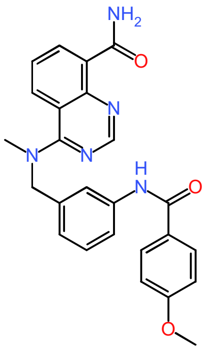

IC50(nM): 13
fringNplaN4B: 1
fsp3Csp2N5B: 1
N_H_2B: 5
fsp2Osp2C5B: 6
da_lipo_5B: 15
fringNC6B: 2
fOringC6B: 1

396 2018405

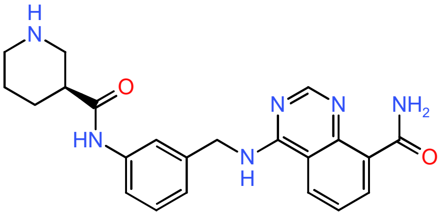

IC50(nM): 1.6
fringNplaN4B: 2
fsp3Csp2N5B: 1
N_H_2B: 6
fsp2Osp2C5B: 5
da_lipo_5B: 13
fringNC6B: 2
fOringC6B: 1

397 2018406

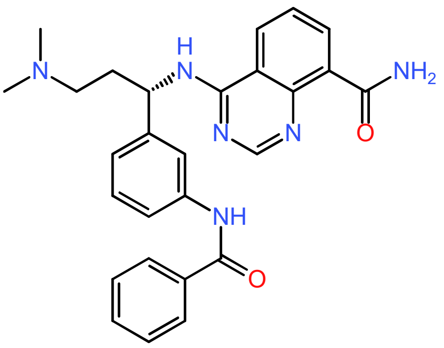

IC50(nM): 1.9
fringNplaN4B: 1
fsp3Csp2N5B: 1
N_H_2B: 6
fsp2Osp2C5B: 6
da_lipo_5B: 17
fringNC6B: 2
fOringC6B: 1

398 2018407

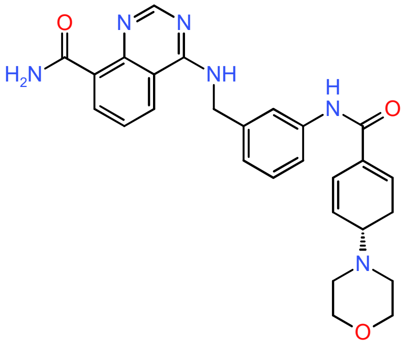

IC50(nM): 6.9
fringNplaN4B: 1
fsp3Csp2N5B: 1
N_H_2B: 6
fsp2Osp2C5B: 5
da_lipo_5B: 15
fringNC6B: 2
fOringC6B: 1

399 2018409

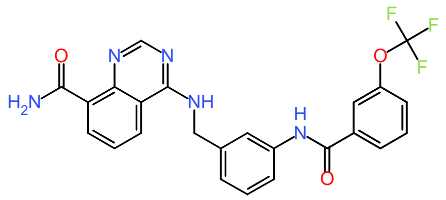

IC50(nM): 2
fringNplaN4B: 1
fsp3Csp2N5B: 1
N_H_2B: 5
fsp2Osp2C5B: 6
da_lipo_5B: 15
fringNC6B: 2
fOringC6B: 1

400 2018410

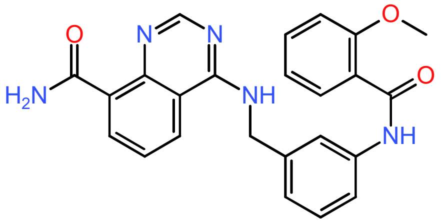

IC50(nM): 4.6
fringNplaN4B: 1
fsp3Csp2N5B: 1
N_H_2B: 5
fsp2Osp2C5B: 6
da_lipo_5B: 15
fringNC6B: 2
fOringC6B: 1

401 2018412

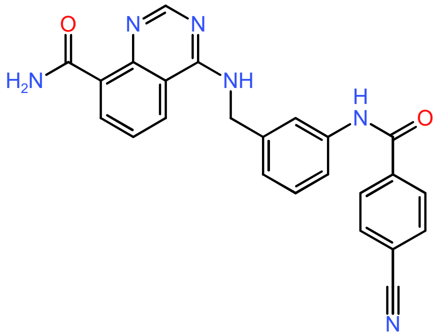

IC50(nM): 2.4
fringNplaN4B: 1
fsp3Csp2N5B: 1
N_H_2B: 5
fsp2Osp2C5B: 6
da_lipo_5B: 16
fringNC6B: 2
fOringC6B: 1

402 2018414

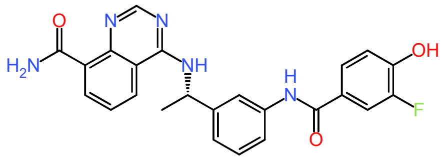

IC50(nM): 14
fringNplaN4B: 1
fsp3Csp2N5B: 1
N_H_2B: 5
fsp2Osp2C5B: 6
da_lipo_5B: 17
fringNC6B: 2
fOringC6B: 1

403 2018415

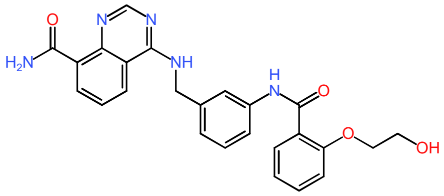

IC50(nM): 16
fringNplaN4B: 1
fsp3Csp2N5B: 1
N_H_2B: 5
fsp2Osp2C5B: 6
da_lipo_5B: 15
fringNC6B: 2
fOringC6B: 1

404 2018416

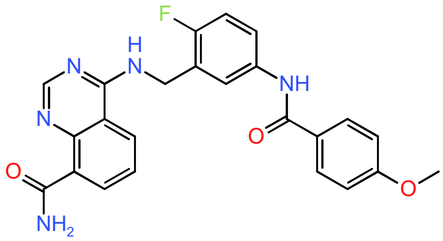

IC50(nM): 2.7
fringNplaN4B: 1
fsp3Csp2N5B: 1
N_H_2B: 5
fsp2Osp2C5B: 6
da_lipo_5B: 16
fringNC6B: 2
fOringC6B: 1

405 2018417

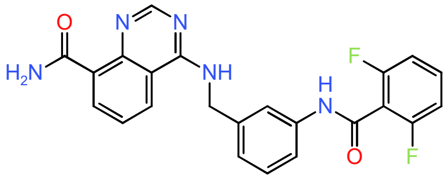

IC50(nM): 0.88
fringNplaN4B: 1
fsp3Csp2N5B: 1
N_H_2B: 5
fsp2Osp2C5B: 6
da_lipo_5B: 18
fringNC6B: 2
fOringC6B: 1

406 2018418

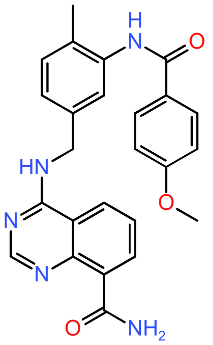

IC50(nM):0.8
fringNplaN4B:1
fsp3Csp2N5B:1
N_H_2B:5
fsp2Osp2C5B:6
da_lipo_5B:16
fringNC6B:2
fOringC6B:1

407 2018419

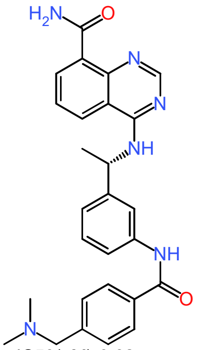

IC50(nM):0.62
fringNplaN4B:1
fsp3Csp2N5B:1
N_H_2B:6
fsp2Osp2C5B:6
da_lipo_5B:17
fringNC6B:2
fOringC6B:1

408 2018423

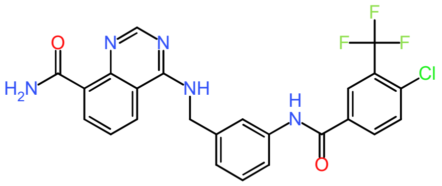

IC50(nM):0.52
fringNplaN4B:1
fsp3Csp2N5B:1
N_H_2B:5
fsp2Osp2C5B:6
da_lipo_5B:17
fringNC6B:2
fOringC6B:1

409 2018425

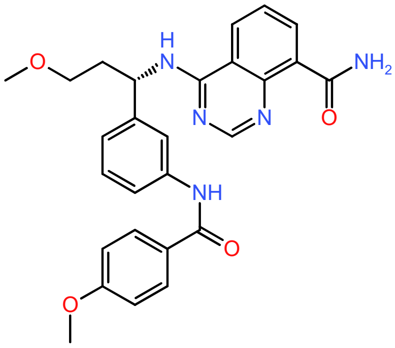

IC50(nM):7.6
fringNplaN4B:1
fsp3Csp2N5B:1
N_H_2B:5
fsp2Osp2C5B:6
da_lipo_5B:16
fringNC6B:2
fOringC6B:0

410 2018430

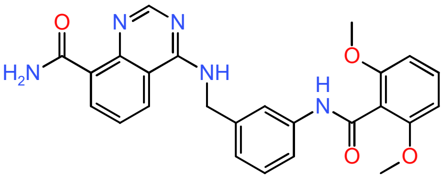

IC50(nM):9.8
fringNplaN4B:1
fsp3Csp2N5B:1
N_H_2B:5
fsp2Osp2C5B:6
da_lipo_5B:14
fringNC6B:2
fOringC6B:1

411 2018431

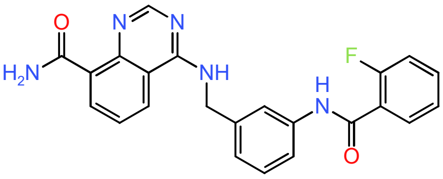

IC50(nM):2
fringNplaN4B:1
fsp3Csp2N5B:1
N_H_2B:5
fsp2Osp2C5B:6
da_lipo_5B:17
fringNC6B:2
fOringC6B:1

412 2018436

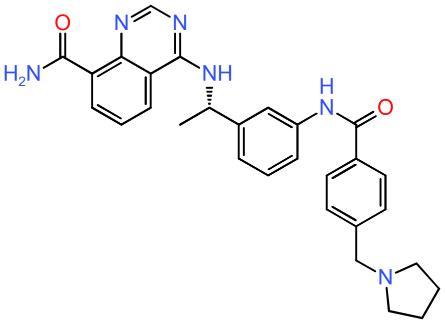

IC50(nM):3.3
fringNplaN4B:1
fsp3Csp2N5B:1
N_H_2B:6
fsp2Osp2C5B:6
da_lipo_5B:17
fringNC6B:3
fOringC6B:1

413 2018438

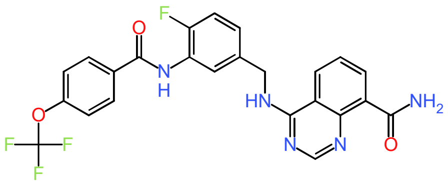

IC50(nM):1.8
fringNplaN4B:1
fsp3Csp2N5B:1
N_H_2B:5
fsp2Osp2C5B:6
da_lipo_5B:16
fringNC6B:2
fOringC6B:1

414 2018444

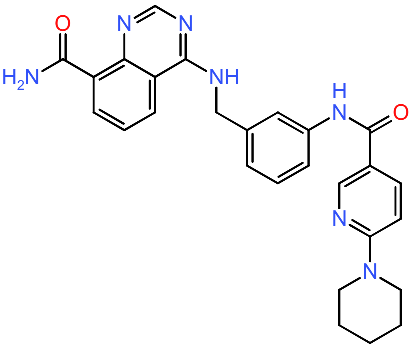

IC50(nM):0.81
fringNplaN4B:2
fsp3Csp2N5B:1
N_H_2B:7
fsp2Osp2C5B:6
da_lipo_5B:13
fringNC6B:2
fOringC6B:1

415 2018460

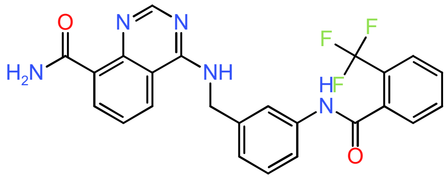

IC50(nM): 3.8
fringNplaN4B: 1
fsp3Csp2N5B: 1
N_H_2B: 5
fsp2Osp2C5B: 6
da_lipo_5B: 20
fringNC6B: 2
fOringC6B: 1

416 2018468

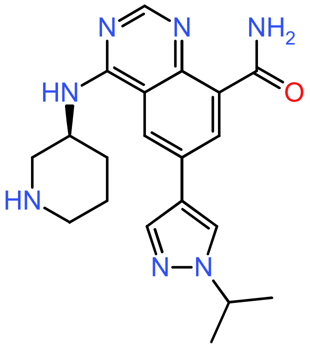

IC50(nM): 18
fringNplaN4B: 1
fsp3Csp2N5B: 1
N_H_2B: 7
fsp2Osp2C5B: 4
da_lipo_5B: 8
fringNC6B: 0
fOringC6B: 2

417 2025431

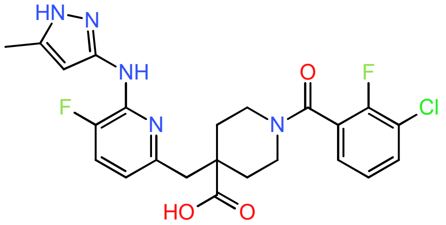

IC50(nM): 180
fringNplaN4B: 0
fsp3Csp2N5B: 0
N_H_2B: 4
fsp2Osp2C5B: 1
da_lipo_5B: 10
fringNC6B: 0
fOringC6B: 2

418 2038679

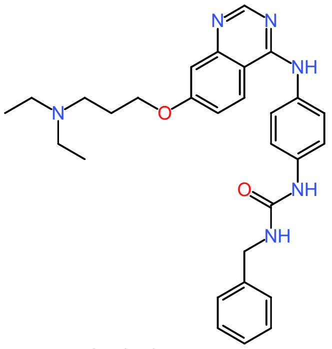

IC50(nM): 196
fringNplaN4B: 0
fsp3Csp2N5B: 1
N_H_2B: 6
fsp2Osp2C5B: 4
da_lipo_5B: 14
fringNC6B: 2
fOringC6B: 3

419 2038680

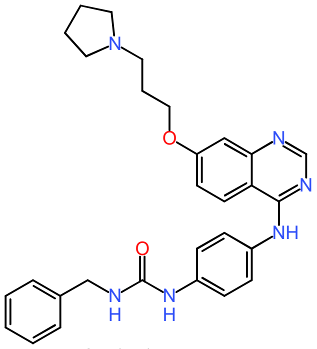

IC50(nM): 278
fringNplaN4B: 0
fsp3Csp2N5B: 1
N_H_2B: 6
fsp2Osp2C5B: 4
da_lipo_5B: 14
fringNC6B: 1
fOringC6B: 5

420 2038689

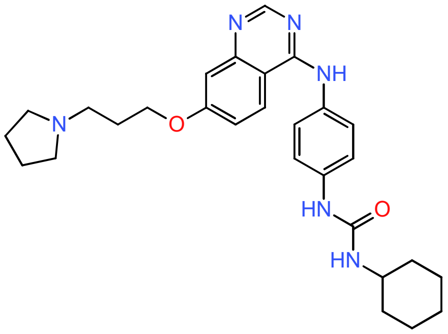

IC50(nM): 181
fringNplaN4B: 0
fsp3Csp2N5B: 1
N_H_2B: 6
fsp2Osp2C5B: 2
da_lipo_5B: 13
fringNC6B: 1
fOringC6B: 4

421 2038690

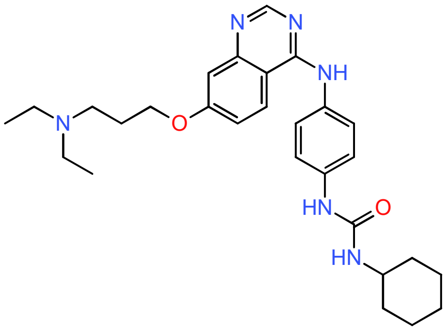

IC50(nM): 176
fringNplaN4B: 0
fsp3Csp2N5B: 1
N_H_2B: 6
fsp2Osp2C5B: 2
da_lipo_5B: 13
fringNC6B: 2
fOringC6B: 2

422 2056426

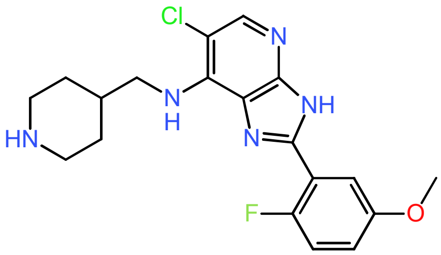

IC50(nM): 157
fringNplaN4B: 0
fsp3Csp2N5B: 1
N_H_2B: 4
fsp2Osp2C5B: 0
da_lipo_5B: 11
fringNC6B: 1
fOringC6B: 2

423 2056428

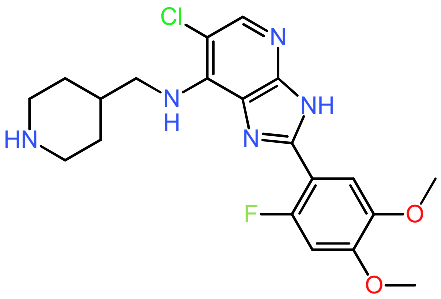

IC50(nM): 132
fringNplaN4B: 0
fsp3Csp2N5B: 1
N_H_2B: 4
fsp2Osp2C5B: 0
da_lipo_5B: 10
fringNC6B: 1
fOringC6B: 2

424 2079522

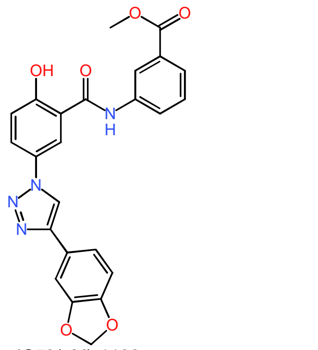

IC50(nM): 1108
fringNplaN4B: 0
fsp3Csp2N5B: 0
N_H_2B: 2
fsp2Osp2C5B: 3
da_lipo_5B: 9
fringNC6B: 2
fOringC6B: 0

425 2097650

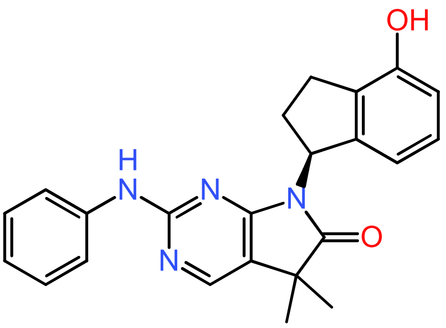

IC50(nM): 163
fringNplaN4B: 0
fsp3Csp2N5B: 0
N_H_2B: 3
fsp2Osp2C5B: 3
da_lipo_5B: 14
fringNC6B: 1
fOringC6B: 0

426 2127354

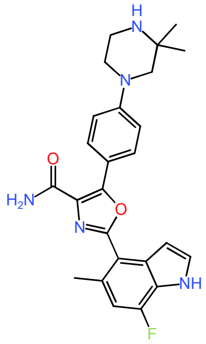

IC50(nM): 5.93
fringNplaN4B: 0
fsp3Csp2N5B: 0
N_H_2B: 4
fsp2Osp2C5B: 3
da_lipo_5B: 15
fringNC6B: 0
fOringC6B: 0

427 2130617

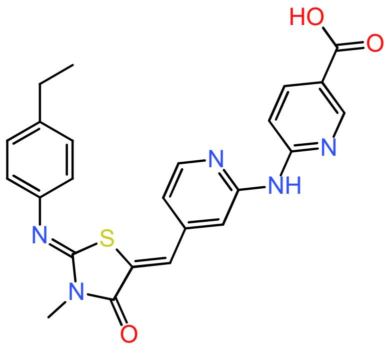

IC50(nM): 12505.05
fringNplaN4B: 0
fsp3Csp2N5B: 0
N_H_2B: 4
fsp2Osp2C5B: 3
da_lipo_5B: 8
fringNC6B: 1
fOringC6B: 4

428 2131546

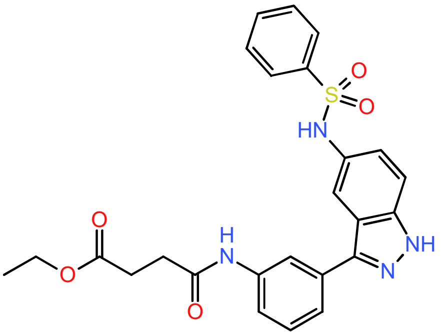

IC50(nM): 64
fringNplaN4B: 0
fsp3Csp2N5B: 0
N_H_2B: 4
fsp2Osp2C5B: 5
da_lipo_5B: 17
fringNC6B: 1
fOringC6B: 3

429 2132789

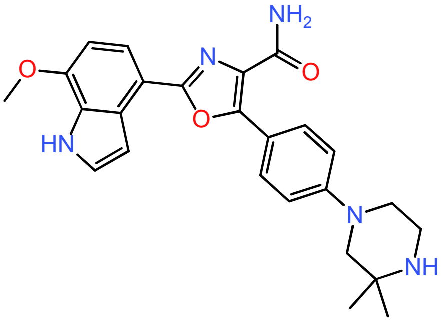

IC50(nM): 8.78
fringNplaN4B: 0
fsp3Csp2N5B: 0
N_H_2B: 4
fsp2Osp2C5B: 3
da_lipo_5B: 12
fringNC6B: 0
fOringC6B: 0

430 2138320

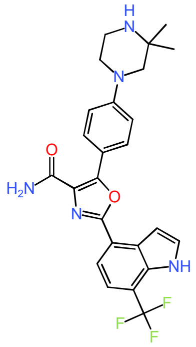

IC50(nM): 14
fringNplaN4B: 0
fsp3Csp2N5B: 0
N_H_2B: 4
fsp2Osp2C5B: 3
da_lipo_5B: 17
fringNC6B: 0
fOringC6B: 0

431 2141733

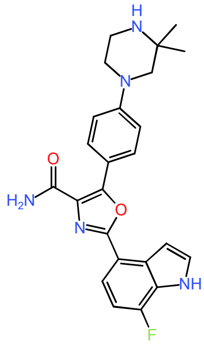

IC50(nM): 9.36
fringNplaN4B: 0
fsp3Csp2N5B: 0
N_H_2B: 4
fsp2Osp2C5B: 3
da_lipo_5B: 14
fringNC6B: 0
fOringC6B: 0

432 2148736

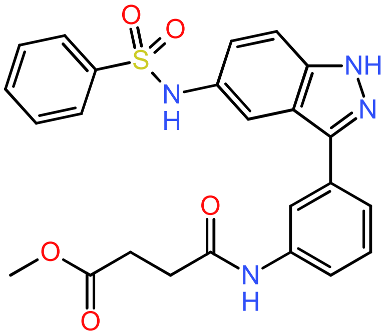

IC50(nM): 48
fringNplaN4B: 0
fsp3Csp2N5B: 0
N_H_2B: 4
fsp2Osp2C5B: 5
da_lipo_5B: 17
fringNC6B: 1
fOringC6B: 3

433 2150230

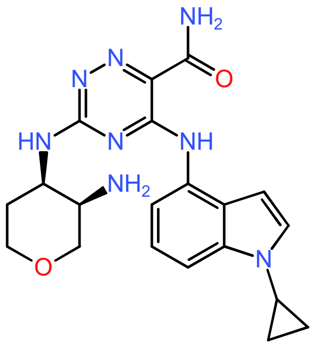

IC50(nM): 1300
fringNplaN4B: 0
fsp3Csp2N5B: 0
N_H_2B: 5
fsp2Osp2C5B: 2
da_lipo_5B: 6
fringNC6B: 0
fOringC6B: 2

434 2154196

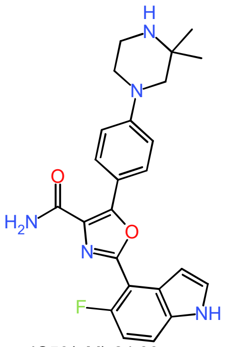

IC50(nM): 24.33
fringNplaN4B: 0
fsp3Csp2N5B: 0
N_H_2B: 4
fsp2Osp2C5B: 3
da_lipo_5B: 14
fringNC6B: 0
fOringC6B: 0

435 2155241

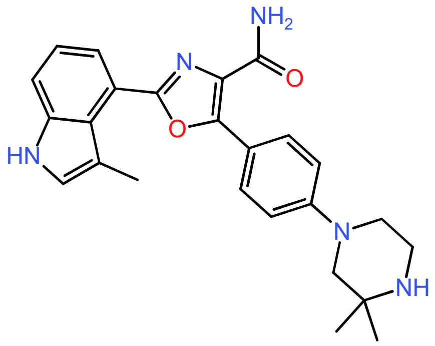

IC50(nM): 14.2
fringNplaN4B: 0
fsp3Csp2N5B: 1
N_H_2B: 4
fsp2Osp2C5B: 3
da_lipo_5B: 14
fringNC6B: 0
fOringC6B: 0

436 2163348

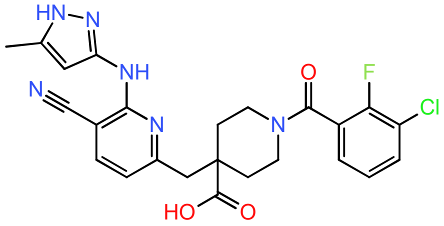

IC50(nM): 320
fringNplaN4B: 0
fsp3Csp2N5B: 0
N_H_2B: 4
fsp2Osp2C5B: 1
da_lipo_5B: 9
fringNC6B: 0
fOringC6B: 2

437 2163941

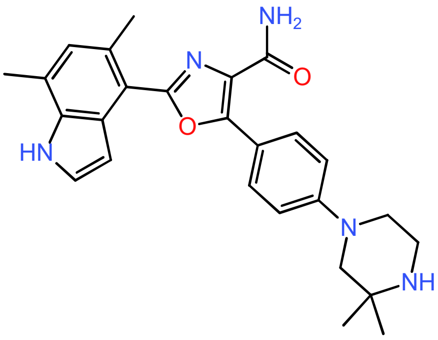

IC50(nM): 19.62
fringNplaN4B: 0
fsp3Csp2N5B: 0
N_H_2B: 4
fsp2Osp2C5B: 3
da_lipo_5B: 15
fringNC6B: 0
fOringC6B: 0

438 2165875

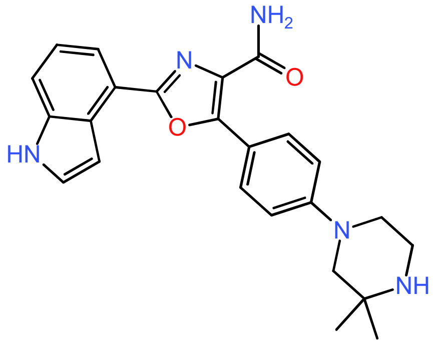

IC50(nM): 15.21
fringNplaN4B: 0
fsp3Csp2N5B: 0
N_H_2B: 4
fsp2Osp2C5B: 3
da_lipo_5B: 13
fringNC6B: 0
fOringC6B: 0

439 2167240

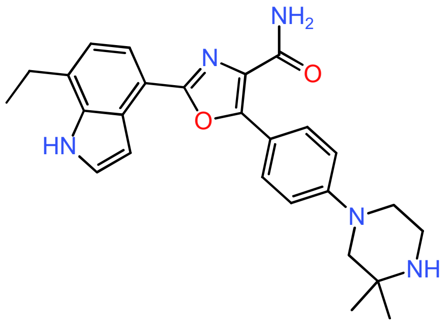

IC50(nM): 12
fringNplaN4B: 0
fsp3Csp2N5B: 0
N_H_2B: 4
fsp2Osp2C5B: 3
da_lipo_5B: 15
fringNC6B: 0
fOringC6B: 0

440 2169237

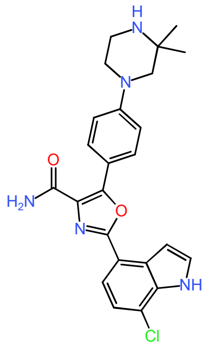

IC50(nM): 13.87
fringNplaN4B: 0
fsp3Csp2N5B: 0
N_H_2B: 4
fsp2Osp2C5B: 3
da_lipo_5B: 14
fringNC6B: 0
fOringC6B: 0

441 2180292

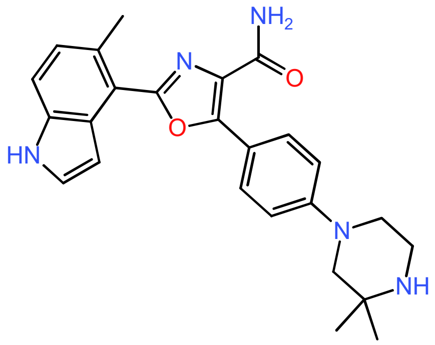

IC50(nM): 5.9
fringNplaN4B: 0
fsp3Csp2N5B: 0
N_H_2B: 4
fsp2Osp2C5B: 3
da_lipo_5B: 14
fringNC6B: 0
fOringC6B: 0

442 2183746

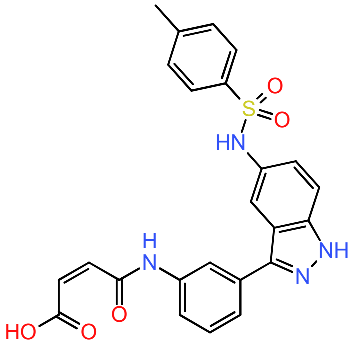

IC50(nM): 32
fringNplaN4B: 0
fsp3Csp2N5B: 0
N_H_2B: 4
fsp2Osp2C5B: 5
da_lipo_5B: 17
fringNC6B: 1
fOringC6B: 3

443 2184713

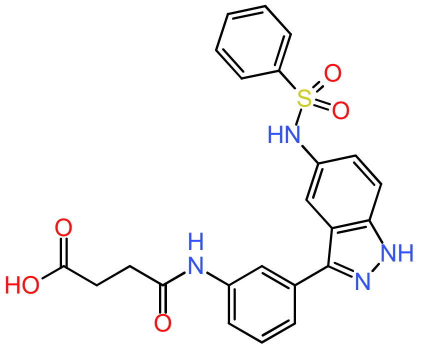

IC50(nM): 28
fringNplaN4B: 0
fsp3Csp2N5B: 0
N_H_2B: 4
fsp2Osp2C5B: 5
da_lipo_5B: 17
fringNC6B: 1
fOringC6B: 3

444 2192698

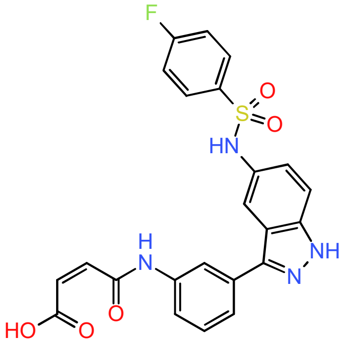

IC50(nM): 30
fringNplaN4B: 0
fsp3Csp2N5B: 0
N_H_2B: 4
fsp2Osp2C5B: 5
da_lipo_5B: 17
fringNC6B: 1
fOringC6B: 3

445 2195980

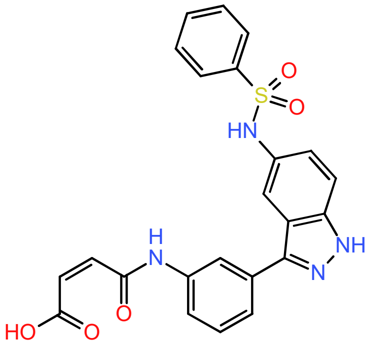

IC50(nM): 52
fringNplaN4B: 0
fsp3Csp2N5B: 0
N_H_2B: 4
fsp2Osp2C5B: 5
da_lipo_5B: 17
fringNC6B: 1
fOringC6B: 3

446 2228127

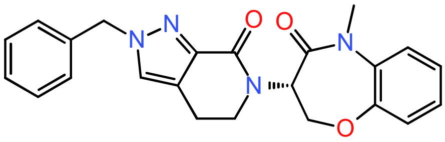

IC50(nM): 1000
fringNplaN4B: 0
fsp3Csp2N5B: 0
N_H_2B: 3
fsp2Osp2C5B: 2
da_lipo_5B: 0
fringNC6B: 0
fOringC6B: 1

447 2230634

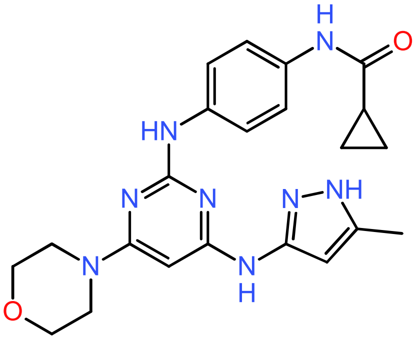

IC50(nM): 853
fringNplaN4B: 0
fsp3Csp2N5B: 1
N_H_2B: 6
fsp2Osp2C5B: 2
da_lipo_5B: 10
fringNC6B: 1
fOringC6B: 3

448 2237689

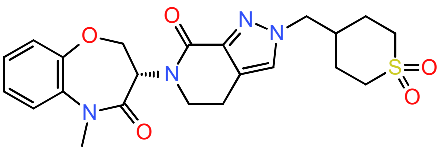

IC50(nM): 1000
fringNplaN4B: 0
fsp3Csp2N5B: 0
N_H_2B: 3
fsp2Osp2C5B: 2
da_lipo_5B: 0
fringNC6B: 0
fOringC6B: 0

449 2238403

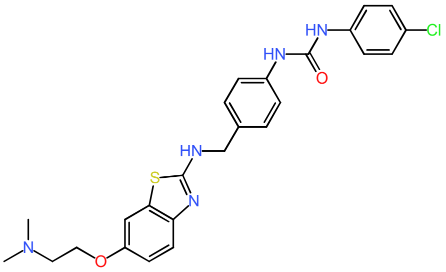

IC50(nM): 192.5
fringNplaN4B: 0
fsp3Csp2N5B: 0
N_H_2B: 4
fsp2Osp2C5B: 4
da_lipo_5B: 16
fringNC6B: 3
fOringC6B: 2

450 2254147

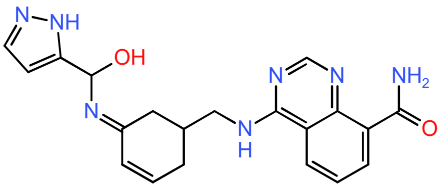

IC50(nM): 5
fringNplaN4B: 1
fsp3Csp2N5B: 1
N_H_2B: 7
fsp2Osp2C5B: 3
da_lipo_5B: 11
fringNC6B: 1
fOringC6B: 1

451 2254163

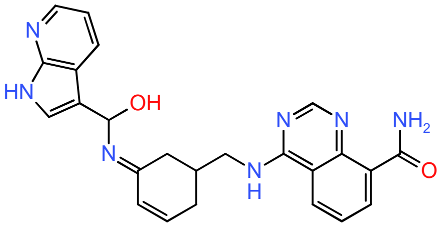

IC50(nM): 13
fringNplaN4B: 1
fsp3Csp2N5B: 1
N_H_2B: 7
fsp2Osp2C5B: 3
da_lipo_5B: 14
fringNC6B: 2
fOringC6B: 2

452 2262770

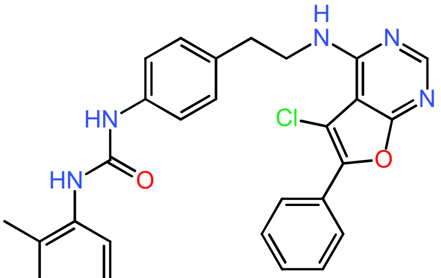

IC50(nM): 60.2
fringNplaN4B: 0
fsp3Csp2N5B: 1
N_H_2B: 5
fsp2Osp2C5B: 4
da_lipo_5B: 16
fringNC6B: 4
fOringC6B: 2

453 2263291

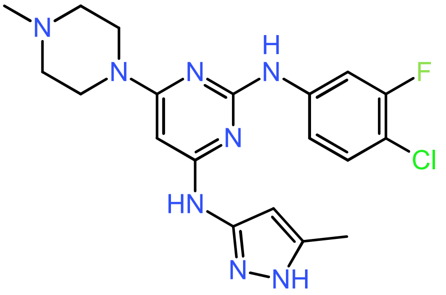

IC50(nM): 101
fringNplaN4B: 0
fsp3Csp2N5B: 1
N_H_2B: 6
fsp2Osp2C5B: 0
da_lipo_5B: 10
fringNC6B: 1
fOringC6B: 0

454 2265664

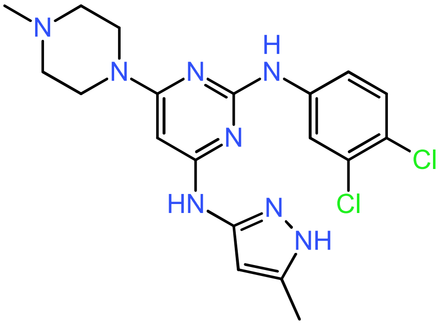

IC50(nM): 50
fringNplaN4B: 0
fsp3Csp2N5B: 1
N_H_2B: 6
fsp2Osp2C5B: 0
da_lipo_5B: 10
fringNC6B: 1
fOringC6B: 0

455 2266562

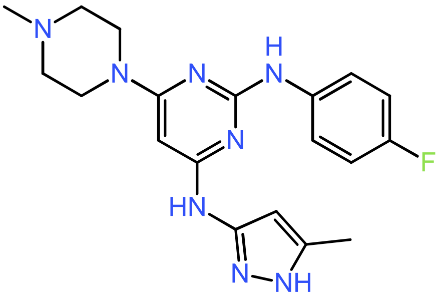

IC50(nM): 75.1
fringNplaN4B: 0
fsp3Csp2N5B: 1
N_H_2B: 6
fsp2Osp2C5B: 0
da_lipo_5B: 9
fringNC6B: 1
fOringC6B: 0

456 2267767

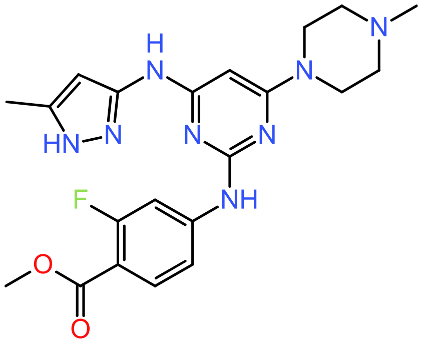

IC50(nM): 145
fringNplaN4B: 0
fsp3Csp2N5B: 1
N_H_2B: 6
fsp2Osp2C5B: 0
da_lipo_5B: 9
fringNC6B: 1
fOringC6B: 0

457 2268090

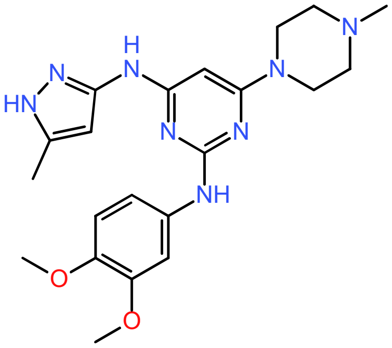

IC50(nM): 452
fringNplaN4B: 0
fsp3Csp2N5B: 1
N_H_2B: 6
fsp2Osp2C5B: 0
da_lipo_5B: 6
fringNC6B: 1
fOringC6B: 0

458 2269322

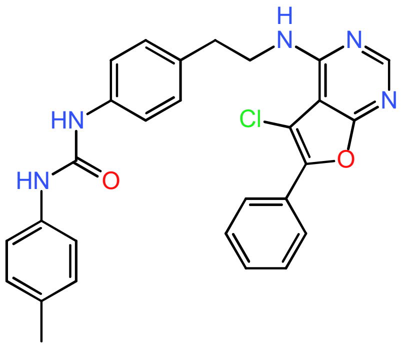

IC50(nM): 73.3
fringNplaN4B: 0
fsp3Csp2N5B: 1
N_H_2B: 5
fsp2Osp2C5B: 4
da_lipo_5B: 16
fringNC6B: 4
fOringC6B: 2

459 2271208

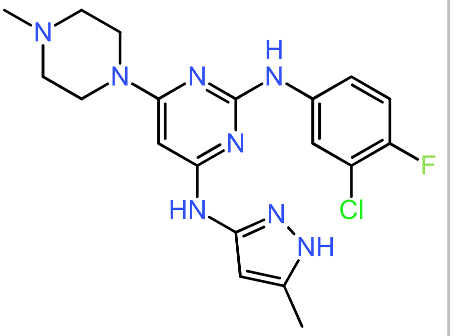

IC50(nM): 25.7
fringNplaN4B: 0
fsp3Csp2N5B: 1
N_H_2B: 6
fsp2Osp2C5B: 0
da_lipo_5B: 10
fringNC6B: 1
fOringC6B: 0

460 2271892

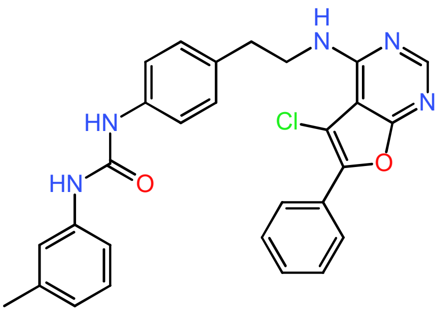

IC50(nM): 74.9
fringNplaN4B: 0
fsp3Csp2N5B: 1
N_H_2B: 5
fsp2Osp2C5B: 4
da_lipo_5B: 16
fringNC6B: 4
fOringC6B: 2

461 2271936

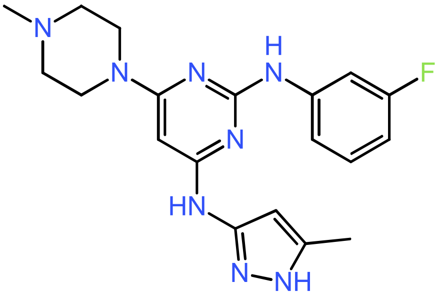

IC50(nM): 102
fringNplaN4B: 0
fsp3Csp2N5B: 1
N_H_2B: 6
fsp2Osp2C5B: 0
da_lipo_5B: 9
fringNC6B: 1
fOringC6B: 0

462 2274951

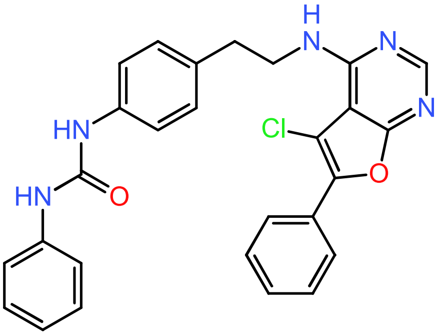

IC50(nM): 128.75
fringNplaN4B: 0
fsp3Csp2N5B: 1
N_H_2B: 5
fsp2Osp2C5B: 4
da_lipo_5B: 15
fringNC6B: 4
fOringC6B: 2

463 2277259

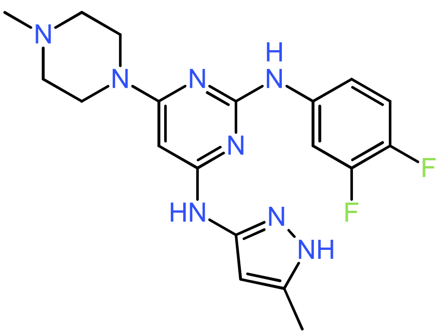

IC50(nM): 89.2
fringNplaN4B: 0
fsp3Csp2N5B: 1
N_H_2B: 6
fsp2Osp2C5B: 0
da_lipo_5B: 10
fringNC6B: 1
fOringC6B: 0

464 2278710

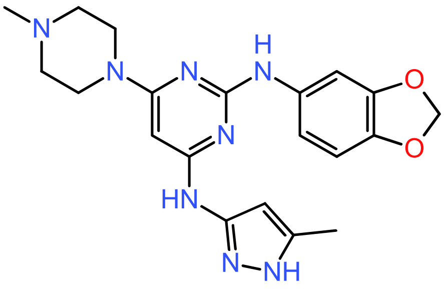

IC50(nM): 91.7
fringNplaN4B: 0
fsp3Csp2N5B: 1
N_H_2B: 6
fsp2Osp2C5B: 0
da_lipo_5B: 6
fringNC6B: 1
fOringC6B: 0

465 2287003

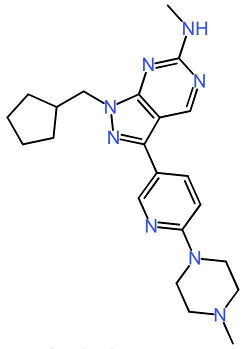

IC50(nM): 170
fringNplaN4B: 0
fsp3Csp2N5B: 0
N_H_2B: 6
fsp2Osp2C5B: 0
da_lipo_5B: 1
fringNC6B: 0
fOringC6B: 0

466 2297672

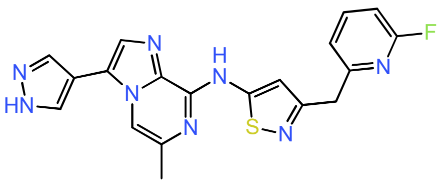

IC50(nM): 56.5
fringNplaN4B: 0
fsp3Csp2N5B: 1
N_H_2B: 5
fsp2Osp2C5B: 0
da_lipo_5B: 4
fringNC6B: 0
fOringC6B: 0

467 2297876

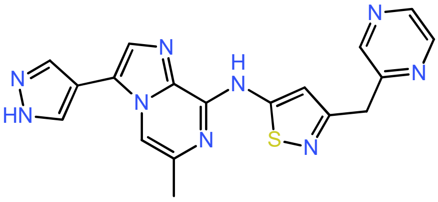

IC50(nM): 110.5
fringNplaN4B: 0
fsp3Csp2N5B: 1
N_H_2B: 7
fsp2Osp2C5B: 0
da_lipo_5B: 4
fringNC6B: 0
fOringC6B: 0

468 2298199

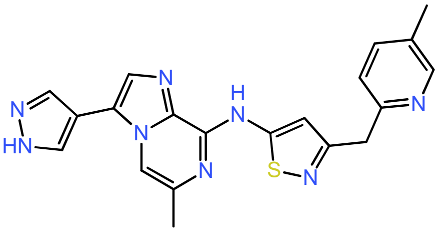

IC50(nM): 244
fringNplaN4B: 0
fsp3Csp2N5B: 1
N_H_2B: 6
fsp2Osp2C5B: 0
da_lipo_5B: 4
fringNC6B: 0
fOringC6B: 0

469 2298414

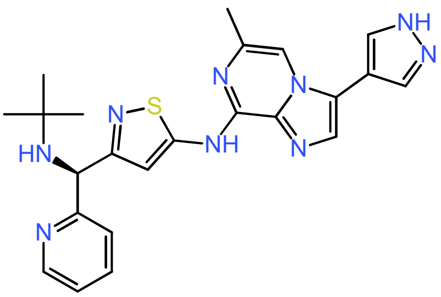

IC50(nM):17.28714286
fringNplaN4B:0
fsp3Csp2N5B:1
N_H_2B:7
fsp2Osp2C5B:0
da_lipo_5B:9
fringNC6B:0
fOringC6B:0

470 2298470

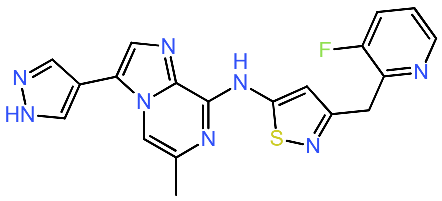

IC50(nM):97.5
fringNplaN4B:0
fsp3Csp2N5B:1
N_H_2B:6
fsp2Osp2C5B:0
da_lipo_5B:4
fringNC6B:0
fOringC6B:0

471 2298527

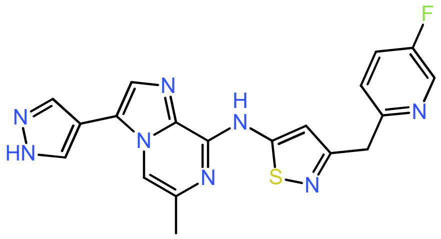

IC50(nM):249.5
fringNplaN4B:0
fsp3Csp2N5B:1
N_H_2B:6
fsp2Osp2C5B:0
da_lipo_5B:4
fringNC6B:0
fOringC6B:0

472 2298675

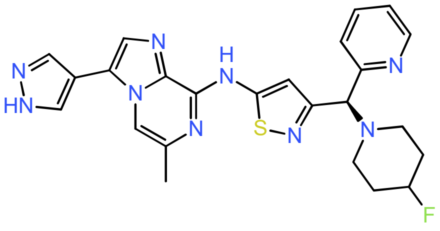

IC50(nM):48
fringNplaN4B:0
fsp3Csp2N5B:1
N_H_2B:7
fsp2Osp2C5B:0
da_lipo_5B:3
fringNC6B:0
fOringC6B:0

473 2298985

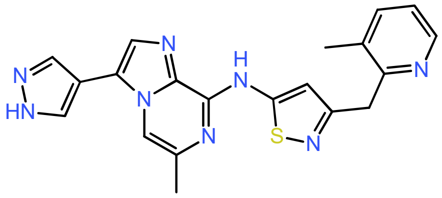

IC50(nM):131.5
fringNplaN4B:0
fsp3Csp2N5B:1
N_H_2B:6
fsp2Osp2C5B:0
da_lipo_5B:4
fringNC6B:0
fOringC6B:0

474 2299148

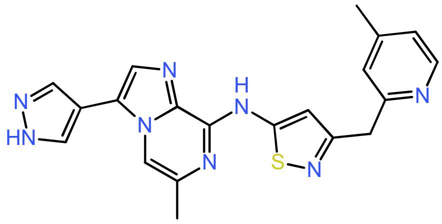

IC50(nM):194.5
fringNplaN4B:0
fsp3Csp2N5B:1
N_H_2B:6
fsp2Osp2C5B:0
da_lipo_5B:4
fringNC6B:0
fOringC6B:0

475 2299195

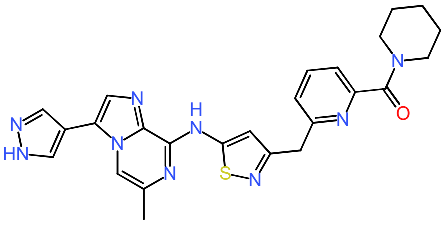

IC50(nM):129
fringNplaN4B:0
fsp3Csp2N5B:1
N_H_2B:6
fsp2Osp2C5B:1
da_lipo_5B:4
fringNC6B:0
fOringC6B:1

476 2299215

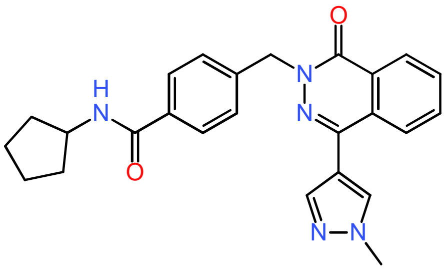

IC50(nM):265
fringNplaN4B:0
fsp3Csp2N5B:0
N_H_2B:4
fsp2Osp2C5B:2
da_lipo_5B:10
fringNC6B:1
fOringC6B:2

477 2299460

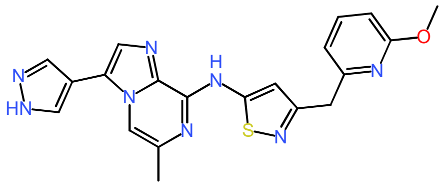

IC50(nM):184.5
fringNplaN4B:0
fsp3Csp2N5B:1
N_H_2B:5
fsp2Osp2C5B:0
da_lipo_5B:4
fringNC6B:0
fOringC6B:1

478 2299573

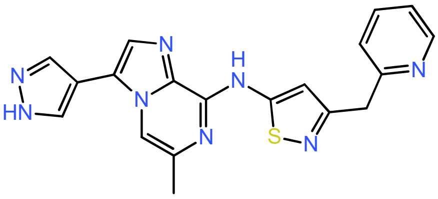

IC50(nM): 43.36666667
fringNplaN4B: 0
fsp3Csp2N5B: 1
N_H_2B: 6
fsp2Osp2C5B: 0
da_lipo_5B: 4
fringNC6B: 0
fOringC6B: 0

479 2300048

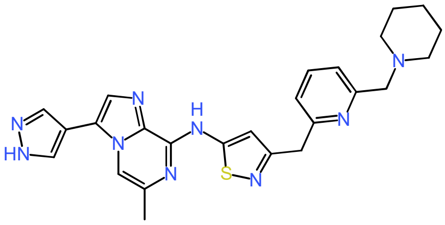

IC50(nM): 151
fringNplaN4B: 0
fsp3Csp2N5B: 1
N_H_2B: 6
fsp2Osp2C5B: 0
da_lipo_5B: 4
fringNC6B: 0
fOringC6B: 0

480 2300295

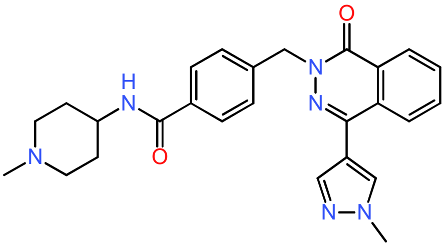

IC50(nM): 105
fringNplaN4B: 1
fsp3Csp2N5B: 0
N_H_2B: 5
fsp2Osp2C5B: 2
da_lipo_5B: 8
fringNC6B: 0
fOringC6B: 2

481 2300309

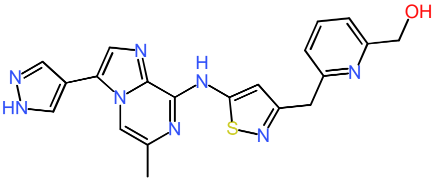

IC50(nM): 286
fringNplaN4B: 0
fsp3Csp2N5B: 1
N_H_2B: 5
fsp2Osp2C5B: 0
da_lipo_5B: 7
fringNC6B: 0
fOringC6B: 1

482 2300335

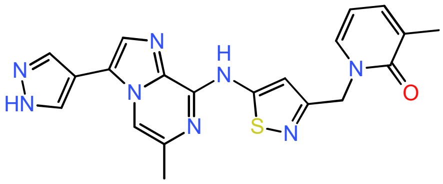

IC50(nM): 250
fringNplaN4B: 0
fsp3Csp2N5B: 1
N_H_2B: 6
fsp2Osp2C5B: 1
da_lipo_5B: 3
fringNC6B: 0
fOringC6B: 1

483 2300558

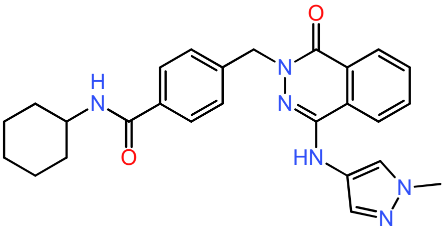

IC50(nM): 132
fringNplaN4B: 0
fsp3Csp2N5B: 0
N_H_2B: 5
fsp2Osp2C5B: 1
da_lipo_5B: 17
fringNC6B: 1
fOringC6B: 2

484 2300626

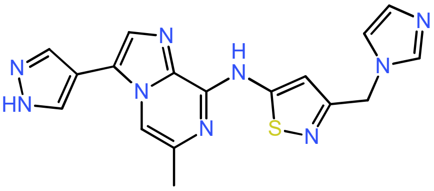

IC50(nM): 191.5
fringNplaN4B: 0
fsp3Csp2N5B: 1
N_H_2B: 7
fsp2Osp2C5B: 0
da_lipo_5B: 3
fringNC6B: 0
fOringC6B: 0

485 2300799

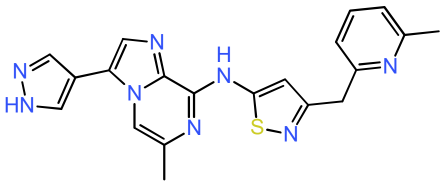

IC50(nM): 50.5
fringNplaN4B: 0
fsp3Csp2N5B: 1
N_H_2B: 5
fsp2Osp2C5B: 0
da_lipo_5B: 4
fringNC6B: 0
fOringC6B: 0

486 2300903

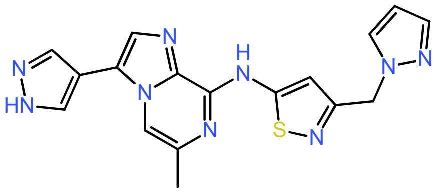

IC50(nM): 98.5
fringNplaN4B: 0
fsp3Csp2N5B: 1
N_H_2B: 7
fsp2Osp2C5B: 0
da_lipo_5B: 3
fringNC6B: 0
fOringC6B: 0

487 2301077

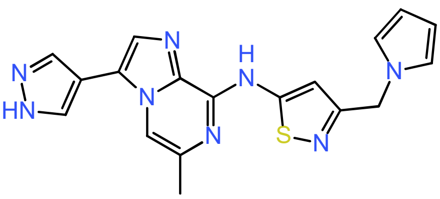

IC50(nM):271.5
fringNplaN4B:0
fsp3Csp2N5B:1
N_H_2B:6
fsp2Osp2C5B:0
da_lipo_5B:3
fringNC6B:0
fOringC6B:0

488 2301078

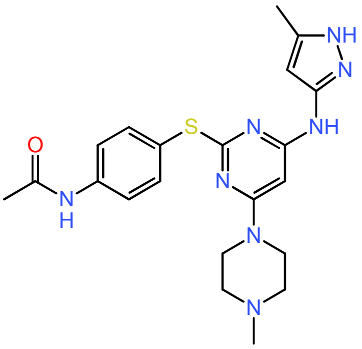

IC50(nM):15
fringNplaN4B:0
fsp3Csp2N5B:1
N_H_2B:6
fsp2Osp2C5B:2
da_lipo_5B:10
fringNC6B:1
fOringC6B:1

489 2301626

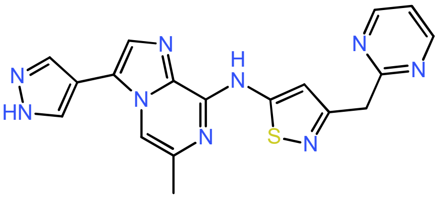

IC50(nM):108
fringNplaN4B:0
fsp3Csp2N5B:1
N_H_2B:7
fsp2Osp2C5B:0
da_lipo_5B:4
fringNC6B:0
fOringC6B:0

490 2303454

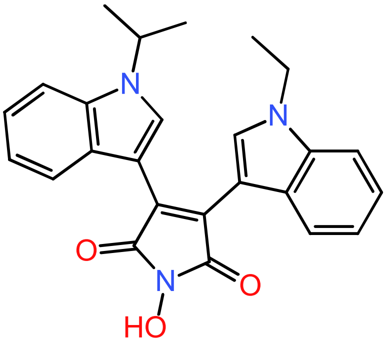

IC50(nM):5047.5
fringNplaN4B:0
fsp3Csp2N5B:0
N_H_2B:3
fsp2Osp2C5B:4
da_lipo_5B:6
fringNC6B:0
fOringC6B:4

491 2303892

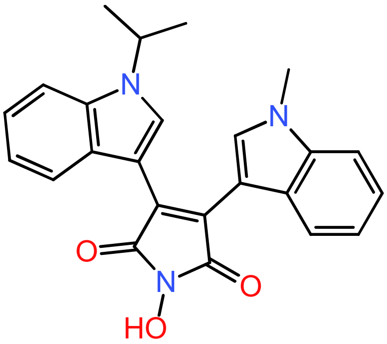

IC50(nM):5047.5
fringNplaN4B:0
fsp3Csp2N5B:0
N_H_2B:3
fsp2Osp2C5B:4
da_lipo_5B:6
fringNC6B:0
fOringC6B:4

492 2305350

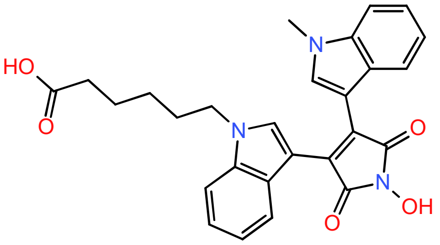

IC50(nM):5035.5
fringNplaN4B:0
fsp3Csp2N5B:0
N_H_2B:3
fsp2Osp2C5B:4
da_lipo_5B:10
fringNC6B:1
fOringC6B:4

493 2306374

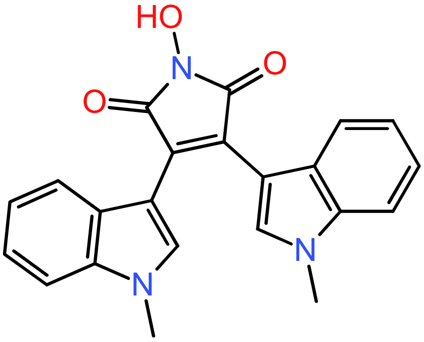

IC50(nM):5042
fringNplaN4B:0
fsp3Csp2N5B:0
N_H_2B:3
fsp2Osp2C5B:4
da_lipo_5B:6
fringNC6B:0
fOringC6B:4

494 2306806

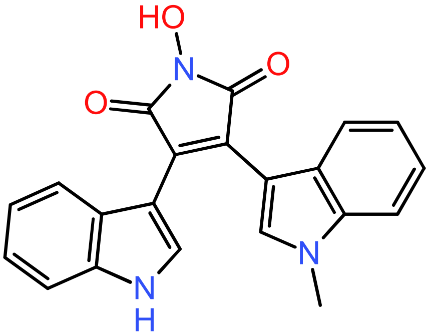

IC50(nM):5039.5
fringNplaN4B:0
fsp3Csp2N5B:0
N_H_2B:3
fsp2Osp2C5B:4
da_lipo_5B:10
fringNC6B:0
fOringC6B:4

495 2307651

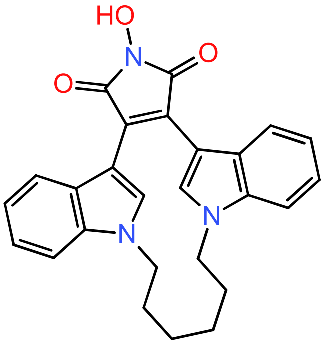

IC50(nM):5048.5
fringNplaN4B:0
fsp3Csp2N5B:0
N_H_2B:3
fsp2Osp2C5B:4
da_lipo_5B:6
fringNC6B:0
fOringC6B:4

496 2310161

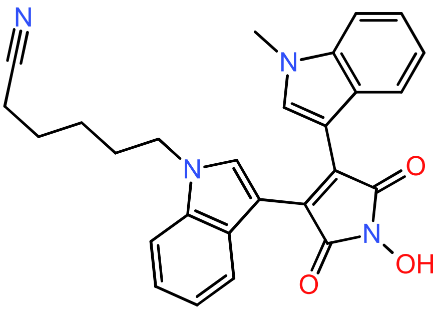

IC50(nM):5035.5
fringNplaN4B:0
fsp3Csp2N5B:0
N_H_2B:3
fsp2Osp2C5B:4
da_lipo_5B:6
fringNC6B:1
fOringC6B:4

497 2310633

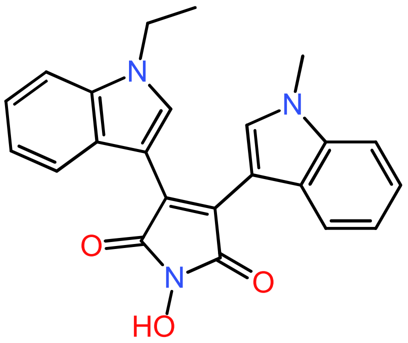

IC50(nM):5049
fringNplaN4B:0
fsp3Csp2N5B:0
N_H_2B:3
fsp2Osp2C5B:4
da_lipo_5B:6
fringNC6B:0
fOringC6B:4

498 2313964

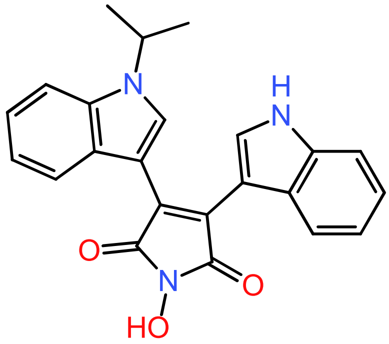

IC50(nM):5073.5
fringNplaN4B:0
fsp3Csp2N5B:0
N_H_2B:3
fsp2Osp2C5B:4
da_lipo_5B:10
fringNC6B:0
fOringC6B:4

499 2317141

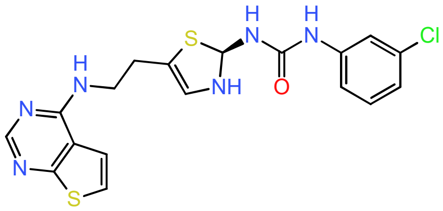

IC50(nM):31
fringNplaN4B:1
fsp3Csp2N5B:1
N_H_2B:6
fsp2Osp2C5B:4
da_lipo_5B:13
fringNC6B:2
fOringC6B:1

500 2317838

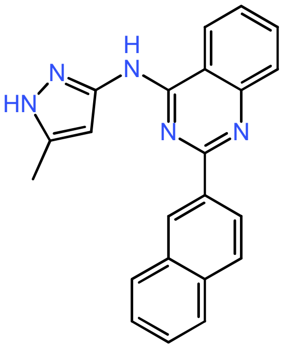

IC50(nM):5070
fringNplaN4B:0
fsp3Csp2N5B:0
N_H_2B:3
fsp2Osp2C5B:0
da_lipo_5B:10
fringNC6B:2
fOringC6B:0

501 2318132

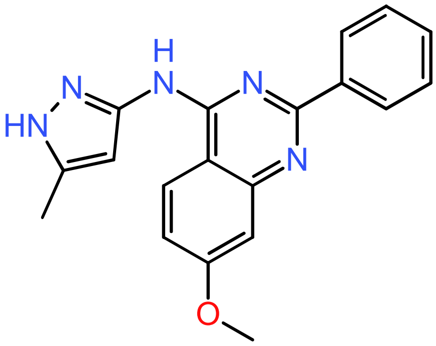

IC50(nM):773
fringNplaN4B:0
fsp3Csp2N5B:1
N_H_2B:3
fsp2Osp2C5B:0
da_lipo_5B:9
fringNC6B:0
fOringC6B:1

502 2318210

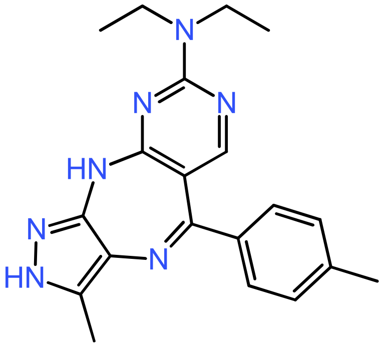

IC50(nM):321.6
fringNplaN4B:0
fsp3Csp2N5B:0
N_H_2B:5
fsp2Osp2C5B:0
da_lipo_5B:5
fringNC6B:1
fOringC6B:0

503 2319570

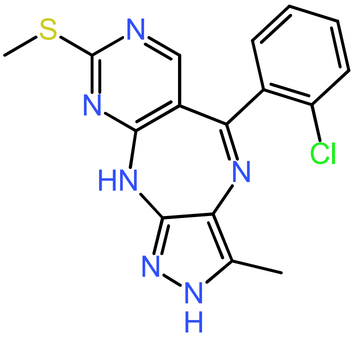

IC50(nM):278.1
fringNplaN4B:0
fsp3Csp2N5B:0
N_H_2B:4
fsp2Osp2C5B:0
da_lipo_5B:7
fringNC6B:0
fOringC6B:0

504 2320173

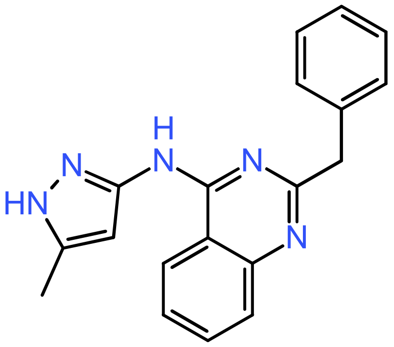

IC50(nM):1271
fringNplaN4B:0
fsp3Csp2N5B:0
N_H_2B:3
fsp2Osp2C5B:0
da_lipo_5B:9
fringNC6B:1
fOringC6B:0

505 2321271

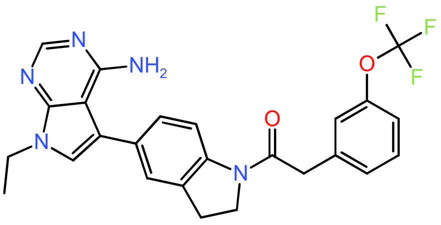

IC50(nM):210
fringNplaN4B:0
fsp3Csp2N5B:1
N_H_2B:5
fsp2Osp2C5B:4
da_lipo_5B:5
fringNC6B:1
fOringC6B:1

506 2321294

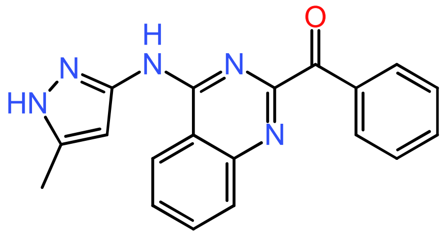

IC50(nM):11500
fringNplaN4B:0
fsp3Csp2N5B:0
N_H_2B:3
fsp2Osp2C5B:3
da_lipo_5B:8
fringNC6B:1
fOringC6B:3

507 2321596

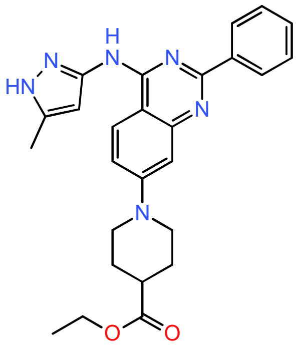

IC50(nM):2522
fringNplaN4B:0
fsp3Csp2N5B:1
N_H_2B:4
fsp2Osp2C5B:0
da_lipo_5B:9
fringNC6B:1
fOringC6B:1

508 2321921

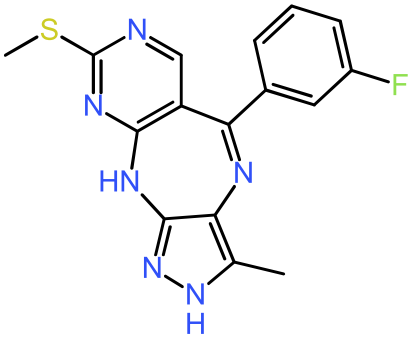

IC50(nM):611
fringNplaN4B:0
fsp3Csp2N5B:0
N_H_2B:4
fsp2Osp2C5B:0
da_lipo_5B:7
fringNC6B:0
fOringC6B:0

509 2321978

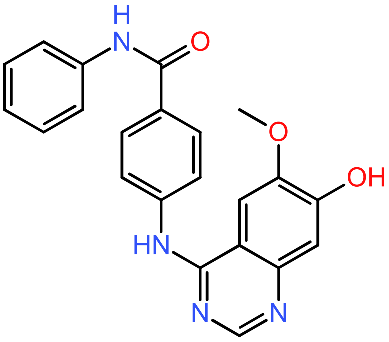

IC50(nM):41
fringNplaN4B:0
fsp3Csp2N5B:0
N_H_2B:4
fsp2Osp2C5B:3
da_lipo_5B:13
fringNC6B:2
fOringC6B:1

510 2322312

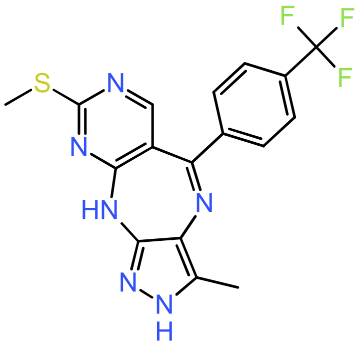

IC50(nM):854.4
fringNplaN4B:0
fsp3Csp2N5B:0
N_H_2B:4
fsp2Osp2C5B:0
da_lipo_5B:7
fringNC6B:1
fOringC6B:0

511 2322741

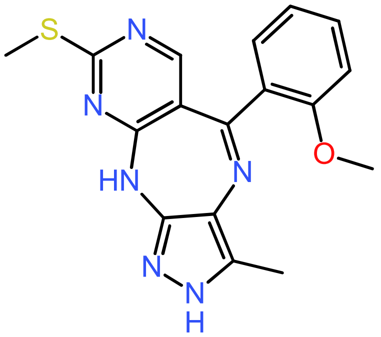

IC50(nM):2320.6
fringNplaN4B:0
fsp3Csp2N5B:0
N_H_2B:4
fsp2Osp2C5B:0
da_lipo_5B:6
fringNC6B:0
fOringC6B:2

512 2325459

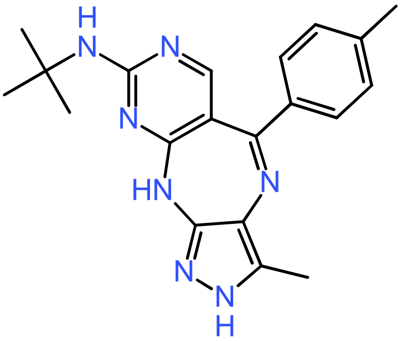

IC50(nM):1184.7
fringNplaN4B:0
fsp3Csp2N5B:0
N_H_2B:5
fsp2Osp2C5B:0
da_lipo_5B:8
fringNC6B:1
fOringC6B:0

513 2326090

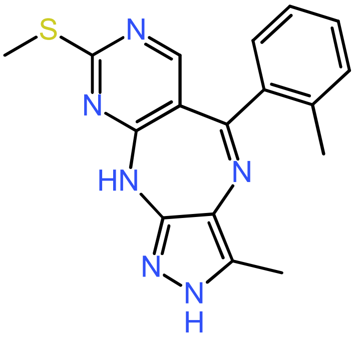

IC50(nM):610.5
fringNplaN4B:0
fsp3Csp2N5B:0
N_H_2B:4
fsp2Osp2C5B:0
da_lipo_5B:7
fringNC6B:0
fOringC6B:0

514 2326870

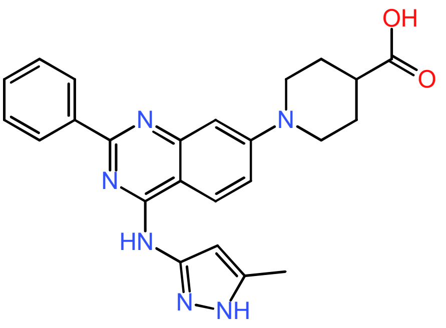

IC50(nM): 127
fringNplaN4B: 0
fsp3Csp2N5B: 1
N_H_2B: 4
fsp2Osp2C5B: 0
da_lipo_5B: 12
fringNC6B: 0
fOringC6B: 1

515 2328465

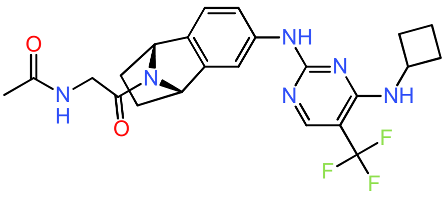

IC50(nM): 17.75
fringNplaN4B: 0
fsp3Csp2N5B: 0
N_H_2B: 5
fsp2Osp2C5B: 2
da_lipo_5B: 16
fringNC6B: 0
fOringC6B: 2

516 2328510

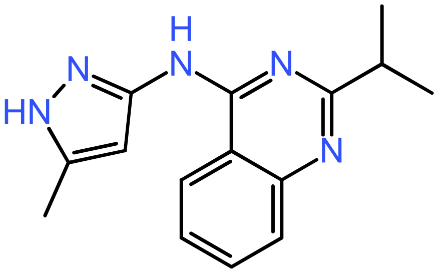

IC50(nM): 1147
fringNplaN4B: 0
fsp3Csp2N5B: 0
N_H_2B: 3
fsp2Osp2C5B: 0
da_lipo_5B: 10
fringNC6B: 0
fOringC6B: 0

517 2328881

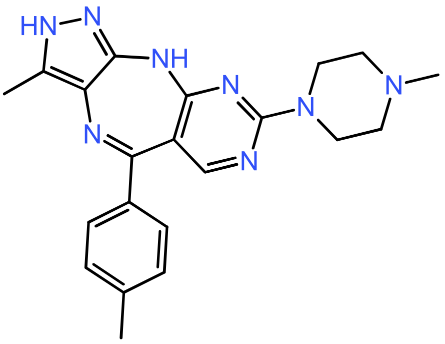

IC50(nM): 86
fringNplaN4B: 0
fsp3Csp2N5B: 0
N_H_2B: 6
fsp2Osp2C5B: 0
da_lipo_5B: 5
fringNC6B: 1
fOringC6B: 0

518 2329580

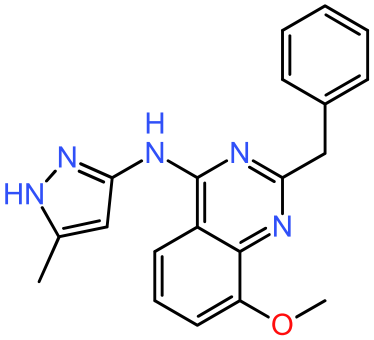

IC50(nM): 893
fringNplaN4B: 0
fsp3Csp2N5B: 0
N_H_2B: 3
fsp2Osp2C5B: 0
da_lipo_5B: 8
fringNC6B: 1
fOringC6B: 2

519 2329793

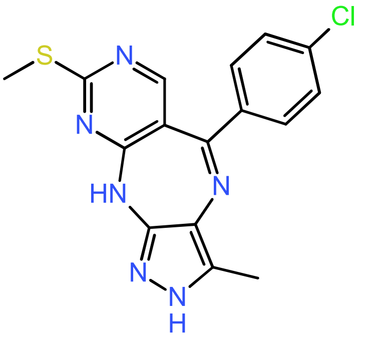

IC50(nM): 1620
fringNplaN4B: 0
fsp3Csp2N5B: 0
N_H_2B: 4
fsp2Osp2C5B: 0
da_lipo_5B: 7
fringNC6B: 0
fOringC6B: 0

520 2330206

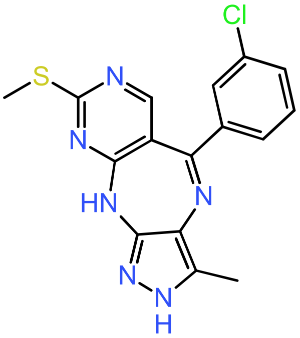

IC50(nM): 1003.6
fringNplaN4B: 0
fsp3Csp2N5B: 0
N_H_2B: 4
fsp2Osp2C5B: 0
da_lipo_5B: 7
fringNC6B: 0
fOringC6B: 0

521 2330594

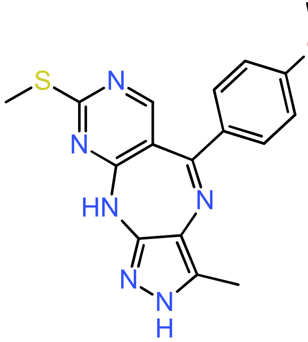

IC50(nM): 2681.5
fringNplaN4B: 0
fsp3Csp2N5B: 0
N_H_2B: 4
fsp2Osp2C5B: 0
da_lipo_5B: 7
fringNC6B: 0
fOringC6B: 1

522 2331020

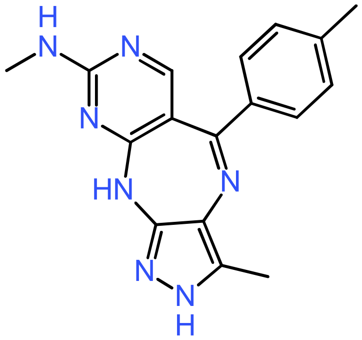

IC50(nM): 352
fringNplaN4B: 0
fsp3Csp2N5B: 0
N_H_2B: 5
fsp2Osp2C5B: 0
da_lipo_5B: 5
fringNC6B: 1
fOringC6B: 0

523 2331922

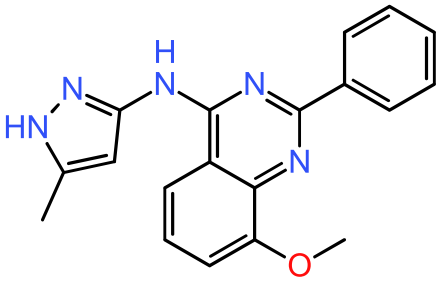

IC50(nM):547
fringNplaN4B:0
fsp3Csp2N5B:0
N_H_2B:3
fsp2Osp2C5B:0
da_lipo_5B:9
fringNC6B:0
fOringC6B:3

524 2332203

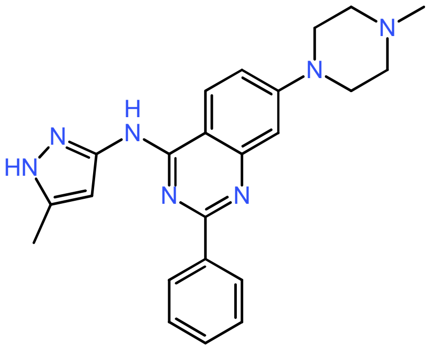

IC50(nM):270
fringNplaN4B:0
fsp3Csp2N5B:1
N_H_2B:5
fsp2Osp2C5B:0
da_lipo_5B:9
fringNC6B:0
fOringC6B:0

525 2332915

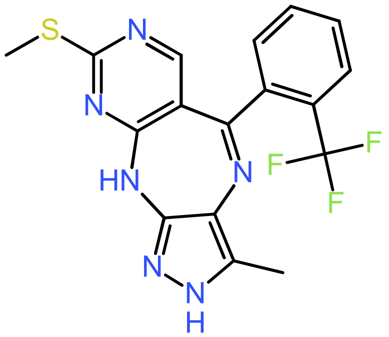

IC50(nM):307.4
fringNplaN4B:0
fsp3Csp2N5B:0
N_H_2B:4
fsp2Osp2C5B:0
da_lipo_5B:7
fringNC6B:0
fOringC6B:0

526 2333609

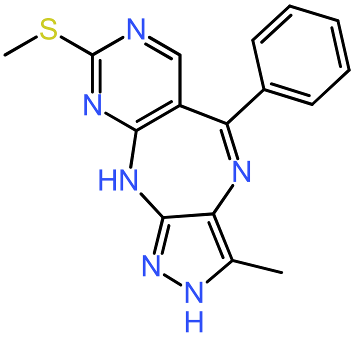

IC50(nM):894
fringNplaN4B:0
fsp3Csp2N5B:0
N_H_2B:4
fsp2Osp2C5B:0
da_lipo_5B:7
fringNC6B:0
fOringC6B:0

527 2341713

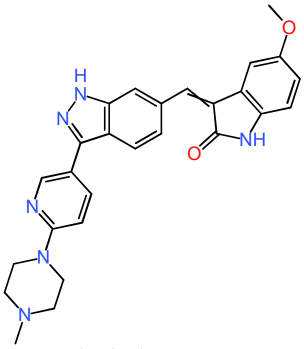

IC50(nM):35
fringNplaN4B:0
fsp3Csp2N5B:0
N_H_2B:6
fsp2Osp2C5B:4
da_lipo_5B:14
fringNC6B:1
fOringC6B:2

528 2343585

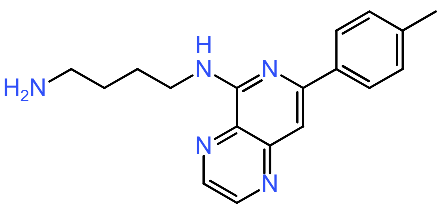

IC50(nM):1258.9
fringNplaN4B:0
fsp3Csp2N5B:1
N_H_2B:4
fsp2Osp2C5B:0
da_lipo_5B:6
fringNC6B:2
fOringC6B:0

529 2344424

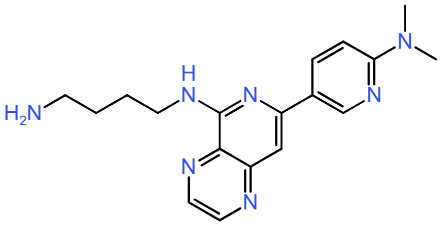

IC50(nM):50.12
fringNplaN4B:0
fsp3Csp2N5B:1
N_H_2B:6
fsp2Osp2C5B:0
da_lipo_5B:5
fringNC6B:1
fOringC6B:0

530 2345757

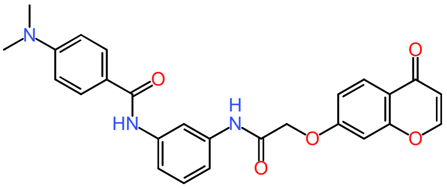

IC50(nM):1239
fringNplaN4B:0
fsp3Csp2N5B:0
N_H_2B:3
fsp2Osp2C5B:2
da_lipo_5B:11
fringNC6B:0
fOringC6B:0

531 2346337

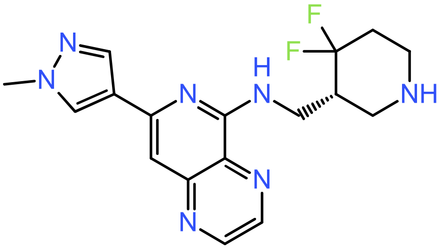

IC50(nM):63.1
fringNplaN4B:0
fsp3Csp2N5B:1
N_H_2B:6
fsp2Osp2C5B:0
da_lipo_5B:7
fringNC6B:0
fOringC6B:0

532 2346662

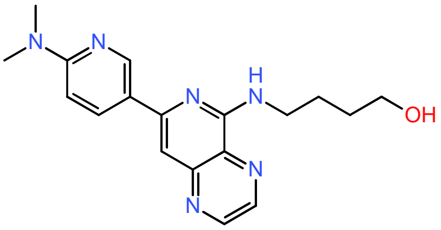

IC50(nM):100
fringNplaN4B:0
fsp3Csp2N5B:1
N_H_2B:5
fsp2Osp2C5B:0
da_lipo_5B:5
fringNC6B:1
fOringC6B:1

533 2347062

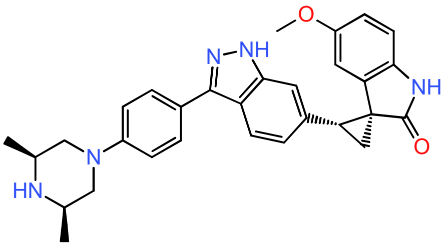

IC50(nM):13
fringNplaN4B:0
fsp3Csp2N5B:1
N_H_2B:5
fsp2Osp2C5B:4
da_lipo_5B:19
fringNC6B:1
fOringC6B:2

534 2347821

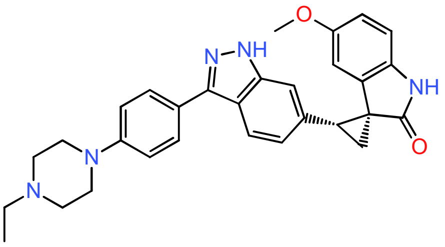

IC50(nM):8.5
fringNplaN4B:0
fsp3Csp2N5B:1
N_H_2B:5
fsp2Osp2C5B:4
da_lipo_5B:17
fringNC6B:1
fOringC6B:2

535 2351542

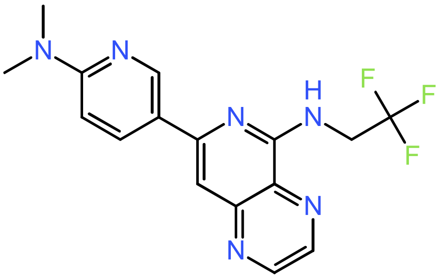

IC50(nM):50.12
fringNplaN4B:0
fsp3Csp2N5B:1
N_H_2B:5
fsp2Osp2C5B:0
da_lipo_5B:7
fringNC6B:0
fOringC6B:0

536 2353443

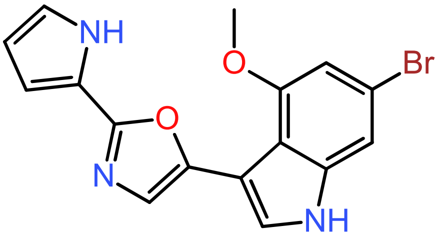

IC50(nM):900
fringNplaN4B:0
fsp3Csp2N5B:0
N_H_2B:3
fsp2Osp2C5B:0
da_lipo_5B:8
fringNC6B:0
fOringC6B:0

537 2355605

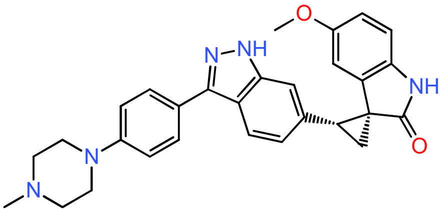

IC50(nM):6.9
fringNplaN4B:0
fsp3Csp2N5B:1
N_H_2B:5
fsp2Osp2C5B:4
da_lipo_5B:17
fringNC6B:1
fOringC6B:2

538 2355704

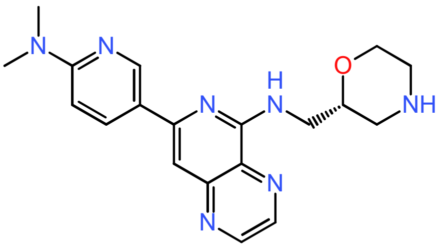

IC50(nM):89.715
fringNplaN4B:0
fsp3Csp2N5B:1
N_H_2B:6
fsp2Osp2C5B:0
da_lipo_5B:3
fringNC6B:0
fOringC6B:2

539 2360227

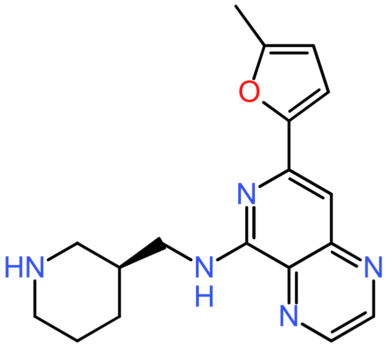

IC50(nM):630.96
fringNplaN4B:0
fsp3Csp2N5B:1
N_H_2B:4
fsp2Osp2C5B:0
da_lipo_5B:5
fringNC6B:0
fOringC6B:1

540 2362102

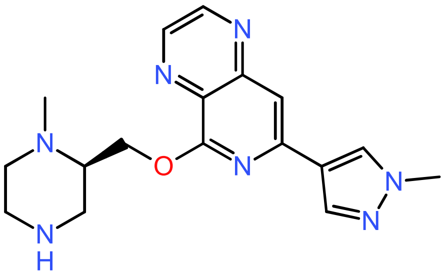

IC50(nM):630.96
fringNplaN4B:0
fsp3Csp2N5B:1
N_H_2B:6
fsp2Osp2C5B:0
da_lipo_5B:0
fringNC6B:0
fOringC6B:0

541 2362277

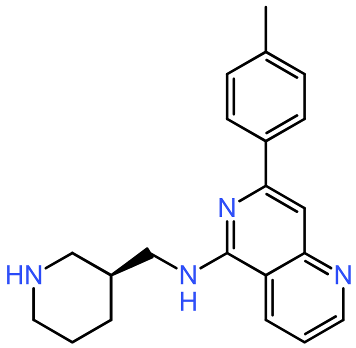

IC50(nM):1584.9
fringNplaN4B:0
fsp3Csp2N5B:1
N_H_2B:3
fsp2Osp2C5B:0
da_lipo_5B:10
fringNC6B:1
fOringC6B:0

542 2365854

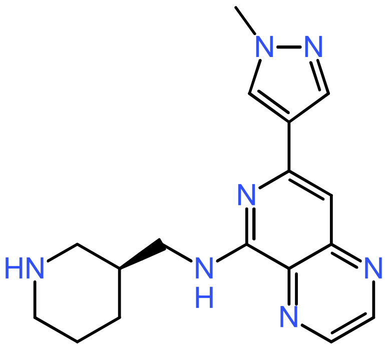

IC50(nM):251.19
fringNplaN4B:0
fsp3Csp2N5B:1
N_H_2B:6
fsp2Osp2C5B:0
da_lipo_5B:5
fringNC6B:0
fOringC6B:0

543 2367292

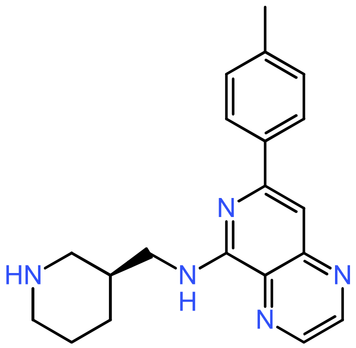

IC50(nM):630.96
fringNplaN4B:0
fsp3Csp2N5B:1
N_H_2B:4
fsp2Osp2C5B:0
da_lipo_5B:7
fringNC6B:1
fOringC6B:0

544 2367685

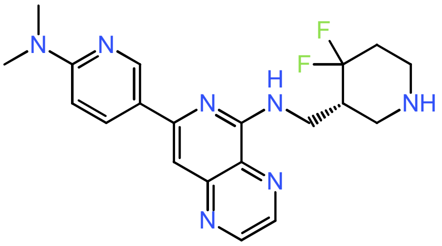

IC50(nM):89.715
fringNplaN4B:0
fsp3Csp2N5B:1
N_H_2B:6
fsp2Osp2C5B:0
da_lipo_5B:8
fringNC6B:0
fOringC6B:0

545 2375056

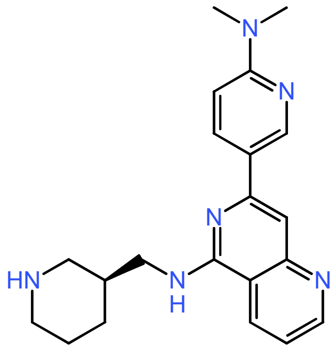

IC50(nM):316.23
fringNplaN4B:0
fsp3Csp2N5B:1
N_H_2B:5
fsp2Osp2C5B:0
da_lipo_5B:9
fringNC6B:0
fOringC6B:0

546 2376550

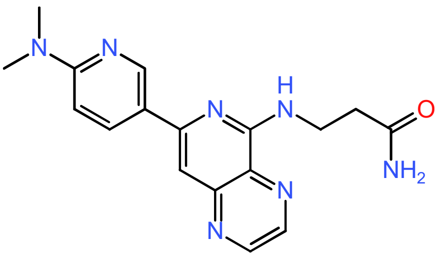

IC50(nM):79.43
fringNplaN4B:0
fsp3Csp2N5B:1
N_H_2B:6
fsp2Osp2C5B:1
da_lipo_5B:4
fringNC6B:0
fOringC6B:1

547 2379836

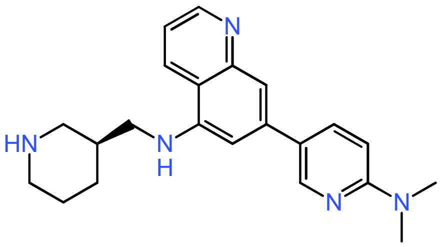

IC50(nM):100
fringNplaN4B:1
fsp3Csp2N5B:1
N_H_2B:5
fsp2Osp2C5B:0
da_lipo_5B:11
fringNC6B:0
fOringC6B:0

548 2380159

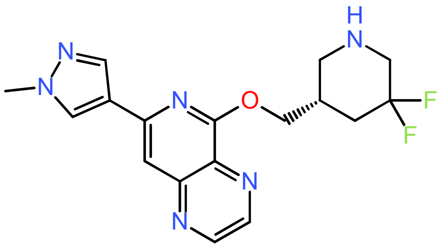

IC50(nM):63.1
fringNplaN4B:0
fsp3Csp2N5B:1
N_H_2B:5
fsp2Osp2C5B:0
da_lipo_5B:5
fringNC6B:0
fOringC6B:0

549 2385709

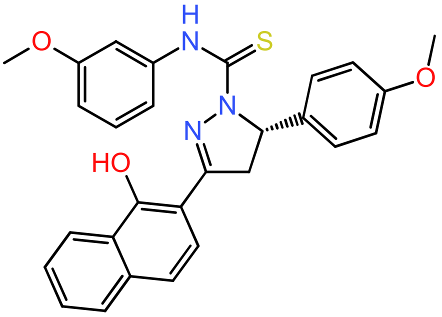

IC50(nM):8530
fringNplaN4B:0
fsp3Csp2N5B:0
N_H_2B:2
fsp2Osp2C5B:0
da_lipo_5B:18
fringNC6B:3
fOringC6B:0

550 2391808

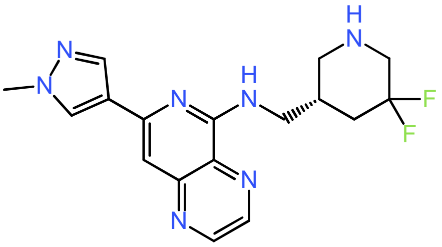

IC50(nM): 501.19
fringNplaN4B: 0
fsp3Csp2N5B: 1
N_H_2B: 6
fsp2Osp2C5B: 0
da_lipo_5B: 7
fringNC6B: 0
fOringC6B: 0

551 2394312

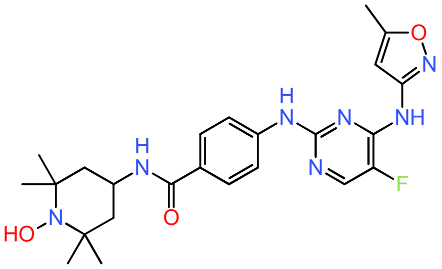

IC50(nM): 46.9
fringNplaN4B: 1
fsp3Csp2N5B: 0
N_H_2B: 5
fsp2Osp2C5B: 1
da_lipo_5B: 15
fringNC6B: 1
fOringC6B: 2

552 2396581

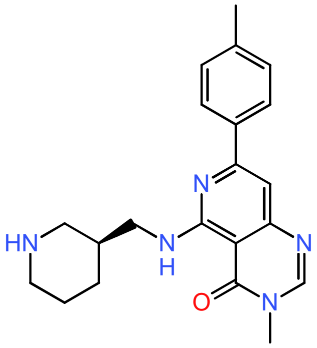

IC50(nM): 199.53
fringNplaN4B: 0
fsp3Csp2N5B: 0
N_H_2B: 4
fsp2Osp2C5B: 1
da_lipo_5B: 8
fringNC6B: 1
fOringC6B: 2

553 2398720

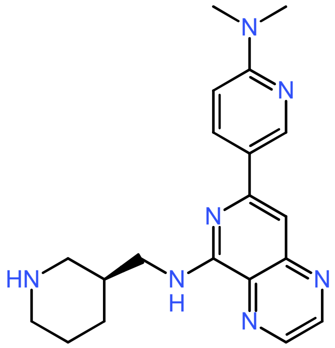

IC50(nM): 251.19
fringNplaN4B: 0
fsp3Csp2N5B: 1
N_H_2B: 6
fsp2Osp2C5B: 0
da_lipo_5B: 6
fringNC6B: 0
fOringC6B: 0

554 2420095

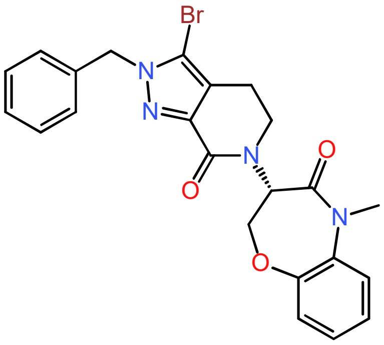

IC50(nM): 1000
fringNplaN4B: 0
fsp3Csp2N5B: 0
N_H_2B: 3
fsp2Osp2C5B: 2
da_lipo_5B: 0
fringNC6B: 0
fOringC6B: 1

555 2423779

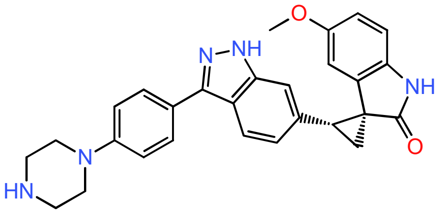

IC50(nM): 6.9
fringNplaN4B: 0
fsp3Csp2N5B: 1
N_H_2B: 5
fsp2Osp2C5B: 4
da_lipo_5B: 17
fringNC6B: 1
fOringC6B: 2

556 2431391

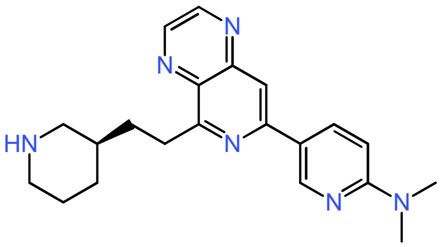

IC50(nM): 158.49
fringNplaN4B: 0
fsp3Csp2N5B: 0
N_H_2B: 5
fsp2Osp2C5B: 0
da_lipo_5B: 5
fringNC6B: 0
fOringC6B: 0

557 2431526

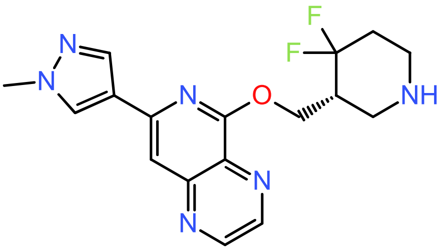

IC50(nM): 50.12
fringNplaN4B: 0
fsp3Csp2N5B: 1
N_H_2B: 5
fsp2Osp2C5B: 0
da_lipo_5B: 5
fringNC6B: 0
fOringC6B: 0

558 2434765

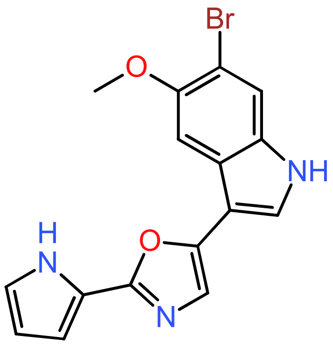

IC50(nM): 222.25
fringNplaN4B: 0
fsp3Csp2N5B: 0
N_H_2B: 3
fsp2Osp2C5B: 0
da_lipo_5B: 8
fringNC6B: 1
fOringC6B: 0

559 2440716

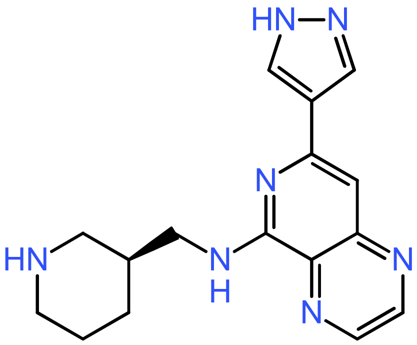

IC50(nM): 398.11
fringNplaN4B: 0
fsp3Csp2N5B: 1
N_H_2B: 6
fsp2Osp2C5B: 0
da_lipo_5B: 5
fringNC6B: 0
fOringC6B: 0

560 2455220

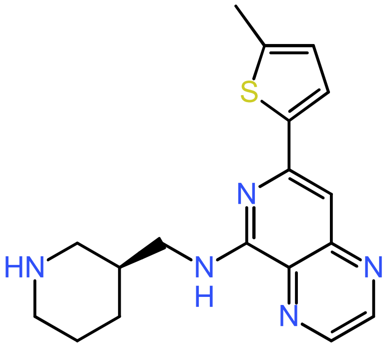

IC50(nM): 316.23
fringNplaN4B: 0
fsp3Csp2N5B: 1
N_H_2B: 4
fsp2Osp2C5B: 0
da_lipo_5B: 7
fringNC6B: 0
fOringC6B: 0

561 2460851

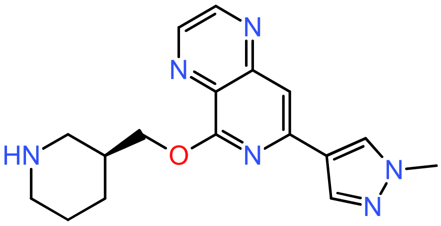

IC50(nM): 158.49
fringNplaN4B: 0
fsp3Csp2N5B: 1
N_H_2B: 5
fsp2Osp2C5B: 0
da_lipo_5B: 3
fringNC6B: 0
fOringC6B: 0
