# Supplementary material for: Pharmacophore Synergism in Diverse Scaffold Clinches in Aurora Kinase B
Source: Int J Mol Sci. 2022 Nov 22;23(23):14527. doi: 10.3390/ijms232314527 (PMC9739353; doi:10.3390/ijms232314527)
Supplement: Supplementary file 1 [file ijms-23-14527-s001.zip › Supplementary Materials.pdf]

Pharmacophore Synergism in Diverse Scaffolds Clinches Aurora Kinase B

Vijay H. Masand [1,*], Sami A. Al-Hussain [2], Mithilesh M. Rathore [1], Sumer D. Thakur [3], Siddhartha Akasapu [4], Abdul Samad [5], Aamal A. Al-Mutairi [2] and Magdi E. A. Zaki [2,*]

Statistical Parameters for Model-A:

R^2_{tr} = 0.815, $R^2_{adj.}$ = 0.812, R^2_{tr} - $R^2_{adj.}$ = 0.003, *LOF* = 0.234, *Kxx* = 0.409, *ΔK* = 0.045, $RMSE_{tr}$ = 0.468, MAE_{tr} = 0.401, RSS_{tr} = 98.506, CCC_{tr} = 0.898, *s* = 0.473, *F* = 277.836, R^2_{cv} (Q^2loo) = 0.808, R^2-R^2_{cv} = 0.007, $RMSE_{cv}$ = 0.477, MAE_{cv} = 0.408, $PRESS_{cv}$ = 102.107, CCC_{cv} = 0.895, Q^2_{LMO} = 0.807, R^2_{Yscr} = 0.016, Q^2_{Yscr} = -0.02, $RMSE_{ex}$ = 0.446, MAE_{ex} = 0.373, $PRESS_{ext}$ = 22.264, R^2_{ex} = 0.814, Q^2-F^1 = 0.811, Q^2-F^2 = 0.811, Q^2-F^3 = 0.833, CCC_{ex} = 0.9, R^2-$ExPy$ = 0.814, R'^2_o = 0.79, *k'* = 0.991, $1-(R^2/R'^2_o)$ = 0.029, r'^2m = 0.689, R_o^2 = 0.812, *k* = 1.005, $1-(R^2\text{-}ExPy/R_o^2)$ = 0.002, r^2m = 0.779,

Table S1. A comparison of two tautomeric forms YJA-T1 and YJA-T2 with respect to interactions with the receptor.

Tautomer with descriptors value	H-bonds formed with distance (Å) with angle (Donor-Hydrogen-Acceptor) (cut-off: 5 Å)	**List of amino acids and solvent molecules whose at least one atom is within 5 Å from tautomeric form**
YJA-T1<br>fsp3Csp2N5B = 0<br>N_H_2B = 6<br>fsp2Osp2C5B = 3	LYS122 at 1.668 with 159.8°, GLN145 at 2.251 with 142.4, ALA173 at 1.952 with 163.9°	**ARG97**, LEU99, PHE104, VAL107, ALA120, LYS122, LEU124, LEU138, GLU141, ILE142, GLN145, LEU154, MET156, LEU168, LEU170, PHE172, ALA173, GLU177, LEU223, ALA233, **ASP234**, **HOH2016**
YJA-T2<br>fsp3Csp2N5B = 1<br>N_H_2B = 7<br>fsp2Osp2C5B = 3	LYS122 at 2.361 with 157.8°, GLN145 at 2.323 with 115.7°, ALA173 at 1.946 with 174.4°, HOH2108 2.222 with 106.7°	LEU99, PHE104, VAL107, ALA120, LYS122, LEU124, LEU138, GLU141, ILE142, GLN145, LEU154, MET156, LEU168, LEU170, PHE172, ALA173, **ARG175**, GLU177, **LYS180**, LEU223, ALA233, **HOH2060**, **HOH2108**

Statistical parameters used for validation of QSAR models:

$$R^2 = 1 - \frac{\sum(y_i - \hat{y}_i)^2}{\sum(y_i - \bar{y})^2}$$

where y_i are the observed values of the response, $\bar{y}$ the corresponding average, $\hat{y}$ are the calculated values

$$Q^2 = 1 - \frac{\sum(y_i - \hat{y}_i)^2}{\sum(y_i - \bar{y})^2}$$

where y_i are the observed values of the response, $\bar{y}$ the corresponding average, $\hat{y}$ are the values predicted for each object when it is not in the training set.

$$Q_{F1}^2 = 1 - \frac{\sum_{i=1}^{n_{EXT}}(y_i - \hat{y}_i)^2}{\sum_{i=1}^{n_{EXT}}(y_i - \bar{y}_{TR})^2}$$

where y_i are the observed values of the response, $\bar{y}$ the corresponding average, $\hat{y}$ are the calculated values

$$Q_{F2}^2 = 1 - \frac{\sum_{i=1}^{n_{EXT}}(y_i - \hat{y}_i)^2}{\sum_{i=1}^{n_{EXT}}(y_i - \bar{y}_{EXT})^2}$$

where y_i are the observed values of the response, $\bar{y}$ the corresponding average, $\hat{y}$ are the calculated values

$$Q_{F3}^2 = 1 - \frac{\left[\sum_{i=1}^{n_{EXT}}(y_i - \hat{y}_i)^2\right]/n_{EXT}}{\left[\sum_{i=1}^{n_{TR}}(y_i - \bar{y}_{TR})^2\right]/n_{TR}}$$

where y_i are the observed values of the response, $\bar{y}$ the corresponding average, $\hat{y}$ are the calculated values

$$CCC = \frac{2\sum_{i=1}^{n_{EXT}}(y_i - \bar{y})(\hat{y}_i - \bar{\hat{y}})}{\sum_{i=1}^{n_{EXT}}(y_i - \bar{y})^2 + \sum_{i=1}^{n_{EXT}}(\hat{y}_i - \bar{\hat{y}})^2 + n_{EXT}(\bar{y} - \bar{\hat{y}})^2}$$

$$k = \frac{\sum_{i=i}^{n_{EXT}} y_i\hat{y}_i}{\sum_{i=1}^{n_{EXT}} \hat{y}_i^2}$$

$$k' = \frac{\sum_{i=i}^{n_{EXT}} y_i\hat{y}_i}{\sum_{i=1}^{n_{EXT}} y_i^2}$$

$$r_m^2 = r^2\left(1 - \sqrt{r^2 - r_0^2}\right)$$

$$\overline{r_m^2} = \frac{(r_m^2 + r_m'^2)}{2}$$

$$RMSE = \sqrt{\frac{\sum_{i=1}^{n_{EXT}}(y_i - \hat{y}_i)^2}{n_{EXT}}}$$

$$MAE = \frac{\sum_{i=1}^{n_{EXT}}|y_i - \hat{y}_i|}{n_{EXT}}$$

where y_i are the observed values of the response, $\bar{y}$ the corresponding average, $\hat{y}$ are the calculated values
